# Identification, introgression, and validation of fruit volatile QTLs from a red-fruited wild tomato species

**DOI:** 10.1093/jxb/erw455

**Published:** 2016-12-31

**Authors:** José L. Rambla, Aurora Medina, Asun Fernández-del-Carmen, Walter Barrantes, Silvana Grandillo, Maria Cammareri, Gloria López-Casado, Guillermo Rodrigo, Arancha Alonso, Santiago García-Martínez, Jaime Primo, Juan J. Ruiz, Rafael Fernández-Muñoz, Antonio J. Monforte, Antonio Granell

**Affiliations:** 1 CSIC-Universidad Politécnica de Valencia, Instituto de Biología Molecular y Celular de Plantas, Valencia 46022, Spain; 2 National Research Council of Italy, Institute of Biosciences and Bioresources (CNR-IBBR), Research Division Portici, Via Università 133, 80055 Portici (Naples), Italy; 3 Universidad Politécnica de Valencia, Centro de Ecología Química Agrícola, Instituto Agroforestal Mediterráneo, Valencia 46022, Spain; 4 CSIC-Universidad de Málaga, Instituto de Hortofruticultura Subtropical y Mediterránea, Algarrobo Costa, Málaga 29750, Spain; 5 Departamento de Biología Aplicada, EPSO-UMH. Ctra, Beniel Km 3,2, Orihuela, Alicante 03312, Spain

**Keywords:** Fruit flavor, introgression lines (ILs), quantitative trait loci (QTLs), recombinant inbred lines (RILs), *Solanum habrochaites*, *Solanum pimpinellifolium*, SolCap tomato SNP array, tomato, volatiles.

## Abstract

Volatile organic compounds (VOCs) are major determinants of fruit flavor, a primary objective in tomato breeding. A recombinant inbred line (RIL) population consisting of 169 lines derived from a cross between *Solanum lycopersicum* and a red-fruited wild tomato species *Solanum pimpinellifolium* accession (SP) was characterized for VOCs in three different seasons. Correlation and hierarchical cluster analyses were performed on the 52 VOCs identified, providing a tool for the putative assignation of individual compounds to metabolic pathways. Quantitative trait locus (QTL) analysis, based on a genetic linkage map comprising 297 single nucleotide polymorphisms (SNPs), revealed 102 QTLs (75% not described previously) corresponding to 39 different VOCs. The SP alleles exerted a positive effect on most of the underlying apocarotenoid volatile QTLs—regarded as desirable for liking tomato—indicating that alleles inherited from SP are a valuable resource for flavor breeding. An introgression line (IL) population developed from the same parental genotypes provided 12 ILs carrying a single SP introgression and covering 85 VOC QTLs, which were characterized at three locations. The results showed that almost half of the QTLs previously identified in the RILs maintained their effect in an IL form, reinforcing the value of these QTLs for flavor/aroma breeding in cultivated tomato.

## Highlight

Over 100 fruit volatile QTLs were identified in a RIL population derived from the red-fruited wild species *Solanum pimpinellifolium* and a fresh market tomato variety ‘Moneymaker’ and subsequently confirmed in introgression lines.

## Introduction

Tomato is cultivated worldwide, constituting one of the most important vegetable fruit crops in the world and serving as a model in the study of fruit ripening processes. In the 20th century, substantial effort was invested in tomato breeding, principally to generate high-yielding and disease-resistant varieties. However, fruit quality and consumer preferences have not generally been taken into account in breeding programs, leading to a reduction in the taste appreciated by consumers. Tomato flavor is a complex trait, composed of primary characteristics such as sugar content and acidity and, also, a large number of volatile organic compounds (VOCs) that have an important impact on flavor (Klee and Tieman, 2013).

Breeding for improved flavor by controlling the production of VOCs in the fruit has seldom been attempted for a number of reasons. First, our perception of flavor is influenced by the interaction of a relatively large number of both volatile [~16–20 or more (Buttery and Ling, 1993; Tieman *et al.*, 2012)] and non-volatile compounds (Abegaz *et al.*, 2004; Baldwin *et al.*, 2008; Tieman *et al.*, 2012), making it difficult to identify those that it would be desirable to enhance or reduce. Secondly, volatile composition is under polygenic genetic control, and the genetic basis of the mechanisms controlling their levels is largely unknown. Thirdly, volatile composition also depends on agronomic management, environment, and genetic background. Therefore, we need to identify those regions of the genome that modify volatile levels in the ripe fruit to understand this genetic control and breed for tomato flavor.

Information on the genetic control of tomato fruit volatiles is limited. Quantitative trait loci (QTLs ) for these traits have been identified in experimental populations obtained from crosses between tomato cultivars and different germplasm sources used as donor parents, such as cherry tomato (Saliba-Colombani *et al.*, 2001; Zanor *et al.*, 2009) or the distantly related, green-fruited, wild tomato species *Solanum pennellii* (Tadmor *et al.*, 2002; Tieman *et al.*, 2006*b*) and *Solanum habrochaites* (Mathieu *et al.*, 2009). In the case of these two species, wild chromosomal regions were introgressed into processing cultivars, whereas in the case of the cherry tomato, two fresh-market varieties were used as the recipient parent.

In the last decade, a number of genes involved in different metabolic pathways in the tomato fruit have been identified, shedding some light on the biosynthesis of volatile compounds. Research has led to the characterization of several genes involved in the biosynthesis of fatty acid-derived volatiles (Speirs *et al.*, 1998; Chen *et al.*, 2004; Matsui *et al.*, 2000, 2007; Shen *et al.*, 2014), apocarotenoids (Simkin *et al.*, 2004), esters (Goulet *et al.*, 2012, 2015), phenylpropanoids (Tieman *et al.*, 2010; Mageroy *et al.*, 2012), and other phenylalanine-derived volatile compounds (Tieman *et al.*, 2006*a*, 2007), and the role of conjugation in the accumulation and emission of volatiles (Louveau *et al.*, 2011; Tikunov *et al.*, 2013). However, we have only obtained partial knowledge of the metabolic pathways and the genes involved in volatile biosynthesis and regulation.

In the present work, QTLs for a large set of volatile compounds were identified in a recombinant inbred line (RIL) population derived from a cross between the fresh market variety ‘Moneymaker’ and the closely related red-fruited wild species *S. pimpinellifolium*. Most of the genetic regions containing these loci were validated in introgression lines (ILs) derived from the same parental lines, confirming their value for use in breeding programs for the fresh tomato market. Furthermore, genes and associated functions located within the QTL regions are provided; these will facilitate the identification of candidates for those genes involved in volatile biosynthesis/regulation.

## Materials and methods

### Plant material

The RIL population was originated from an interspecific cross between *S. lycopersicum* cv. ‘Moneymaker’ and *S. pimpinellifolium* accession TO-937, producing F_1_ seeds. Selfing of a single F_1_ plant generated an F_2_ segregating population from which a RIL population of 169 F_7:8_ lines was generated by the single seed descent (SSD) method (Alba *et al.*, 2009). Five plants of each RIL were grown during the winter–spring cycle in a plastic greenhouse under standard commercial growing conditions. Three biological replicates were analyzed for each line, each consisting of a mixture of 3–5 red ripe fruits obtained from trusses 2–4. This experimental design was repeated over 3 years.

An IL collection was previously generated from the same parents as the RIL population (Barrantes *et al.*, 2014). Twelve ILs covering different regions harboring selected volatile QTLs and the recurrent ‘Moneymaker’ parent were cultivated under a plastic greenhouse during the summer cycle at three locations in Spain: Alginet (Coagri co-operative), Orihuela (Miguel Hernández University), and Málaga (Institute for Mediterranean and Subtropical Horticulture ‘La Mayora,’ IHSM-UMA-CSIC). The experimental design followed a randomized block with eight blocks, with one single plant replicate per IL and six replicates of ‘Moneymaker’ in each block. Eight independent biological replicates were analyzed per genotype, each consisting of between three and five fruits from a single plant.

In addition, four *S. habrochaites* (accession LA1777) (SH) ILs, in the genetic background of the processing tomato cultivar E6203, were also analyzed. These ILs belonged to a new set of Conserved Ortholog Set II (COSII)-anchored SH ILs, which were developed to improve the previously published SH IL/BIL population (Monforte and Tanksley, 2000) (SG, unpublished data). The four SH ILs were selected to cover two genomic regions where several volatile QTLs had been mapped in the SP RILs. More specifically, two partially overlapping SH ILs—SH IL-9C and SH IL-9F—were selected for chromosome 9 to cover a region of ~50 cM (delimited by markers SNP_340a and SNP_605a); whereas, for chromosome 11, two non-overlapping SH ILs—SH IL11-B and SH IL-11C—were selected, partially covering a region of ~35 cM (defined by flanking markers: SNP_678a and SNO_700a) (see Supplementary Fig. S1 at *JXB* online). The four SH ILs had been anchored to 131 PCR-based markers: 122 COSII markers, eight cleaved amplified polymorphic sequences (CAPS), and one simple sequence repeat (SSR) marker (SG, unpublished data). The genotypic data based on these markers showed that the ILs carried a single wild introgression.

### Analysis of volatile compounds

For volatile compound analysis, red ripe tomato fruits were collected, carefully washed with water, and dried with a paper cloth to avoid external contamination from trichome secretions. A slice of pericarp (avoiding the septa) was excised from each fruit, taking care to remove all locular tissue, and immediately frozen in liquid nitrogen, ground in a cryogenic mill, and stored at –80 ºC until analysis.

Volatile compounds were captured by means of headspace solid phase microextraction (HS-SPME) and separated and detected by means of GC/MS. Samples were processed similarly to as described in Rambla *et al.* (2015). About 500 mg of frozen tomato powder were introduced into a 7 ml glass vial and incubated at 37 ºC for 10 min in a water bath. A 500 ml aliquot of an EDTA 100 mM, pH 7.5 solution and 1.1 g of CaCl_2_·2H_2_O were added, mixed gently, and sonicated for 5 min. A 1 ml aliquot of the resulting paste was transferred to a 10 ml screw cap headspace vial with a silicon/PTFE septum, and analyzed within 12 h. Volatile compound extraction and chromatography conditions were as described in Orzaez *et al.* (2009) except for the analyses of the RIL population in the first season, which were performed as described in Rambla *et al.* (2015).

For quantitation, one specific ion was selected for each compound and the corresponding peak from the extracted ion chromatogram was integrated. An admixture reference sample was prepared for each season by thoroughly mixing equal amounts of each sample. A 500 mg aliquot of the admixture was analyzed every 6–7 samples and processed as any other sample as part of the injection series. This admixture was used as a reference to normalize for temporal variation and fiber aging. Finally, the normalized results for a sample were expressed as the ratio of the abundance of each compound in that particular sample to those present in the reference admixture.

Fifty-two compounds were unequivocally identified and quantified in all the samples except in those from the RIL population in the first season, in which only 32 compounds were identified.

### Genotyping and map construction

RILs and parental lines were genotyped with the 8K SolCap Illumina Infinium SNP (single nucleotide polymorphism) tomato array (Sim *et al.*, 2012) at the INCLIVA Genotyping and Genetic Diagnosis Unit (Valencia, Spain). Monomorphic markers, as well as markers with a high number of missing values (>30%) or unknown parental genotypes were removed before further analysis. Linkage analysis and map construction were performed with JoinMap 4 mapping software (Kyazma BV, The Netherlands) (Van Ooijen, 2006) using the maximum likelihood mapping algorithm. Markers were assigned to linkage groups at a minimum LOD value of 5.0.

### QTL analysis in RILs

To facilitate computational analysis, the genetic map was condensed to 297 markers. QTL analysis was performed independently for each season with log 2-transformed data using Composite Interval Mapping with WinQTLCart software (http://statgen.ncsu.edu/qtlcart/WQTLCart.htm). The confidence interval for QTL position was established at 2–LOD units from the maximum LOD. QTLs detected in at least two seasons were retained for further analysis; QTLs only detected in a single season were discarded. Genetic and QTL maps were drawn with Mapchart 2.2 (Voorrips, 2002).

### Other statistical analysis

All data obtained from volatile analyses of the samples were log 2 transformed before the statistical analyses to achieve a normal distribution.

Correlation matrices between metabolites were constructed separately from the individual sample data from seasons 2 and 3, and from the ~1000 samples representing the sum of the two seasons. Data from season 1 were not used as they contained a lower number of compounds. Pearson correlations were calculated using SPSS 16.0 software.

Two-way ANOVA was used to determine the effects of genotype, environment, and genotype×environment interaction on the levels of each of the volatiles being analyzed. For the RIL population, we used data from seasons 2 and 3. For the IL population, data from the three different locations were used. ANOVA was conducted using Statgraphics Centurion XVI.II 64 bit software.

To evaluate the statistical significance of the differences between the average of the volatiles in each of the ILs and those in the recipient parent ‘Moneymaker’, Dunnett’s test (*P*<0.05) was used. We considered that a QTL previously identified in the RILs was confirmed in the respective IL when the level of that particular compound was significantly different from the control parent from at least one of the three locations evaluated. Our criterion for considering a new volatile QTL was that it was observed in fruits of an IL from at least two of the three locations. Dunnett’s test was conducted with JMP 12 software.

### Hierarchical cluster analysis and cluster similarity

Hierarchical cluster analysis (HCA) based on correlations (Eisen *et al.*, 1998) was performed for the combined data set obtained from the RILs of seasons 2 and 3, and also over each data set individually. To assess the similarity between clusterings with statistical significance, the metric proposed by Fowlkes and Mallows (1983) was used, and random data sets were generated by permutation (bootstrapping). Data were analyzed with Matlab (MathWorks).

## Results and discussion

In the present study, we used an *S. lycopersicum*×*S. pimpinellifolium* RIL population which had previously been employed for the localization of QTLs involved in pest resistance (Salinas *et al.*, 2013) and other fruit quality components (Capel *et al.*, 2015). Line TO-937 was derived from an *S. pimpinellifolium* accession originally collected in Lambayeque, Peru (Alba *et al.*, 2009), which produces the species’ typical small, red fruits containing higher levels of many flavor compounds and a number of healthy metabolites such as sugars, organic acids, vitamin C, or carotenoids (Capel *et al.*, 2015). ‘Moneymaker’ is a medium-size fruit cultivar for the fresh market with an indeterminate growth habit that is commonly used in genetic, physiological, phytopathological, and developmental studies. The two species are close relatives and, in contrast to wild green-fruited tomato species that have been used for genetic analyses of fruit quality traits, they share common characteristics for fruit ripening, metabolite profiling, etc. Additionally, they present no major cross-incompatibility that might cause substantial segregation distortions or the elimination of introgressed genomic regions in the mapping populations.

### The volatile network

**Fig. 1.**
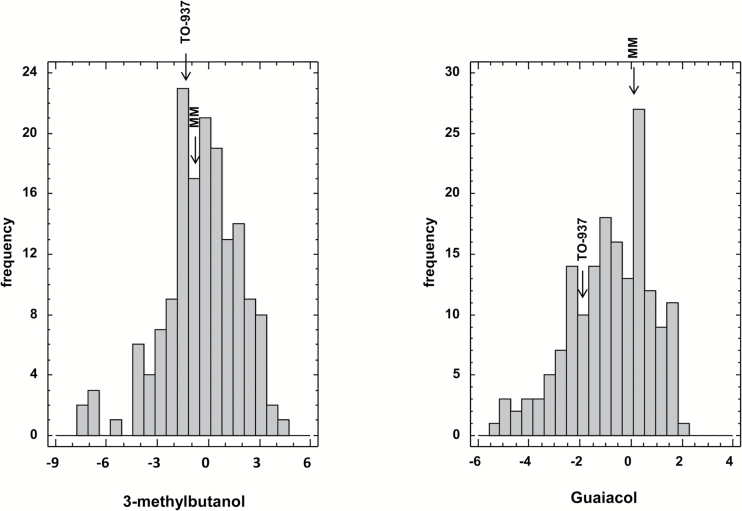
Histogram of the distribution of 3-methylbutanol and guaiacol in the RIL population. The positions of the parentals *S*. *lycopersicum* cv. ‘Moneymaker’ (MM) and *S. pimpinellifolium* accession TO-937 are indicated with an arrow.

Fifty-two different volatile compounds were unequivocally identified in the ripe fruit samples obtained from the RIL population. A continuous variation was observed, with transgressive segregation in both directions for all the compounds (Supplementary Fig. S2), showing a large degree of variation for most of the volatiles, with up to 15 of them displaying a >100-fold difference in levels between the RILs with extreme values. Compounds displaying the highest degree of variation included phenylpropanoids such as guaiacol, eugenol, or methyl salicylate, other phenylalanine derivatives such as 1-nitro-2-phenylethane, benzylnitrile, or 2-phenylethanol, and branched-chain amino acid-related compounds such as 2-isobutylthiazole, 3-methylbutanol, 2-methylbutanol, 3-methylbutanal, 3-methylbutanoic acid, or 3-methylbutanenitrile. It must be noted that broad variability was detected not only for volatiles that were differentially accumulated between the parents of the population (i.e. guaiacol) but also for those that showed very similar levels in both of them (i.e. 3-methylbutanol) as illustrated in Fig. 1. On the other hand, a minority of volatiles showed a relatively low degree of variation, such as fatty acid derivatives octanal, nonanal, and decanal, with a <5-fold range between the RILs with extreme values (Supplementary Table S1).

This indicates that by combining the genomes of the two parents in the RIL population, we generated new unpredictable variability in fruit volatile content.

**Fig. 2.**
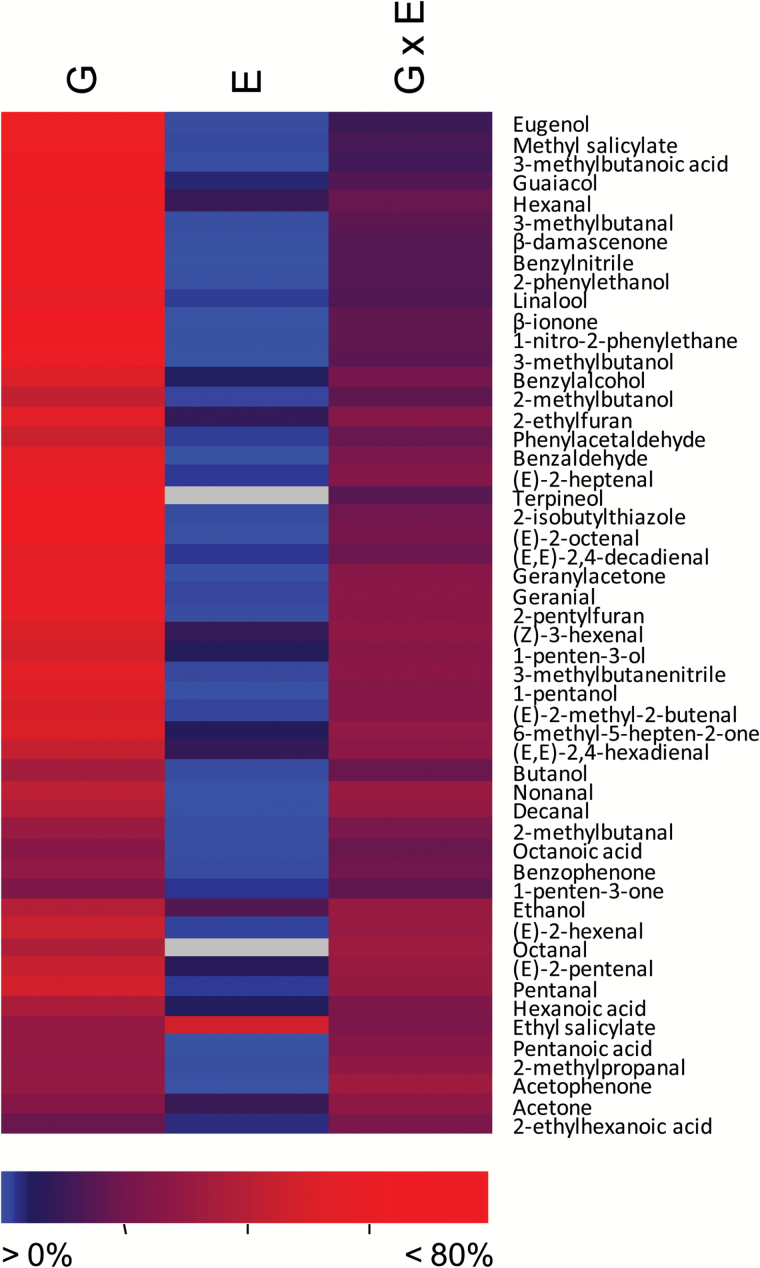
Heat map showing the effect of genotype (G), environment (E), and the genotype×environment (G×E) interaction for each volatile in the RIL population. Data correspond to the percentage of variability explained, according to the scale below. High values (up to 80%) are indicated in red and low values in blue. The exact values are detailed in Supplementary Table S2.

Statistical analysis of the volatile levels revealed that the genotype had a highly significant effect on all the volatiles profiled and was the principal factor explaining the variability found (Supplementary Table S2). The genotypic effect was particularly high in the case of some apocarotenoid volatiles such as β-ionone and β-damascenone, several branched-chain amino acid-related volatiles, phenylalanine derivatives, and particularly phenylpropanoids such as eugenol and methyl salicylate, for which the genotype explained >80% of the total variance. Interestingly, most of the traits with higher heritability were also among those with a larger degree of variation between the RILs with extreme values. For compounds with low heritability, genotypically explained variability scored ~20%, and included volatiles from different metabolic pathways such as fatty acid derivatives 2-ethylhexanoic acid, and 1-penten-3-one. It should be noted that interaction between genotype and environment was also highly significant for almost all the volatiles, and accounted for ~15–30% of total variability for most of the compounds. The effect of the environment was significant but remarkably low for most of the volatiles, with just a few exceptions, such as in the case of ethyl salicylate (Fig. 2; Supplementary Table S2). Therefore, these results suggest that the environment—at least under our experimental conditions—did not have a general effect on variability in volatile biosynthesis, which was modulated mostly by the particular combination of genes in each RIL.

### Correlation between metabolites

**Fig. 3.**
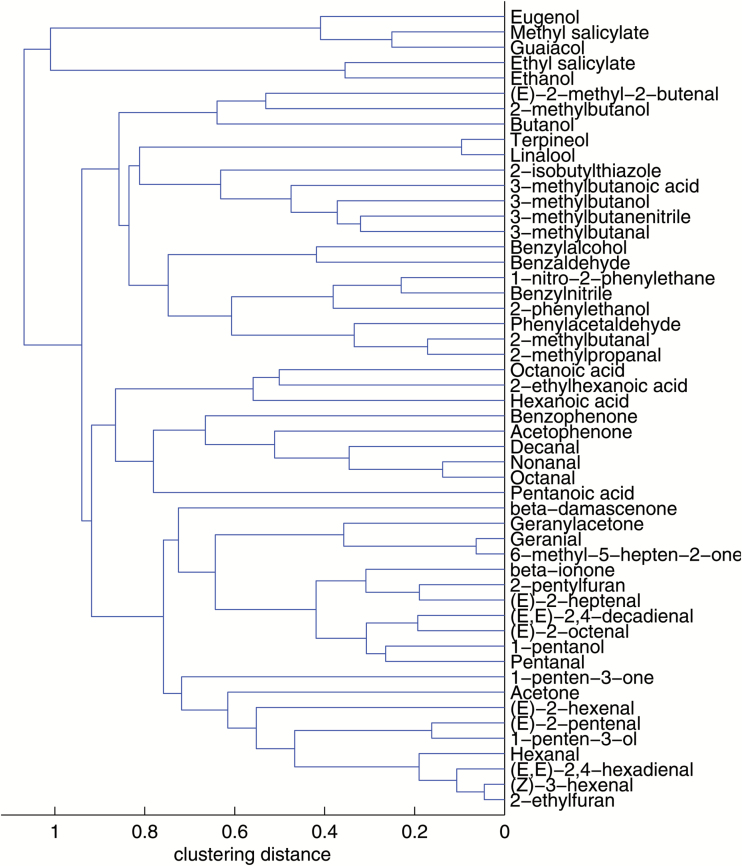
Hierarchical cluster of volatiles in the RIL population in seasons 2 and 3. (This figure is available in colour at *JXB* online.)

A correlation matrix was constructed from the results of the analysis of volatiles for each of seasons 2 and 3 separately, and for the two seasons together, to determine the relationship between the 52 volatile compounds identified in the ripe fruit (Supplementary Table S3). Statistical analyses were performed on data from >1000 biological samples corresponding to 169 independent RILs derived from the cross between the cultivated tomato and the red-fruited wild relative *S. pimpinellifolium*, each line harboring about a half of the randomly inherited genome of each parental line. Therefore, the correlation matrix obtained from this set of data can be considered as a reliable, largely unbiased description of the relationship between volatile compounds in tomato. Assuming that *S. pimpinellifolium* is the closest wild relative of tomato, we could consider that each RIL represents different alterations of the tomato volatile network, but these would not be as drastic as those occurring when combining more distant relatives. On the whole, the correlation results for both seasons matched, as revealed by the independent HCAs of correlations from the data set of one season or the other. Similarity between the hierarchical trees of seasons 2 and 3 was assessed following the metric proposed by Fowlkes and Mallows (1983), which consists of cutting the two hierarchical trees and then counting the number of matching entries in a number of clusters in each tree. We obtained a clustering comparison metric of 0.45 for 10 clusters (note that this metric ranges from 0 to 1; the higher the metric, the greater the degree of similarity). To assess the corresponding statistical significance, we kept the data set for season 3 and generated 1000 random data sets by permuting the values in the data set for season 2 (bootstrapping). By comparing each of the random data sets against the data set for season 3, we obtained a null distribution of the clustering comparison metric, with mean 0.13. This gave a *P*-value of 0 for the value of 0.45, indicating that trees derived from both seasons are statistically similar. Because this clustering comparison metric depends on the number of clusters selected upon cutting, we decided to reassess the statistical significance for five clusters. The clustering comparison metric between seasons was now 0.53, and the mean of the recalculated null distribution 0.27. This also resulted in a *P*-value of 0, reinforcing the similarity of the two trees (Supplementary Fig. S3). HCA was also performed using the results of the volatile levels in each of the samples for both seasons together, producing clusters for volatile compounds, thus facilitating their classification according to their respective correlations (Fig. 3). HCA with the average results of the RILs was also performed in order to facilitate their classification according to their volatile profile (Supplementary Fig. S4).

A number of factors may have caused the parallel levels of different compounds in the RIL population, such as those shown in both the correlation matrices and the HCA. Two or more genes may possibly control the metabolism of compounds that are physically very closely linked one to another, so that any recombination between them would be rare and the lines with a particular allele for one of the genes may have also inherited the same allele for the other gene(s). Nevertheless, the most appropriate explanation in most cases is that significant correlations between volatiles are due to some common process in their biosynthesis, such as sharing the same precursor, or an enzyme in the pathway, or the existence of an independent process favoring both pathways (i.e. cell wall degradation which would facilitate the availability of different substrates). Consequently, cluster analysis had previously been used to propose an assignment of volatile compounds to metabolic pathways (Tikunov *et al.*, 2005; Mathieu *et al.*, 2009), constituting a valuable tool to explain the biosynthesis of those compounds for which a metabolic pathway has not been clearly established. Thus, the most relevant results obtained from both hierarchical cluster and correlation analyses will be discussed hereafter in these terms.

On the basis of its chemical structure, geranial is a monoterpenic aldehyde and could be expected to cluster with the monoterpenoids linalool and terpineol. However, geranial is located in the apocarotenoid cluster, as previously reported (Tikunov *et al.*, 2005), and has a very strong positive correlation with 6-methyl-5-hepten-2-one (*r*=0.94), thus suggesting a common substrate in the biosynthesis of both compounds, probably the linear carotenoid lycopene and, perhaps, also a common carotenoid cleavage dioxygenase enzyme with both 5,6 and 7,8 cleavage activities.

In the case of β-damascenone, it has been suggested that β-carotene could be its precursor on the grounds that this compound clusters with β-ionone in fruit volatile data sets obtained from ILs derived from other wild tomato species (Mathieu *et al.*, 2009). However, in our RIL population, levels of β-damascenone did not correlate strongly with β-ionone. On the contrary, it was located in another cluster, with linear apocarotenoids 6-methyl-5-hepten-2-one, geranylacetone. and geranial, with which it even showed a slightly higher correlation (*r*=0.30–0.33) than with β-ionone (*r*=0.26). Therefore, our results reinforce the hypothesis that we might consider precursors other than β-carotene, such as neoxanthin, proposed elsewhere (Skouroumounis *et al.*, 1993). Alternatively, the low correlation observed between β-damascenone and all the other apocarotenoids (*r*<0.33) suggests a different mechanism of synthesis, as reviewed in Sefton *et al.* (2011) proposing a chemical (non-enzymatic) acid-catalyzed hydrolysis of different plant-derived apocarotenoids as the origin of at least part of the β-damascenone occurring in a number of fruit and foodstuffs.

It has been suggested that the disruption or alteration in permeability of the thylakoid membranes during the chloroplast to chromoplast transition would facilitate the access of both lipoxygenases and carotenoid cleavage dioxygenases to their substrates, non-esterified fatty acids and carotenoids, respectively (Mathieu *et al.*, 2009). Our data may support this hypothesis, considering that β-ionone showed the strongest correlations with fatty acid derivatives such as (*E*)-2-heptenal and hexanal (*r*=0.74 and 0.71, respectively). β-Ionone also shows a notably stronger correlation with geranylacetone than with any other apocarotenoid, which is consistent with both compounds resulting from reactions catalyzed by the same enzyme, as seems to be the case for LeCCD1 enzymes with 9,10 cleavage specificity acting on both cyclic and non-cyclic carotenoid substrates (Simkin *et al.*, 2004).

Two of the volatile compounds located in the fatty acid derivative cluster are 2-ethylfuran and 2-pentylfuran. A metabolic pathway has not yet been elucidated for these compounds but cluster analyses conducted on independent materials of tomato (Tikunov *et al.*, 2005; own unpublished data) and strawberry (Zorrilla-Fontanesi *et al.*, 2012) have repeatedly suggested that both compounds could be derived from fatty acids. This hypothesis is strengthened by the fact that the C_9_ volatile 2-pentylfuran clustered with C_8_–C_10_ fatty acid derivatives in our RIL population, while 2-ethylfuran (a C_6_ compound) clustered with double-bonded C_6_ fatty acid derivatives. The latter volatile showed a remarkably strong correlation with (*Z*)-3-hexenal (*r*=0.95), suggesting that (*Z*)-3-hexenal might be postulated as a plausible precursor of 2-ethylfuran.

### Identification of volatile QTLs

The 8K SolCap Illumina Infinium SNP tomato array (Sim *et al.*, 2012) containing 7720 SNPs was used to genotype the *S. lycopersicum*×*S. pimpinellifolium* RIL population. Of the 7720 markers, 2941 (38%) were monomorphic in the RILs and 538 (7%) could not be reliably genotyped. Therefore, the remaining 4241 markers (around 55% of the SNPs included in the array) were used to generate a genetic linkage map. The markers in the resulting map were assigned to 1704 unique map locations at an average genetic distance of 1.0 cM and a maximum of 8 cM. For QTL analysis, a skeleton map was developed consisting of 297 markers evenly distributed along the tomato genome.

A total of 102 QTLs distributed along all the 12 tomato chromosomes were identified for 39 of the 52 volatile compounds (VOCs) identified in the samples obtained from the 169 RILs (Fig. 4; Supplementary Table S4). Chromosome 1 contained a particularly high number of VOC QTLs—a total of 31 for compounds synthesized via different metabolic pathways. The alleles inherited from *S. pimpinellifolium* (SP) produced an increase in the levels of volatiles in 42 loci, whereas they produced reduced levels in the remaining 60. Regarding the metabolic pathways, the production of phenolic volatile compounds tended to be increased by *S. lycopersicum* (SL) alleles, while levels of apocarotenoid volatiles were more likely to be increased by the SP alleles. For the other metabolic pathways, no clear tendency was observed. These results point to *S. pimpinellifolium* as a species with a potential in terms of breeding for cultivated tomato flavor, as apocarotenoid volatiles have been highlighted as important contributors to liking tomato (Vogel *et al.*, 2010; Tieman *et al.*, 2012).

**Fig. 4.**
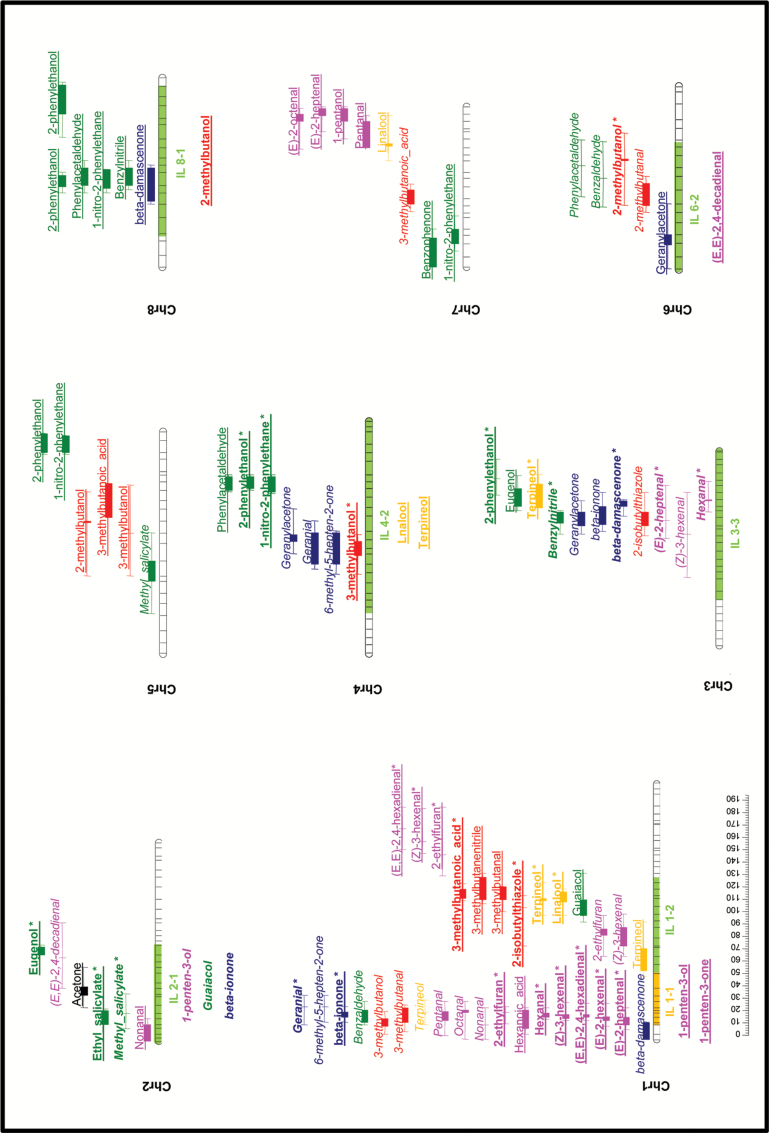
Volatile QTL map obtained from the RIL and IL populations. The metabolic pathways of volatiles are distinguished by using different colors: pink, fatty acid derivatives; green, phenolic compounds; red, branched-chain amino acid-related compounds; orange, terpenoids; blue, apocarotenoids. Loci at which SP alleles induced higher levels of volatiles are marked in italics; those in which SP alleles induced lower levels are marked with plain underlined text. QTLs identified in the RIL population are shown on the right side of each chromosome. The interval corresponds to extreme values of LOD–2 and LOD+2 in the different seasons; the central solid area corresponds to the interval overlapping in the different seasons. QTLs confirmed in the ILs are highlighted in bold and marked with an asterisk. New QTLs identified in the ILs are shown to the left of each chromosome.

**Fig. 4.**
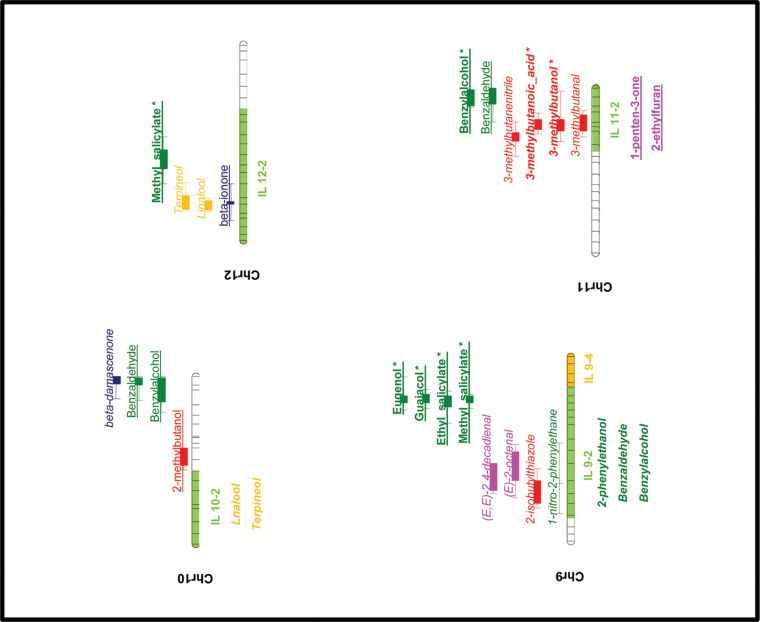
Continued.

#### Fatty acid derivatives

QTLs modifying the levels of volatiles derived from fatty acids were identified in five different chromosomes. Note that a region located at the top of chromosome 1 strongly influenced the levels of 10 different volatile compounds. The SP allele decreased levels of six C_6_ volatiles plus C_7_ (*E*)-2-heptenal, while the levels of the non-C_6_ aliphatic aldehydes pentanal, octanal, and nonanal were increased, indicating the opposite effect of this region on the production of these two groups of compounds, which actually clustered independently and, in most cases (with the exception of pentanal), showed significant negative correlations (Supplementary Table S3). The metabolic pathway of many of these compounds is known, and some key genes involved in their biosynthesis in tomato fruit have been described. Free fatty acids are first oxidized by means of lipoxygenase (LOX) enzymes, and the resulting molecule is subsequently broken down by means of hydroperoxide lyases (HPLs) to produce one non-polar volatile metabolite and one polar non-volatile compound (Rambla *et al.*, 2014). The lipoxygenase gene *TomloxC* (Chen *et al.*, 2004) has been mapped in this region of chromosome 1 and is a reasonable candidate gene for this major QTL, which in some seasons accounts for >50% of the total variability of some compounds. The HPL gene *LeHPL* (Matsui *et al.*, 2000) has been mapped at the bottom of the same chromosome in a region where three C_6_ volatile QTLs were identified. Therefore, this gene may also have an effect on the levels of fatty acid-derived volatiles, although to a much lower extent than *TomloxC*, as revealed by the *R* ^2^ value (Supplementary Table S4).

#### Phenolic compounds

Over 30 volatile QTLs were identified for phenolic compounds spread across all the tomato chromosomes. Major QTLs accounting for over one-third of the total variability were identified for guaiacol, eugenol, methyl salicylate, and, to a lesser extent, ethyl salicylate at the bottom of chromosome 9 (Supplementary Table S4), in the same region mapped by the *NSGT1* glycosyltransferase gene (Tikunov *et al.*, 2013). Many minor QTLs for these compounds were identified at other locations, including one for guaiacol in the same region of chromosome 1 where *SlUGT5*, a gene involved in the glycosylation of many phenylpropanoids, is located (Louveau *et al.*, 2011). Additionally, QTLs for phenylalanine-derived volatiles benzylnitrile, 1-nitro-2-phenylethane, phenylacetaldehyde, and 2-phenylethanol mapped in the same region of chromosome 8 as *LeAADC* genes, which have been reported to be involved in their biosynthesis (Tieman *et al.*, 2006*a*).

#### Branched-chain amino acid-related compounds

QTLs for several branched-chain volatiles with chemical structures resembling those of the amino acids leucine or isoleucine have been identified on chromosomes 1, 3, 4, 5, 6, 7, 9, 10, and 11. The biosynthetic pathway of these volatiles has not yet been established in plants, but it has been suggested that these branched-chain amino acids might be their direct precursors (Mathieu *et al.*, 2009). Nevertheless, recent evidence indicates that these volatiles are more likely to be derived from their respective keto acids rather than from the amino acids (Kochevenko *et al.*, 2012). Although QTLs controlling branched-chain volatiles were identified in most of the chromosomes, those for structurally leucine-related volatile compounds (3-methylbutanal, 3-methylbutanol, 3-methylbutanenitrile, 3-methylbutanoic acid, and 2-isobutylthiazole) only mapped in the same position as those for isoleucine-related volatile compounds [2-methylbutanal, 2-methylbutanol, and (*E*)-2-methyl-2-butenal] on chromosome 5. This indicates that most of the regulation of each group of compounds is independent of group, which is confirmed by the significant, but low, positive correlation observed between the groups.

The only sulfur compound identified in our samples was the branched-chain volatile 2-isobutylthiazole. QTLs for this compound were identified on chromosomes 1, 3, and 9. The 2-isobutylthiazole QTL detected on chromosome 1 co-localized with QTLs for 3-methylbutanol, 3-methylbutanoic acid, and 3-methylbutanenitrile, and the SP alleles reduced the levels of all of them. The other two QTLs identified for 2-isobutylthiazole did not co-localize with loci controlling other volatiles in the same pathway, which points to those regions being likely to harbor the genes responsible for the addition of the sulfur moiety.

#### Terpenoids

Very little is known about terpenoid biosynthesis in the tomato fruit, and only two of the compounds in this pathway were identified in the samples analyzed. We have detected three and five QTLs that regulate the levels of the terpenic alcohols linalool and terpineol, respectively. These QTLs map on chromosomes 1, 3, 7, and 12. Both compounds provide floral notes, although they are not thought to affect tomato flavor (Buttery and Ling, 1993). SP alleles are associated with reduced levels in all the QTLs identified except for those mapping on chromosome 12. Therefore, these chromosome 12 QTLs would be the target of choice for breeding enhanced levels of these potentially desirable volatiles. The chromosomal regions where a QTL for these compounds maps may contain terpene synthases or other genes involved in their biosynthesis, which could be used in the search for candidate genes for their biosynthetic pathway.

#### Apocarotenoids

Fourteen QTLs for a set of volatiles derived from carotenoids were identified on chromosomes 1, 3, 4, 6, 8, 10, and 12. A cluster of QTLs controlling the levels of linear apocarotenoids geranylacetone, geranial, and 6-methyl-5-hepten-2-one was identified on chromosome 4. Another cluster of QTLs was identified on chromosome 3 with an effect on both linear and cyclic C_13_ apocarotenoid volatiles. In both cases, the SP alleles induced increased levels. Interestingly, chromosome 1 harbors a region containing QTLs with the opposite effect on linear compounds 6-methyl-5-hepten-2-one (C_8_) and geranial (C_10_) (alleles from SP increasing their levels) and the cyclic C_13_ compound β-ionone (decreased by SP alleles). It should be noted that carotenoid cleavage dioxygenase 1 genes (*LeCCD1A* and *LeCCD1B*), which have been described as participating in the biosynthesis of C_13_ apocarotenoids (Simkin *et al.*, 2004), also map in this region of chromosome 1.

Up to nine QTLs for the carotenoids lycopene and β-carotene had previously been identified in the same RIL population (Capel *et al.*, 2015). However, apocarotenoid volatile QTLs in general tended not to co-localize with carotenoid QTLs, with the exception of the QTLs for 6-methyl-5-hepten-2-one, geranial, and geranylacetone on chromosome 4 which co-localized with QTLs for both lycopene (the plausible precursor of them all) and β-carotene. This indicates that the main determinants of the production of apocarotenoid volatiles are factors other than substrate accumulation. Therefore, all the other QTLs identified could be due to as yet unknown genes located in these regions implied either directly in the final steps of volatile biosynthesis, or in the accumulation of possible precursors other than the already described lycopene and β-carotene, or in other processes facilitating the interaction between substrates and biosynthetic enzymes.

### Introgression and validation of the volatile QTLs in a fresh market tomato genetic background

Twelve SP ILs previously generated from the same cross (Barrantes *et al.*, 2014) carrying single SP introgressions covering 85 of the QTLs identified in the SP RIL population (Fig. 4) were grown at three different locations, and their fruit volatile composition was determined to validate the effect of the QTLs selected.

In contrast to the RIL population, most of the variability observed for the volatile levels in the fruit of these ILs was due to the environment and the genotype, and significant genotype×environment interaction was only observed for a few compounds (Supplementary Table S5). This could be attributed to the high genetic background isogenicity of the ILs, with an average genetic difference between ILs slightly over 10%, whereas between RILs it was ~50%. This reduction of genotypic variability between ILs may also decrease the power of genotype×environment interaction detection.

Thirty-four of the 85 QTLs were confirmed in the 12 ILs, maintaining the direction of the effect on the volatile levels after a single introgression of the *S. pimpinellifolium* in the fresh market tomato variety ‘Moneymaker’ (Fig. 4). This can be considered indicative of the potential of the QTLs identified for their use in breeding tomato flavor. Nevertheless, the introgression of a genetic region has been described as having a different effect on the volatile profile depending on the genetic background where it is introgressed. Therefore, it seems plausible that the introgression of the same regions of the chromosomes in a different tomato variety might alter different sets of volatiles, as previously observed when introgressing selected regions of cherry tomato in different elite beef tomato varieties (Zanor *et al.*, 2009).

**Fig. 5.**
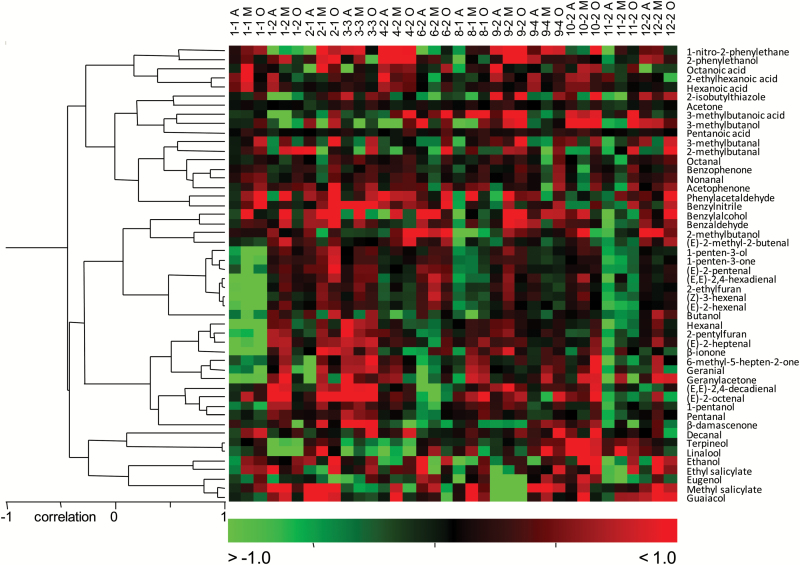
Heat map showing volatile levels in the ILs. Values correspond to the log_2_ of the ratio of the levels of each volatile in the ILs in relation to those in *S. lycopersicum* cv. ‘Moneymaker’, cultivated at each location. Higher levels are shown in red; lower levels in green; identical levels in black. The letter after the IL descriptor indicates where it was cultivated: ‘A’, Alginet; ‘M’, Malaga; ‘O’, Orihuela.

Additionally, 16 new QTLs for volatile compounds corresponding to different metabolic pathways were identified on chromosomes 1, 2, 4, 6, 8, 9, 10, and 11, represented by eight SP ILs (Fig. 4). Some of these new QTLs corresponded to volatiles synthesized in the same metabolic pathway as a VOC QTL previously detected in the RIL population, as in the case of fatty acid derivatives 1-penten-3-ol and 1-penten-3-one in chromosome IL 1-1, which also contained QTLs for another six metabolically related volatiles. This suggests that a gene located in that chromosomal region has a general effect on the whole metabolic pathway, enhancing the accumulation of many different volatiles in the pathway. However, some new QTLs were detected in the ILs unrelated to those mapped in the RIL population. This is the case for the terpenoids linalool and terpineol (in IL 4-2 and IL 10-2), apocarotenoid β-ionone (IL 2-1), branched-chain volatile 2-methylbutanol (IL 8-1), and fatty acid derivatives (*E*,*E*)-2,4-decadienal (IL 6-2) and 1-penten-3-one and 2-ethylfuran (IL 11-2). Additionally, we found significant IL effects for some individual compounds at a single location, but other volatiles belonging to the same metabolic pathway changed significantly in the same ILs at other locations. Although these loci were not included in the QTL map presented here, we consider that they correspond to minor genes with an effect on early stages of the metabolic pathway, therefore affecting the levels of a number of compounds downstream. Complete data on volatile levels in the ILs are shown in Fig. 5 and Supplementary Table S6.

### *Similarities with QTLs identified in selected ILs developed from* S. habrochaites

Two partially overlapping *S. habrochaites* LA1777 (SH) ILs were selected from the new set of COSII-anchored SH ILs for chromosome 9: ILSH-9C and ILSH-9F (Supplementary Fig. S1). Analyses of their fruit volatile profiles revealed that both produced dramatically reduced levels of guaiacol, methyl salicylate, and eugenol. This is in agreement with the QTLs previously observed in both RIL and IL populations derived from *S. pimpinellifolium*, and also in near isogenic lines derived from a cherry tomato donor (Zanor *et al.*, 2009). Additionally, ILSH-9F showed significantly reduced levels of (*E*,*E*)-2,4-decadienal and 1-nitro-2-phenylethane, for which QTLs were detected in the SP RILs but not in the SP ILs. Finally, QTLs for 2-phenylethanol and benzaldehyde, which were detected in IL 9-2 from ‘Moneymaker’ but had not been identified in the RILs, were also confirmed in ILSH-9F. Surprisingly, no QTLs for volatile compounds had been identified on chromosome 9 when the previously published SH IL/BIL population was analyzed (Mathieu *et al.*, 2009). This might be partially due to the different SH ILs used in the previous study to cover this chromosome 9 region.

Another two non-overlapping ILs—ILSH-11B and ILSH-11C—were selected for chromosome 11 from the new set of COSII-anchored SH ILs (Supplementary Fig. S1). All of the four QTLs for branched-chain volatiles 3-methylbutanol, 3-methylbutanal, 3-methylbutanoic acid, and 3-methylbutanenitrile previously identified in the *S. pimpinellifolium* RILs were also detected in ILSH-11B. Of these QTLs, only those for 3-methylbutanol and 3-methylbutanoic acid were detected in the SP ILs. Interestingly, QTLs for these compounds were not detected in the previously published SH IL population (Mathieu *et al.*, 2009), but most of them had been described in an IL population developed from *S. pennellii* (Tieman *et al.*, 2006). ILSH-11C showed significantly higher levels of guaiacol, as described in the earlier SH IL population (Mathieu *et al.*, 2009). However, no QTLs were detected for benzaldehyde and benzyl alcohol, although previous ILs from both SH (Mathieu *et al.*, 2009) and our SP population facilitated the identification of loci for these compounds.

### Volatile QTLs identified in tomato show a low degree of overlapping

QTLs for volatile compounds in tomato fruit have been previously identified in a RIL population developed from an intraspecific cross between *S. lycopersicum* var. *cerasiforme* inbred line ‘Cervil’ and the *S. lycopersicum* inbred line ‘Levovil’ (Saliba-Colombani *et al.*, 2001) or in selected ILs generated from the same cross in two inbred line genetic backgrounds (Zanor *et al.*, 2009), in a particular IL (Tadmor *et al.*, 2002), or an IL population derived from *S. pennellii* in the M82 processing tomato background (Tieman *et al.*, 2006*b*), and also in an IL population generated from *S. habrochaites* in the *S. lycopersicum* cv. E6203 background (Mathieu *et al.*, 2009). It should be noted that there is a very low degree of overlapping of the QTLs described. Only 26 of the 102 VOC QTLs detected in the SP RIL population had been reported in any of the previous studies, whereas the other 76 QTLs are described here for the first time (Table 1). Moreover, only two of those 26, the QTLs for guaiacol on chromosome 1 (probably corresponding to the *SlUGT1* gene) and for phenylacetaldehyde on chromosome 8 (probably due to *LeAADC* genes), had been reported in more than one previous experiment. This lack of consistent results could be explained in part by the differences in the environmental conditions in each of the experiments. Although environmental conditions explained on average only 3.4% of total variability in this experiment, environmental effects accounted for up to 47% in individual compounds, and this variability would probably be higher when comparing fruits grown in different geographical locations. Additionally, different analytical methods were employed to determine volatile compounds. The precise method used for volatile determination, including both sample preparation and the extraction technique, has a major effect on the compounds that can be detected, as recently stated (Rambla *et al.*, 2015). Therefore, a perfect match in the QTLs identified by different research groups should not be expected even if obtained from the same biological material. Nevertheless, even when some discrepancy exists between the results available, co-localization of different QTLs from metabolically related compounds can be used as a clue to their probable association with the same gene. For example, we identified QTLs for four phenylalanine-derived volatiles: 1-nitro-2-phenylethane, phenylacetaldehyde, 2-phenylethanol, and benzylnitrile, in the same region of chromosome 8 where Saliba-Colombani *et al.* (2001) only found a QTL for phenylacetaldehyde, and Tadmor *et al.* (2002) and Tieman *et al.* (2006*b*) described QTLs for phenylacetaldehyde and 2-phenylethanol. In our opinion, the most plausible explanation for these results is that there is a single gene in that region influencing the levels of at least all these four compounds, and the ability to identify a lower or a higher number of volatile QTLs depends largely on the particular analytical method employed by each research group and the environmental conditions in each particular experiment.

**Table 1.** Volatile QTLs with possible overlap with previously identified QTLs

	Saliba-Colombani *et al.* (2001) Cherry tomato	Zanor *et al.* (2009) Cherry tomato	Tadmor *et al.* (2002) *S. pennellii*	Tieman *et al.* (2006*b*) S*. pennellii*	Mathieu *et al*, (2009) *S. habrochaites*
Chr 1		Guaiacol		Guaiacol	Guaiacol
					(*Z*)-3-Hexenal
					3-Methylbutanol
	Pentanal				
	Hexanal				
		Terpineol			
Chr 2		Eugenol			
Chr 4					3-Methylbutanol
	Phenylacetaldehyde	Phenylacetaldehyde			
		2-Phenylethanol			
Chr 6				2-Methylbutanol	
				Benzaldehyde	
Chr 8	Phenylacetaldehyde		Phenylacetaldehyde	Phenylacetaldehyde	
			2-Phenylethanol	2-Phenylethanol	
Chr 9	Guaiacol	Guaiacol			
	Eugenol	Eugenol			
		Methyl salicylate			
Chr 10				2-Methylbutanol	
					Benzaldehyde
					Benzyl alcohol
Chr 11				3-Methylbutanal	
				3-Methylbutanol	
				3-Methylbutanenitrile	
					Benzaldehyde
					Benzyl alcohol

Nevertheless, even after considering that part of the lack of redundancy is due to environmental or methodological factors, a substantial part of it can still be claimed to respond to specific genetic variation, as no QTLs for volatiles in the same metabolic pathway had been reported earlier for most of the genetic regions where volatile QTLs have been described here. This is in consonance with the results derived from the comparison between the QTLs reported in ILs derived from *S. pennellii* (Tieman *et al.*, 2006*b*) and *S. habrochaites* (Mathieu *et al.*, 2009), in which only a minor part of them (10 out of 60) were overlapping, despite the same analytical method having been employed in both studies. Taken all together, this suggests that there is ample genetic variability in wild species of the tomato clade readily available for breeding improved/new fruit flavor and aroma by modulating the levels of volatile compounds.

## Supplementary data

Supplementary data are available at *JXB* online.

Fig. S1. Schematic overview of tomato chromosomes 9 and 11 showing the map position of the four *Solanum habrochaites* LA1777 (SH) ILs analyzed.

Fig. S2. Histogram of the distribution of each volatile compound in the RIL population in seasons 1, 2, and 3.

Fig. S3. Hierarchical clusters of volatiles in seasons 2 and 3 independently and an evaluation of the degree of similarity.

Fig. S4. Hierarchical cluster of the RILs according to their volatile profile in seasons 2 and 3 together.

Table S1. Levels of volatile compounds in the RILs with extreme values and in their parental lines..

Table S2. Effect of genotype, environment, and genotype×environment interactions on levels of the volatile compounds in the RILs and statistical significance, as obtained using ANOVA.

Table S3. Correlation between volatiles and significance.

Table S4. Volatile QTL map obtained from the RIL population.

Table S5. Effect of genotype, environment, and genotype×environment interactions on the levels of the volatile compounds in the ILs and statistical significance, as obtained using ANOVA.

Table S6. Levels of volatile compounds in the ILs.

## Supplementary Material

supplementary_figures_S1_S4

supplementary_table_S1

supplementary_table_S2

supplementary_table_S3

supplementary_table_S4

supplementary_table_S5

supplementary_table_S6
