# Supplementary material for: Identification, introgression, and validation of fruit volatile QTLs from a red-fruited wild tomato species
Source: J Exp Bot. 2016 Dec 31;68(3):429–42. doi: 10.1093/jxb/erw455 (PMC5444475; doi:10.1093/jxb/erw455)
Supplement: supplementary_figures_S1_S4 [file erw455_suppl_supplementary_figures_S1_S4.pdf]

Identification, introgression and validation of fruit volatile QTLs from a red-fruited wild tomato species
Running title: Fruit volatile QTLs and their introgression in tomato

José L. Rambla[1], Aurora Medina[1], Asun Fernández-del-Carmen[1], Walter Barrantes[1†], Silvana Grandillo[2], Maria Cammareri[2], Gloria López-Casado[4], Guillermo Rodrigo[1], Arancha Alonso[5], Santiago García-Martínez[5], Jaime Primo[3], Juan J. Ruiz[5], Rafael Fernández-Muñoz[4], Antonio J. Monforte[1], Antonio Granell[1*]

Supplementary figures

Figure S1. Schematic overview of tomato chromosomes 9 and 11 showing the map position of the four *Solanum habrochaites* LA1777 (SH) ILs analyzed. Map distances in centimorgan (cM) are indicated on the right side of each chromosome (based on the EXPEN 2000 mapping population (http://solgenomics.net/)). The total genetic length is given at the bottom of each chromosome. Marker names are indicated on the left side of each chromosome. COSII markers are identified by the name of the most similar Arabidopsis gene. The SH ILs are indicated with green bars on the right of each chromosome. The borders of the bars are arbitrarily drawn midway between markers positive and negative for the introgressed SH segment.

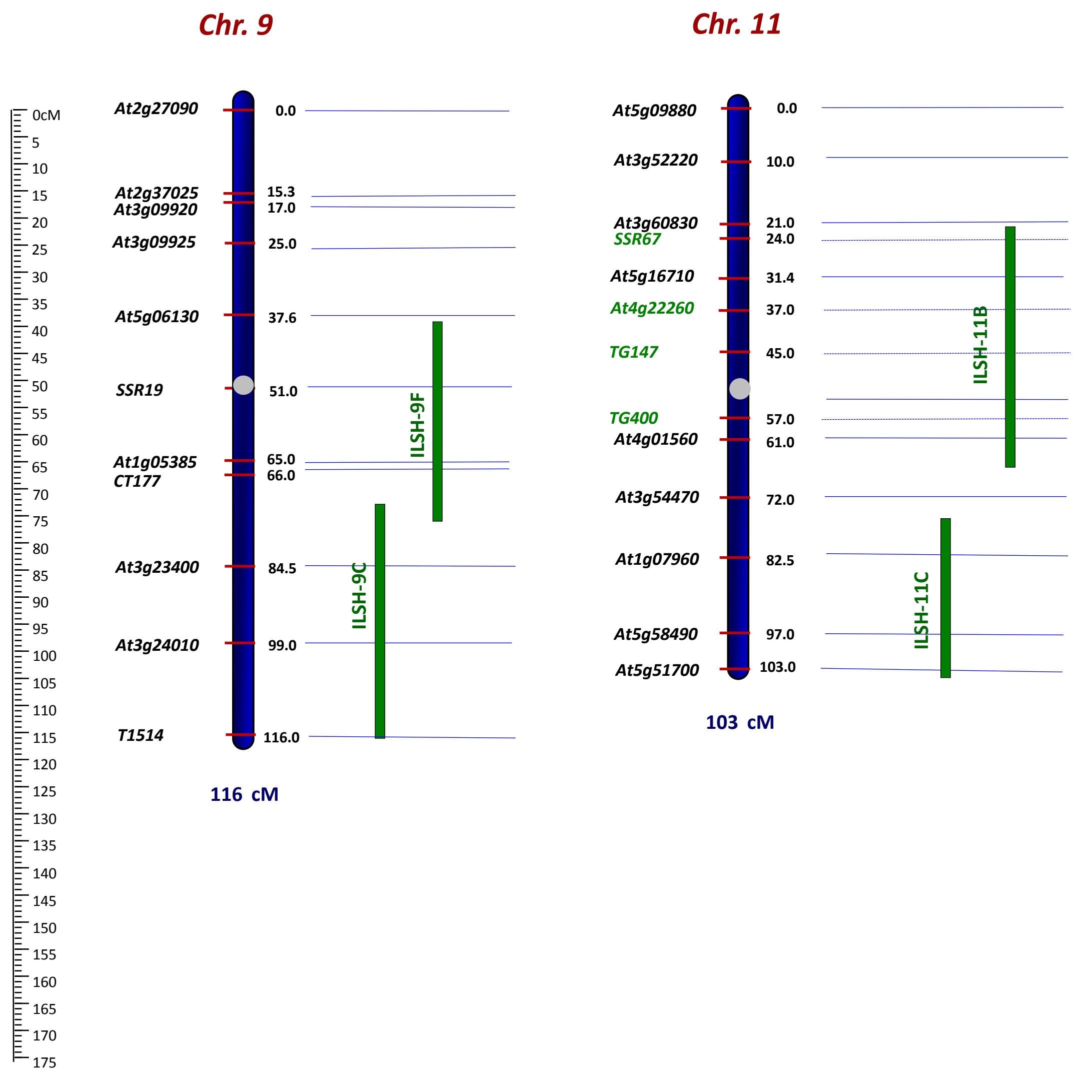

Chr. 9
Chr. 11
0cM
5
10
15
20
25
30
35
40
45
50
55
60
65
70
75
80
85
90
95
100
105
110
115
120
125
130
135
140
145
150
155
160
165
170
175
At2g27090 0.0
At2g37025 15.3
At3g09920 17.0
At3g09925 25.0
At5g06130 37.6
SSR19 51.0
At1g05385 65.0
CT177 66.0
At3g23400 84.5
At3g24010 99.0
T1514 116.0
ILSH-9F
ILSH-9C
116 cM
At5g09880 0.0
At3g52220 10.0
At3g60830 21.0
SSR67 24.0
At5g16710 31.4
At4g22260 37.0
TG147 45.0
TG400 57.0
At4g01560 61.0
At3g54470 72.0
At1g07960 82.5
At5g58490 97.0
At5g51700 103.0
ILSH-11B
ILSH-11C
103 cM

Figure S2. Histogram of the distribution of each volatile compound in the RIL population in seasons 1, 2 and 3.

RILs season 1

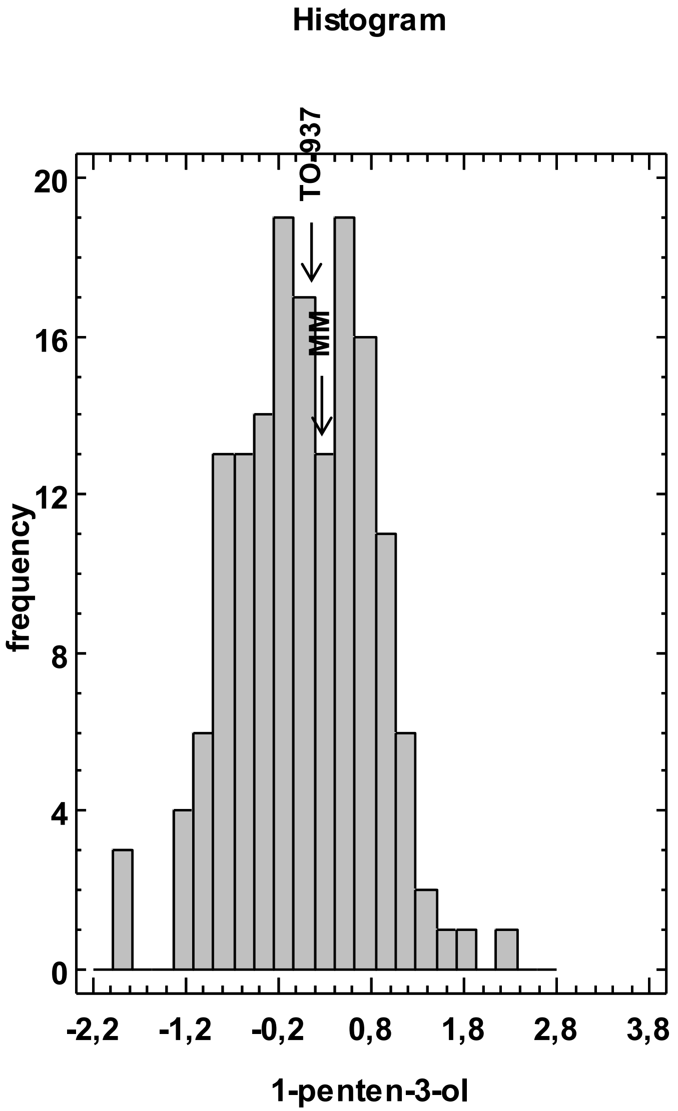

RILs season 1

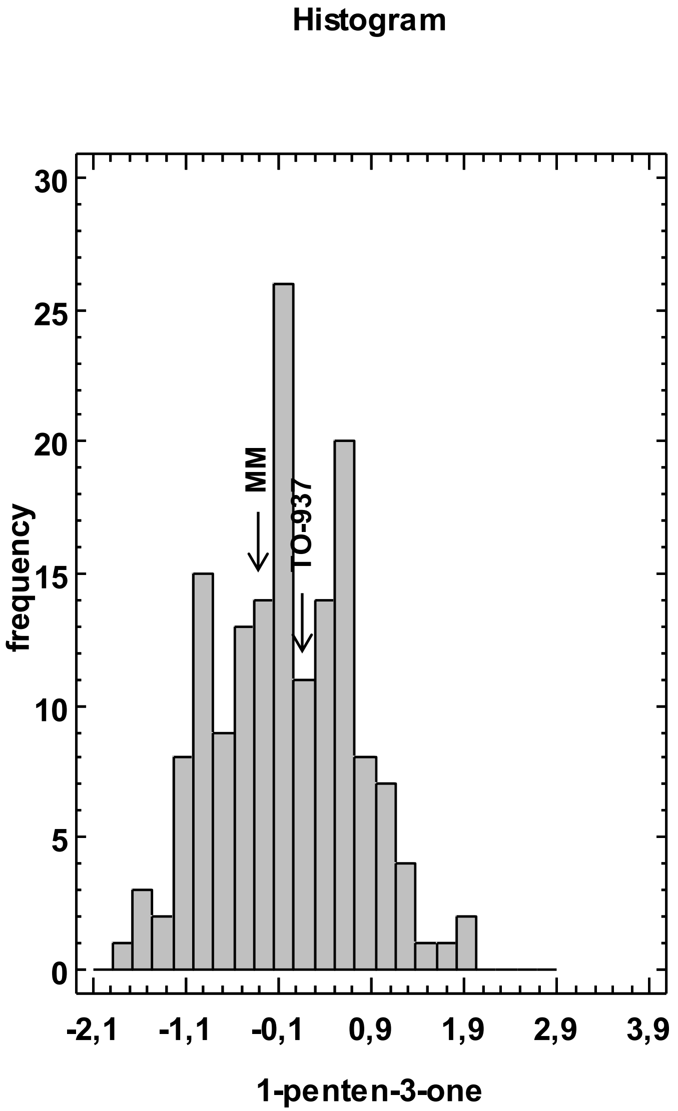

RILs season 1

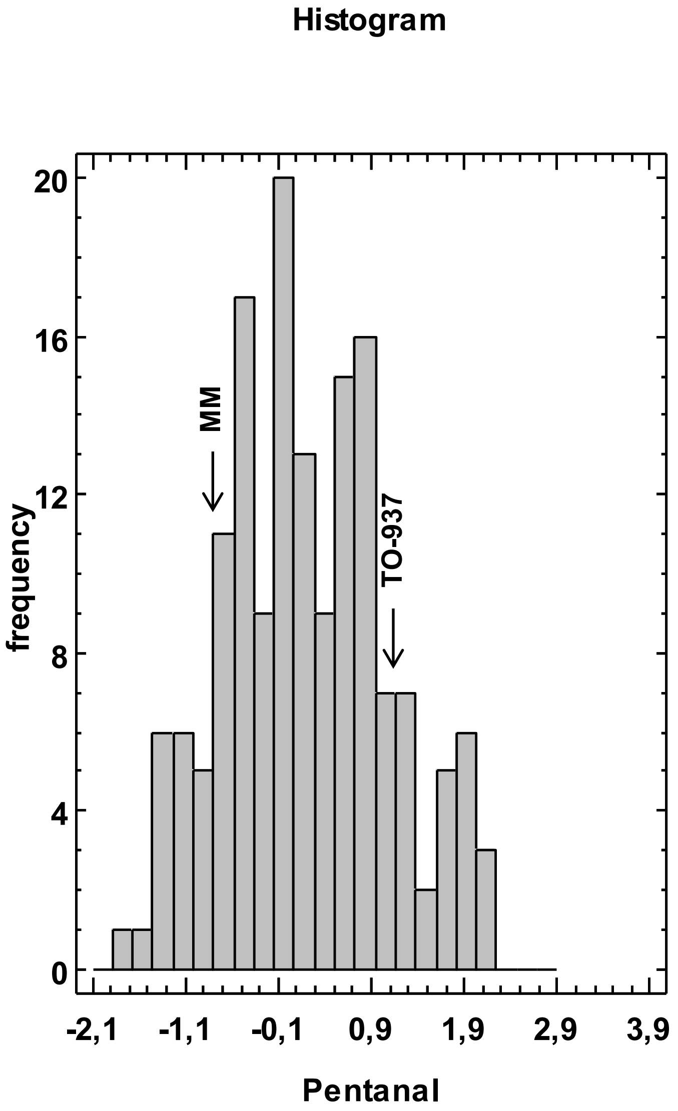

RILs season 1

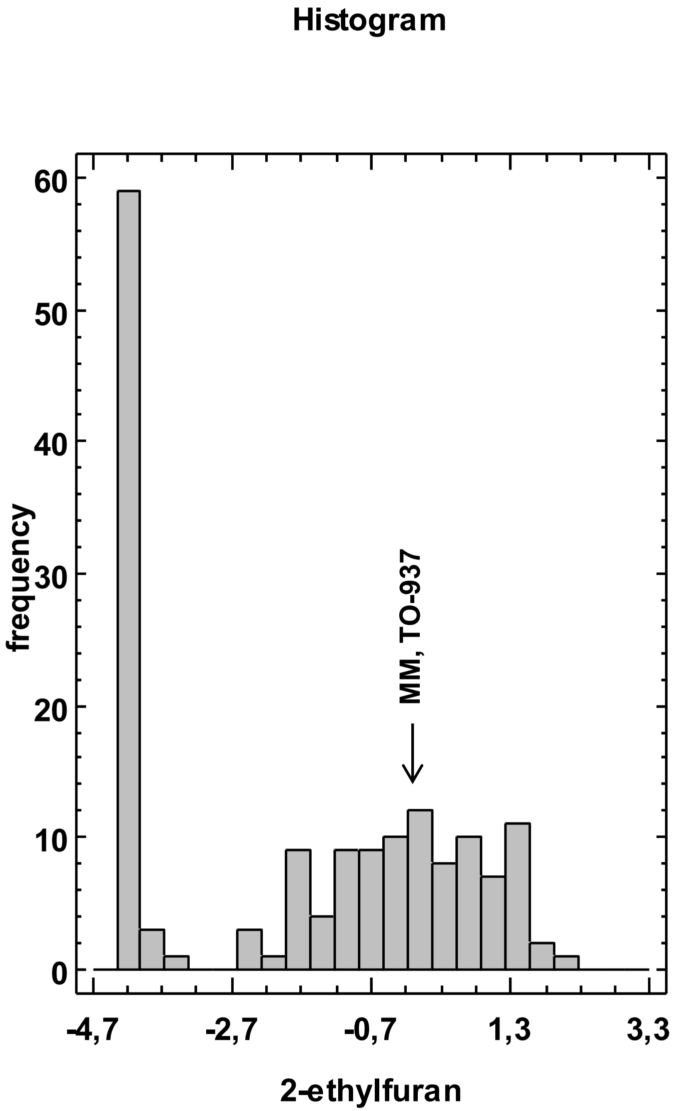

RILs season 1

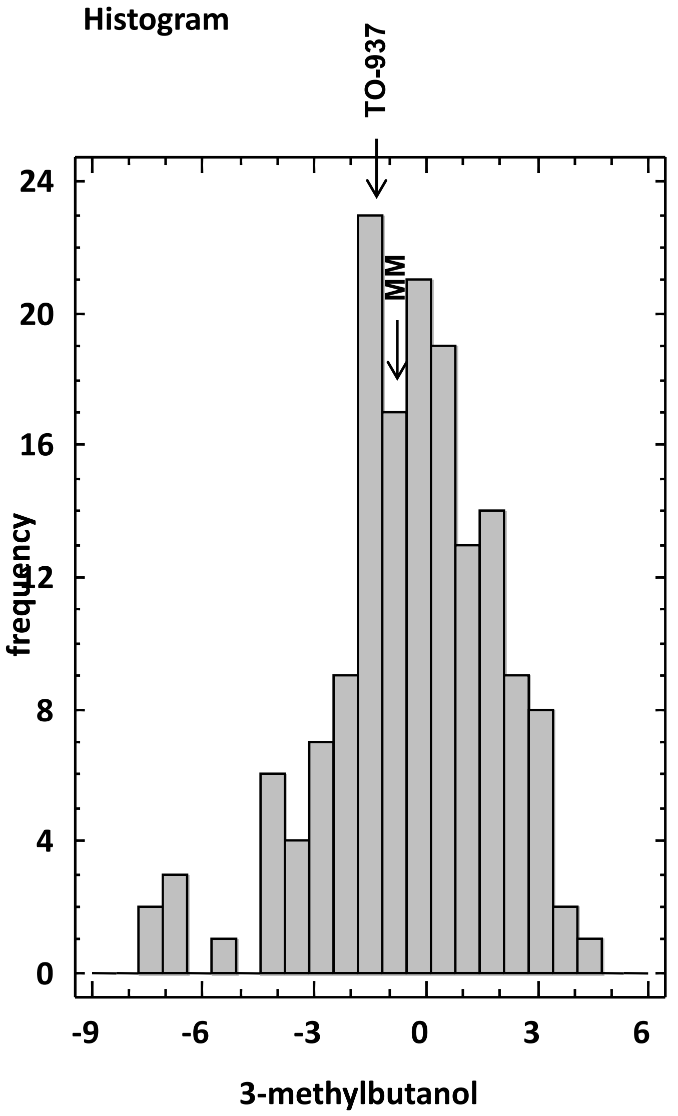

RILs season 1

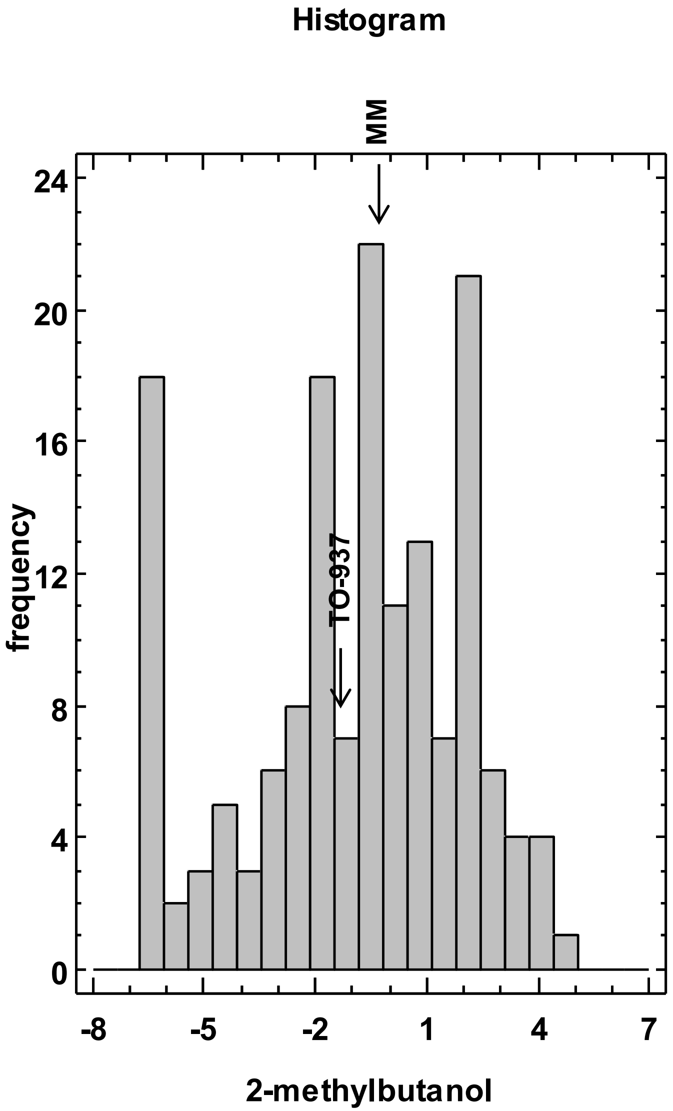

RILs season 1

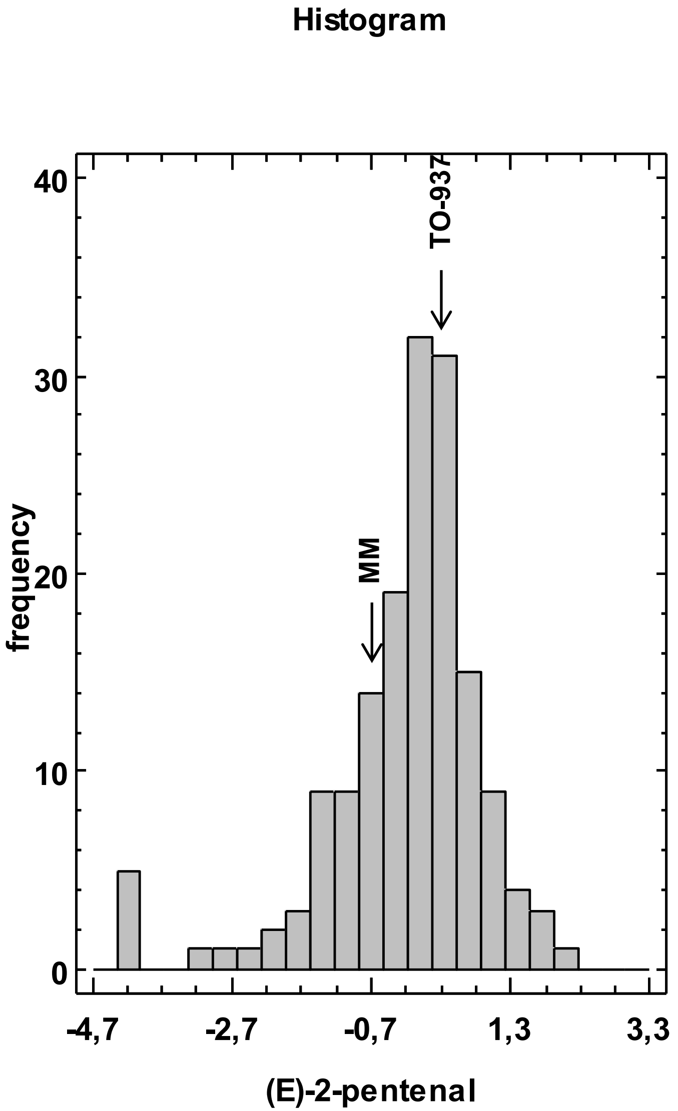

RILs season 1

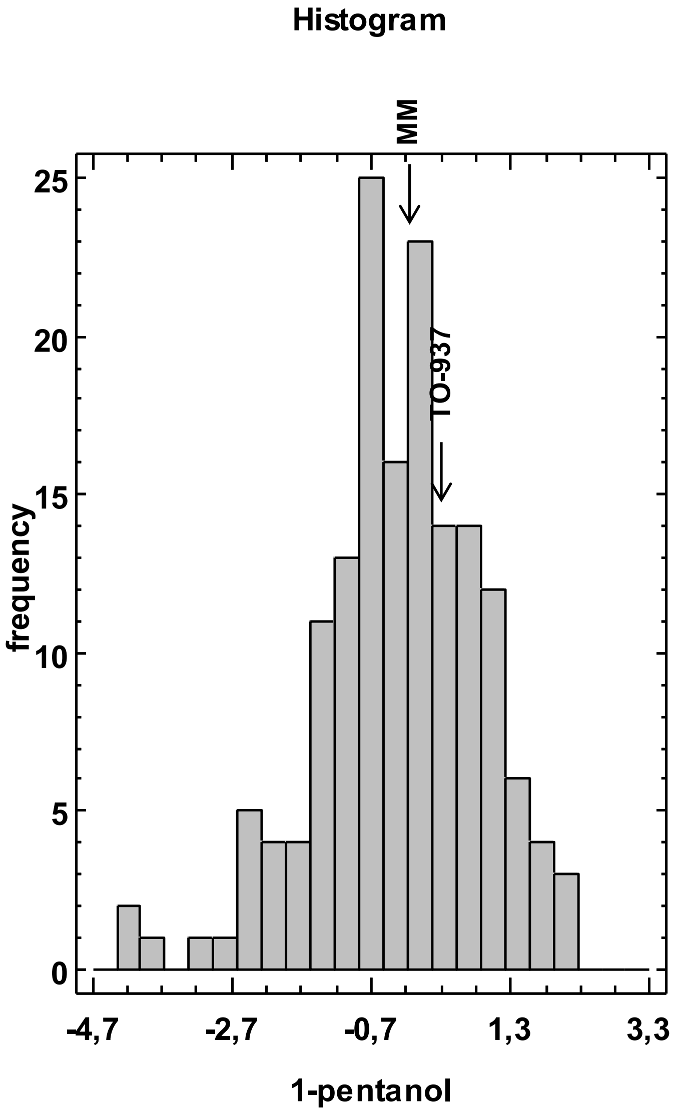

RILs season 1

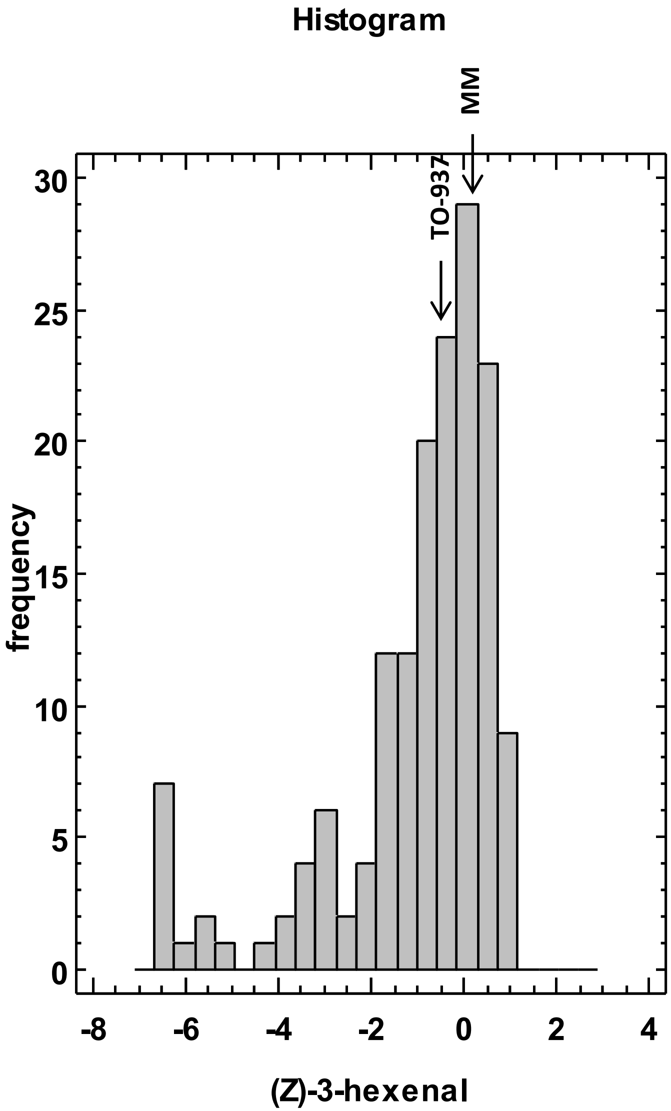

RILs season 1

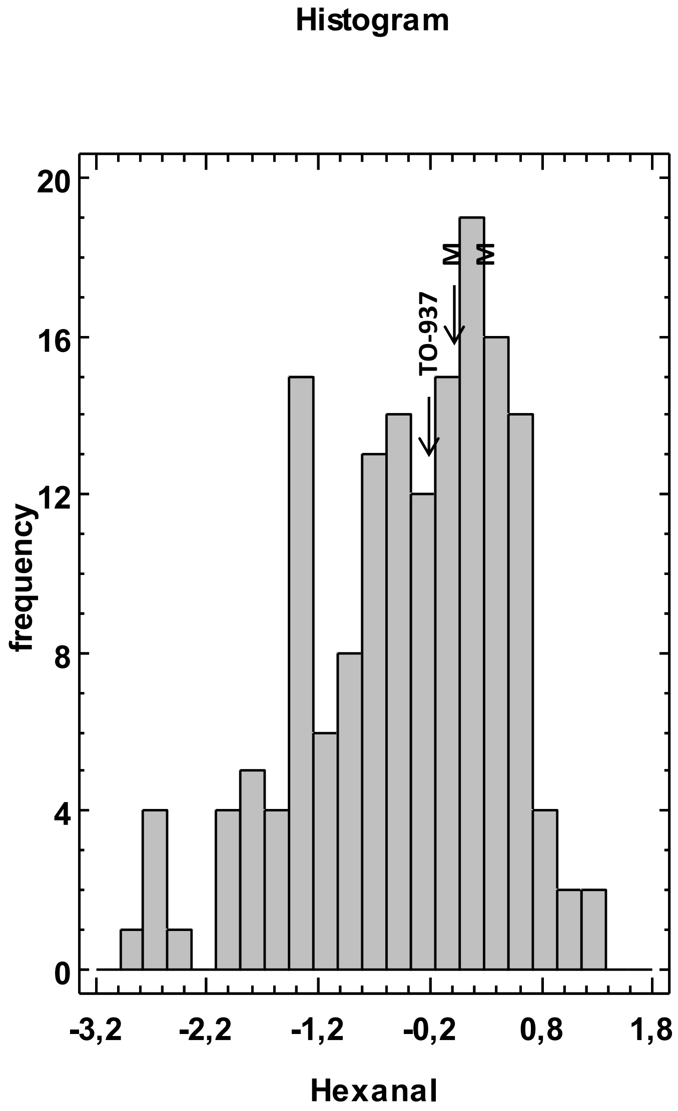

RILs season 1

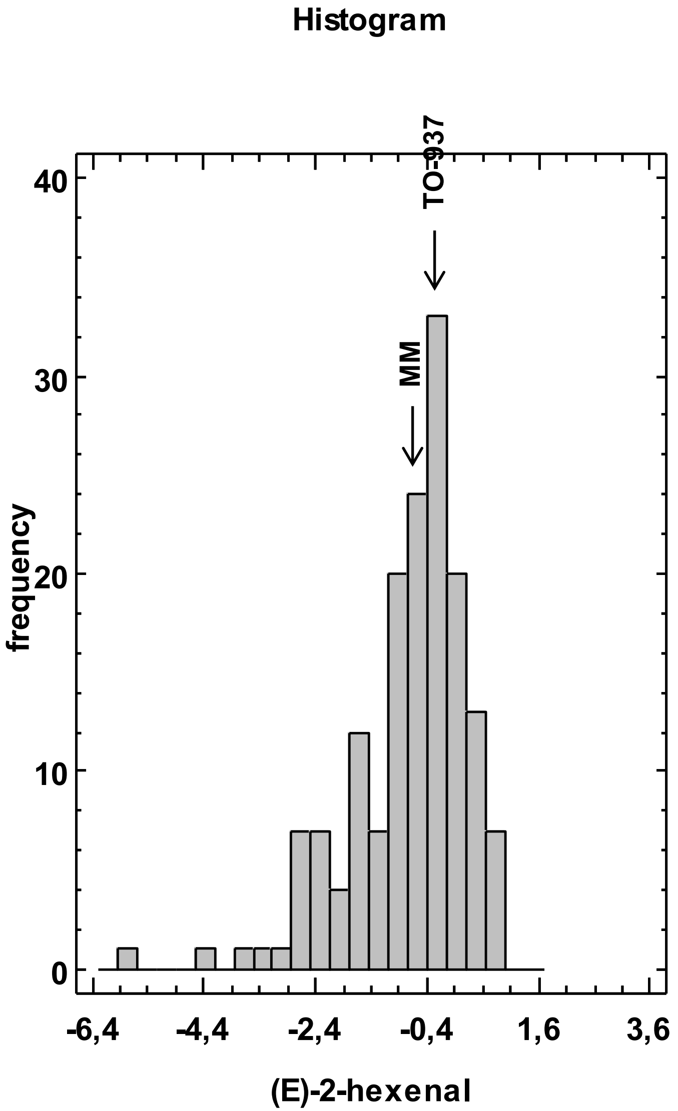

RILs season 1

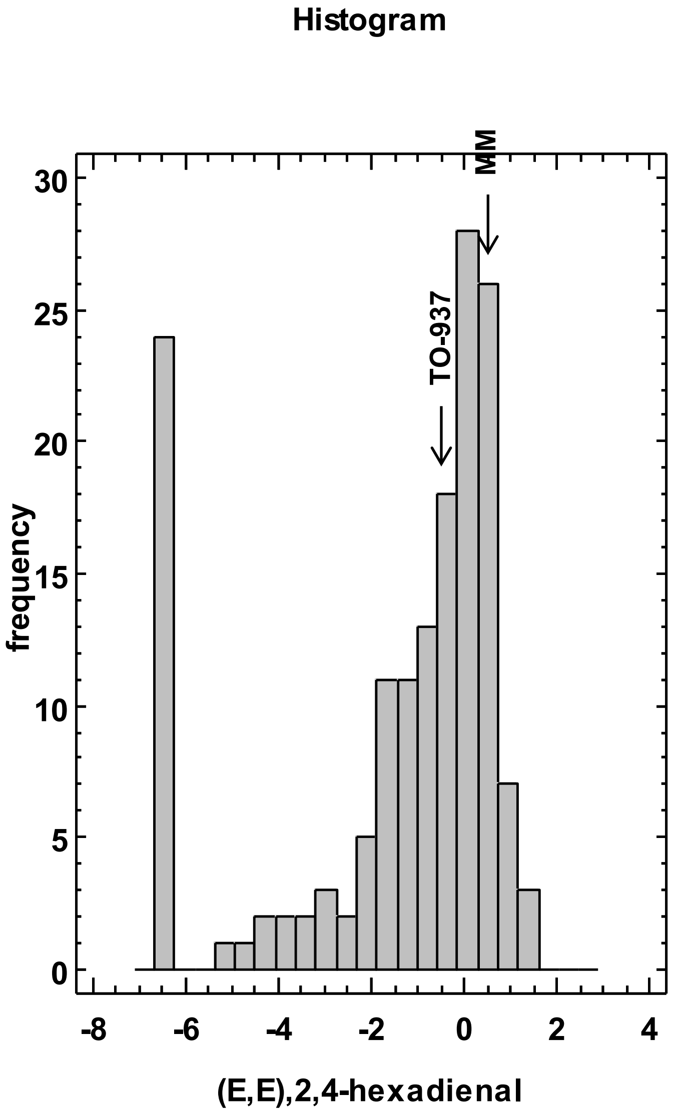

RILs season 1

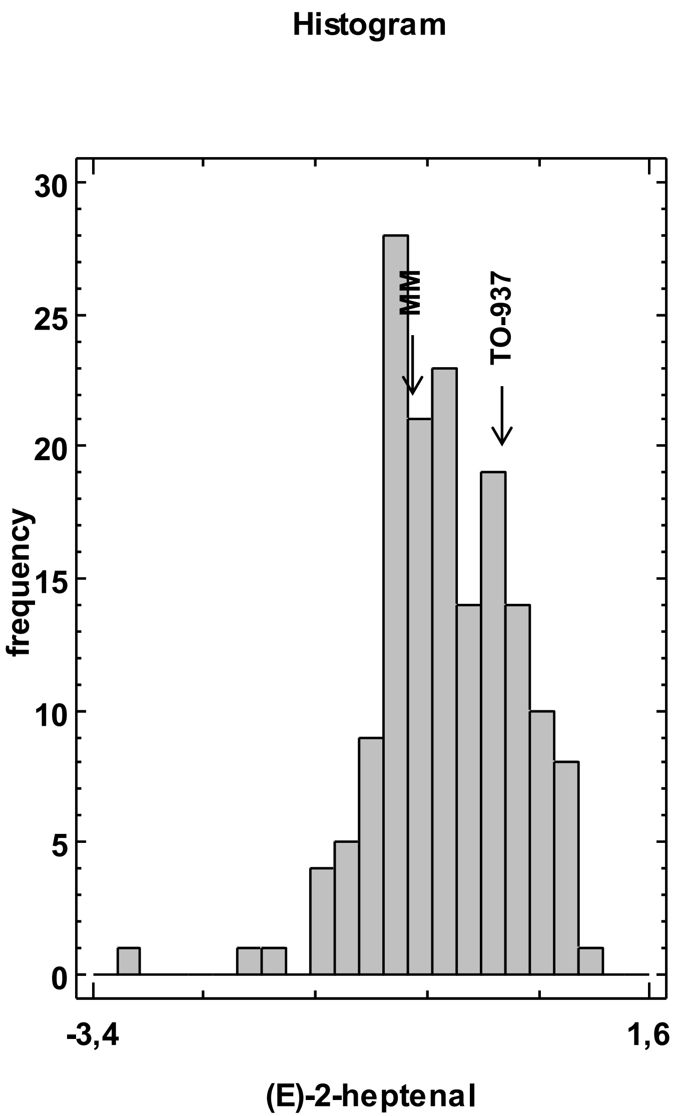

RILs season 1

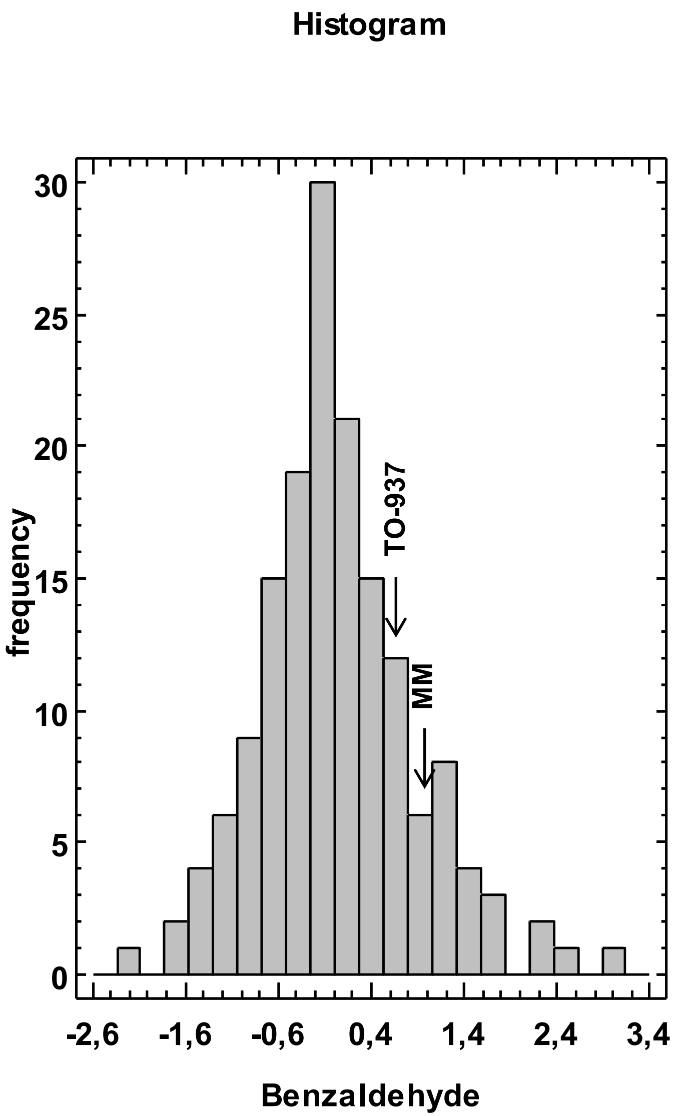

RILs season 1

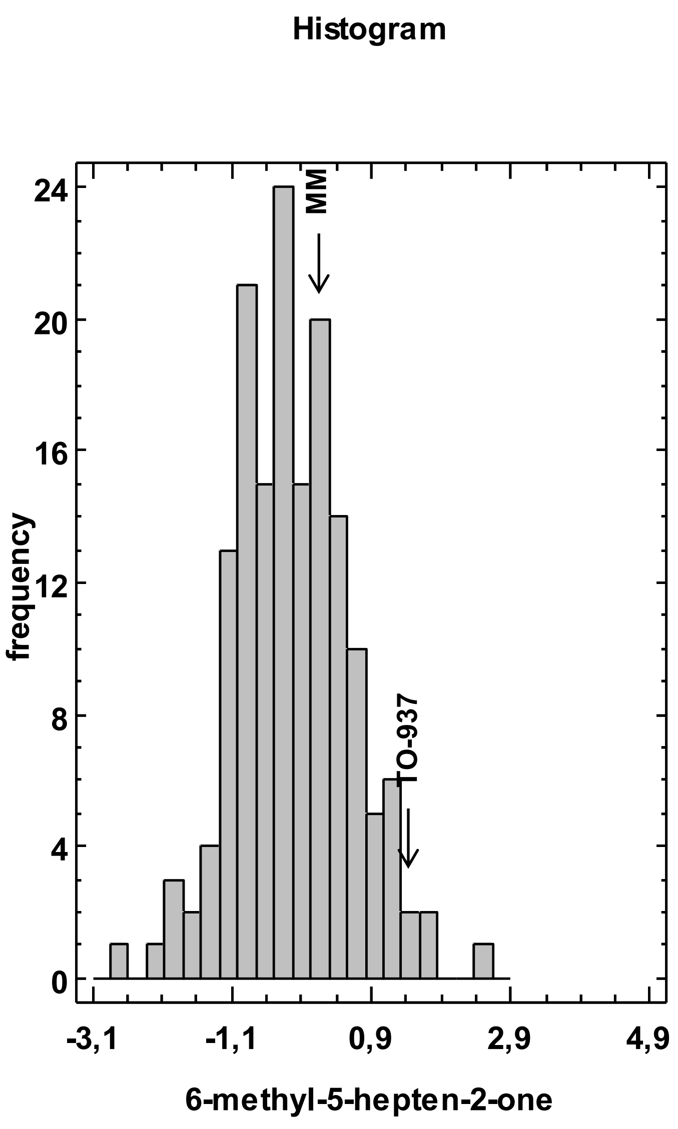

RILs season 1

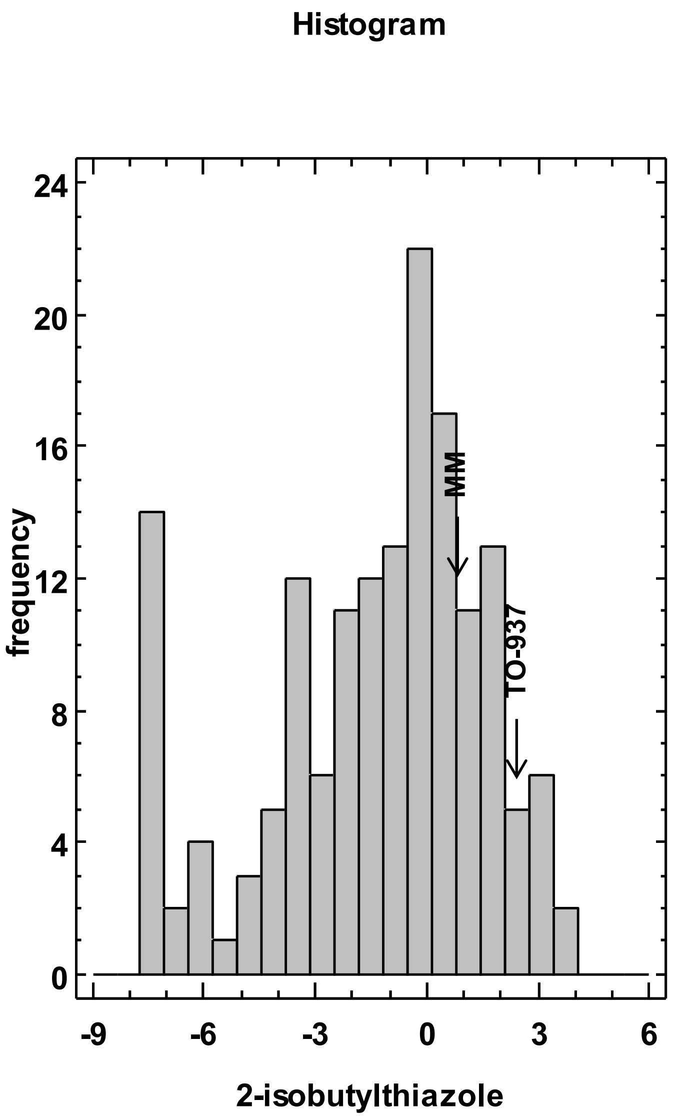

RILs season 1

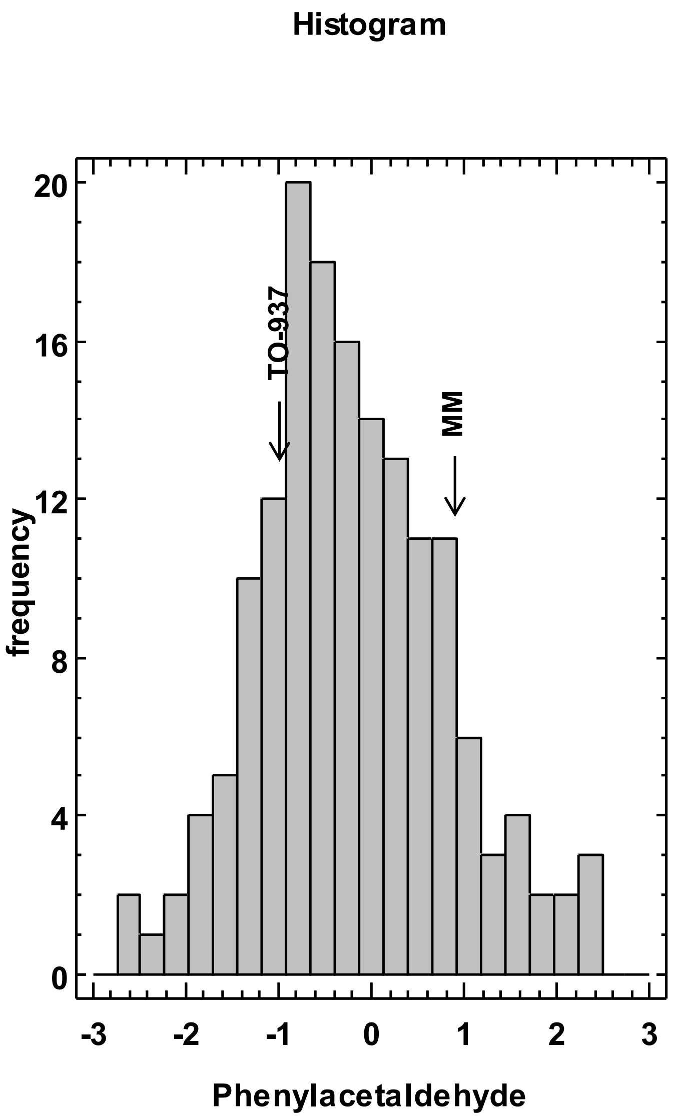

RILs season 1

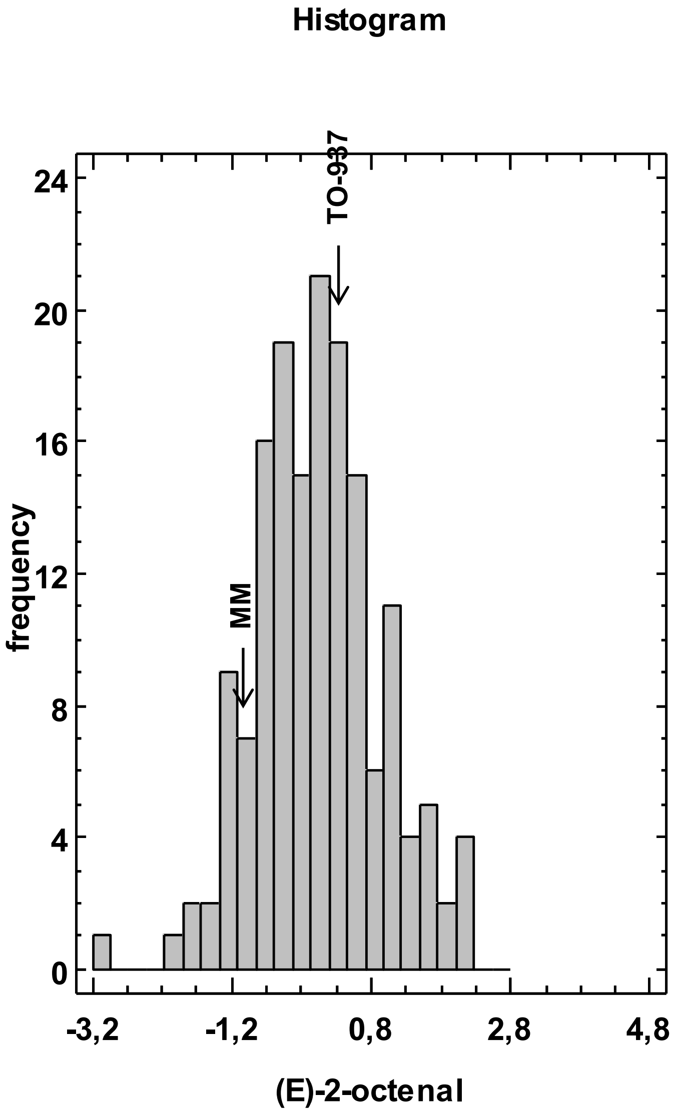

RILs season 1

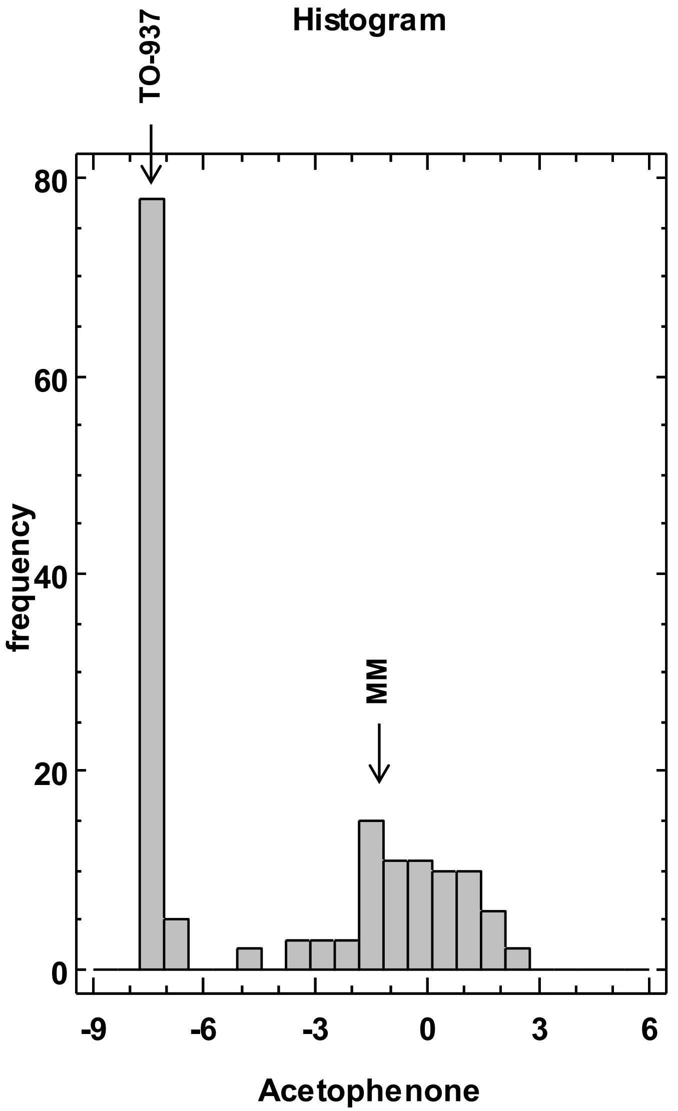

RILs season 1

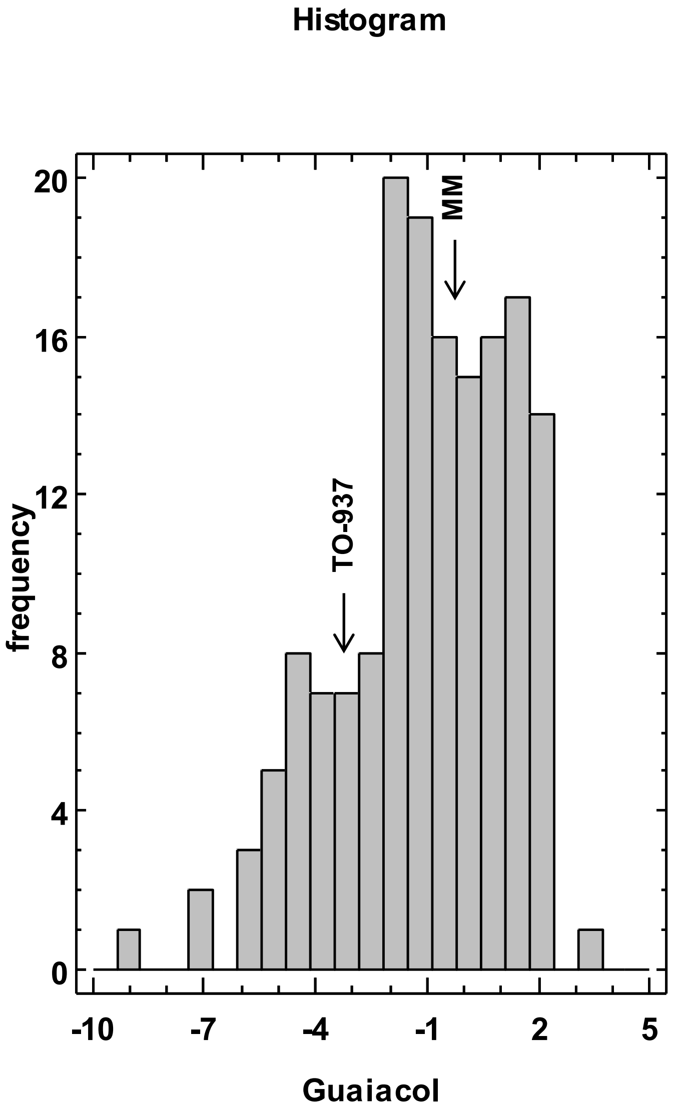

RILs season 1

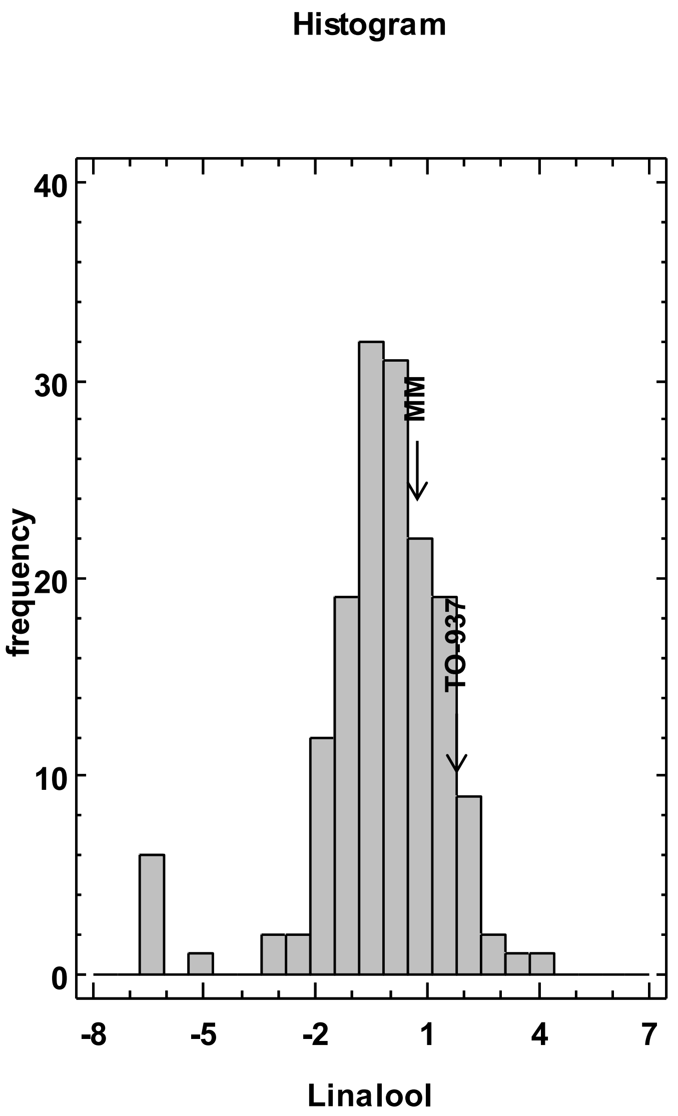

RILs season 1

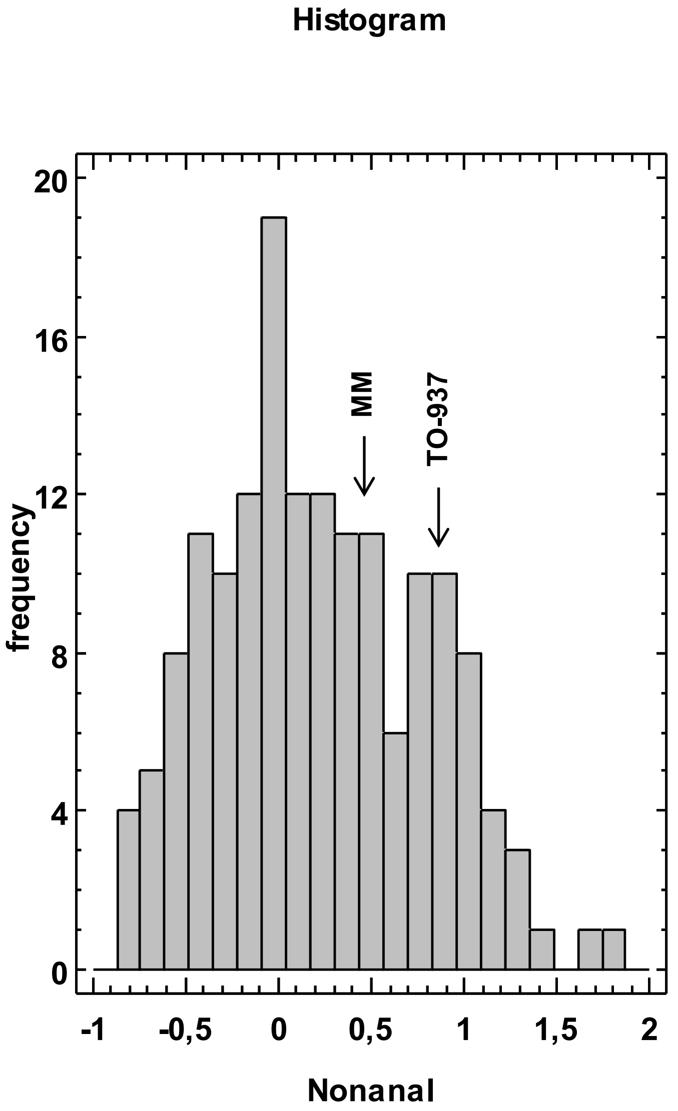

RILs season 1

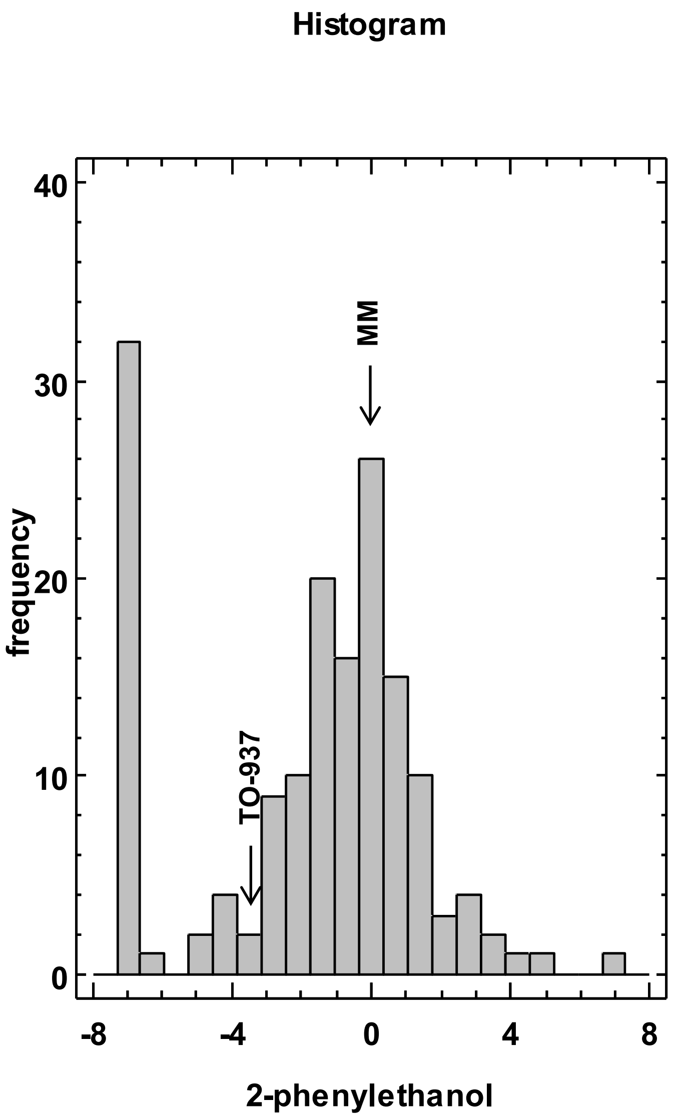

RILs season 1

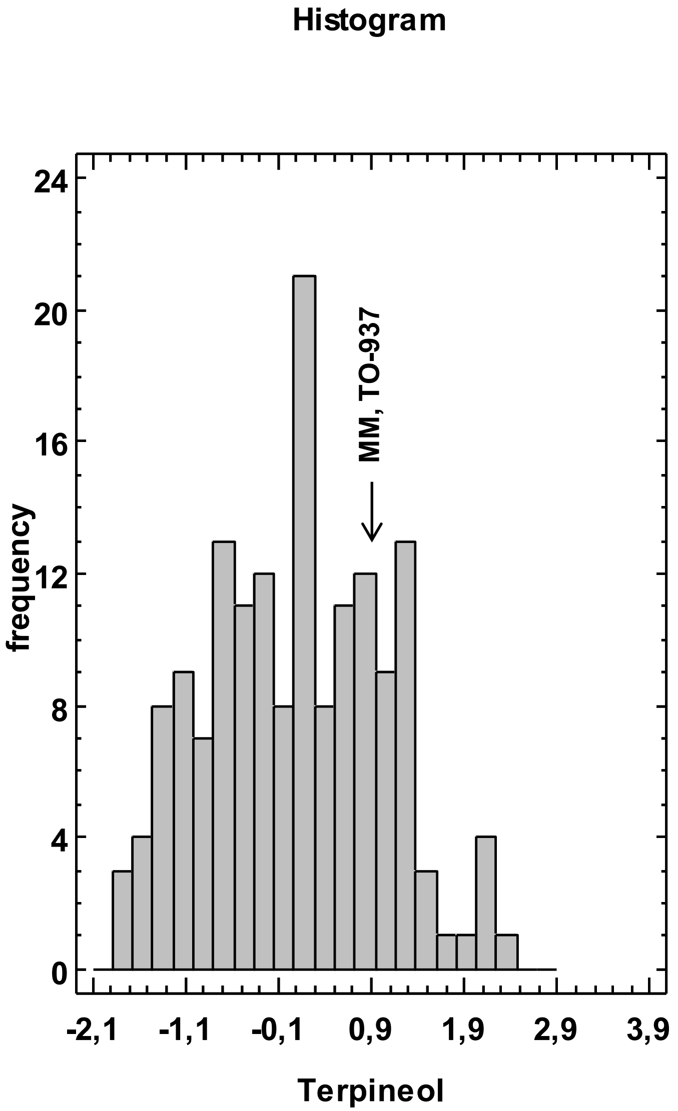

RILs season 1

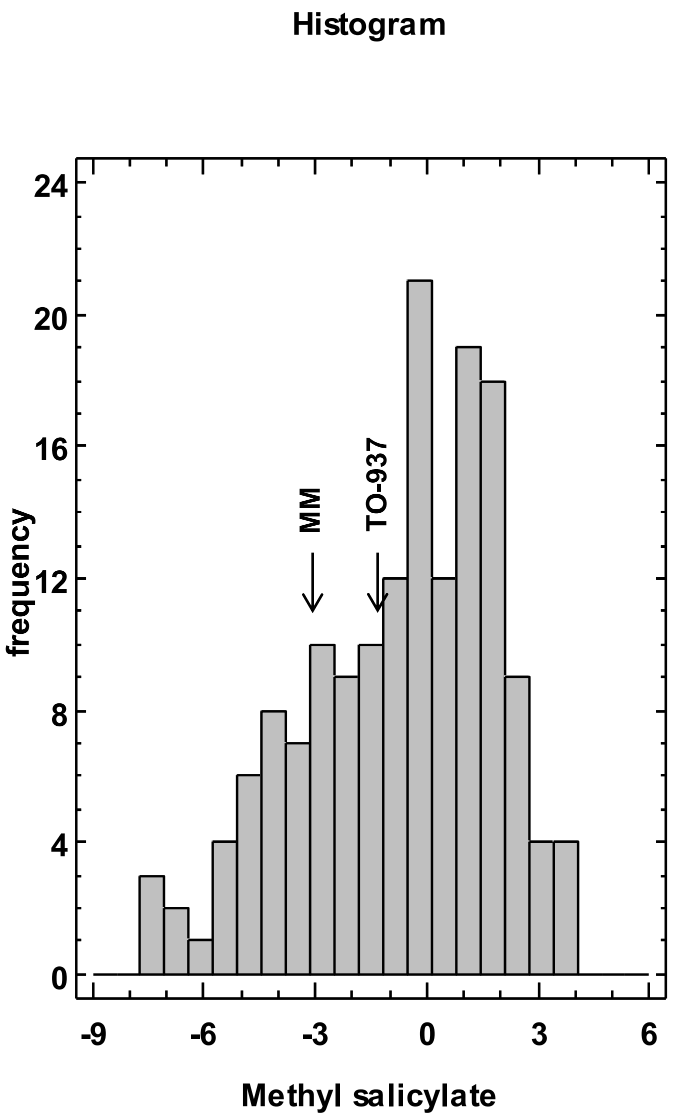

RILs season 1

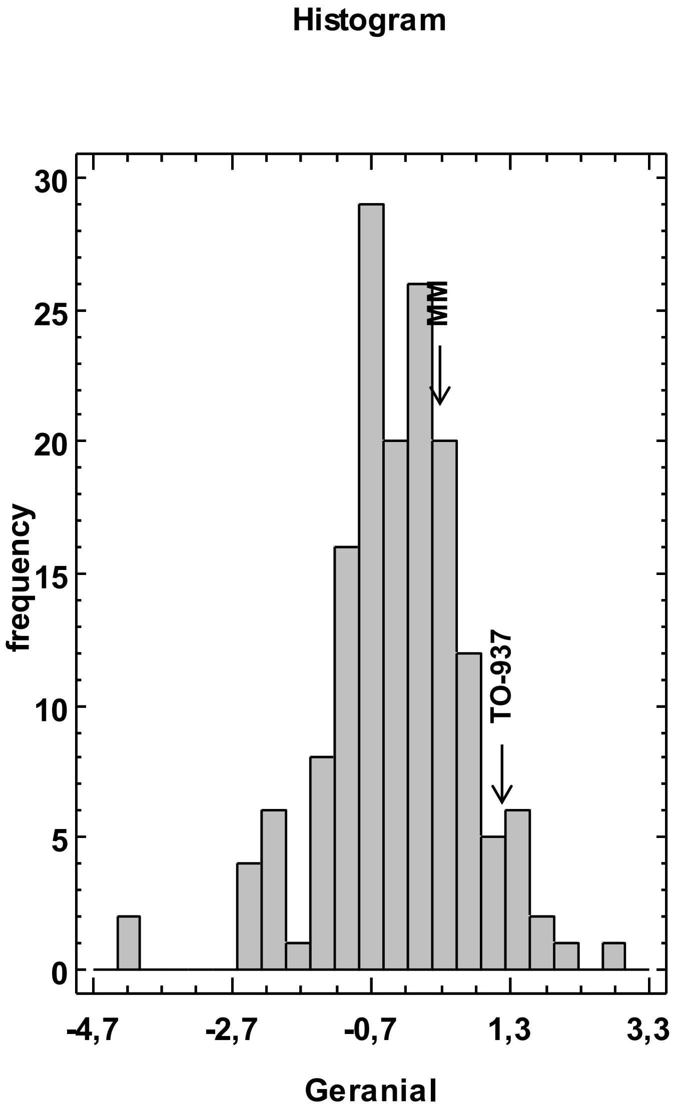

RILs season 1

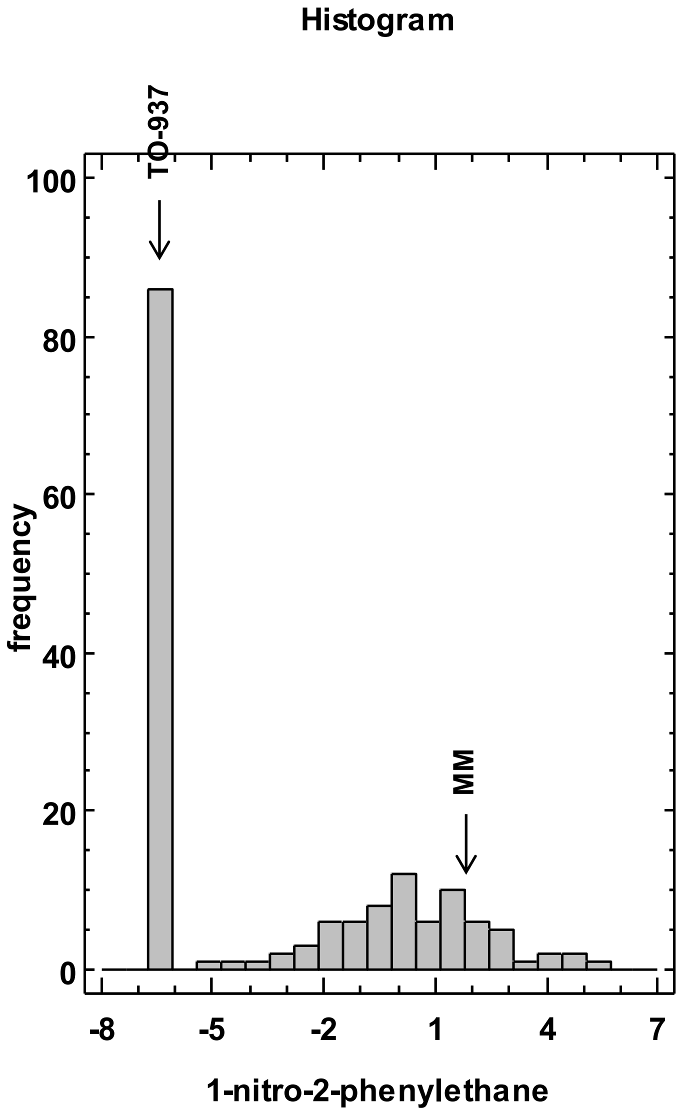

RILs season 1

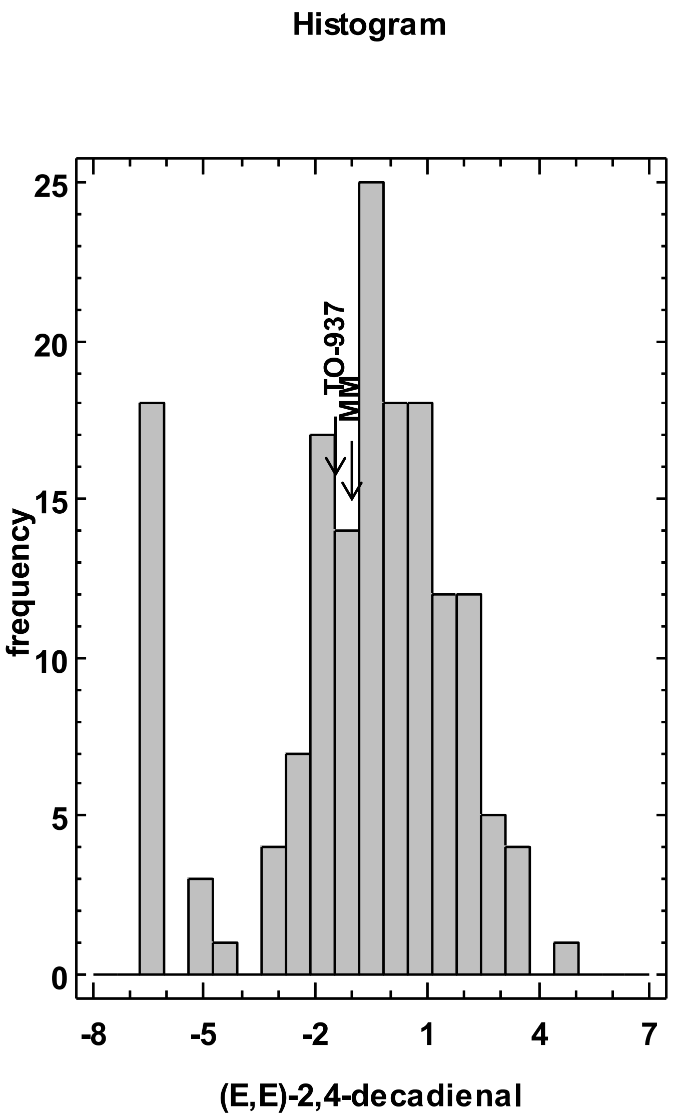

RILs season 1

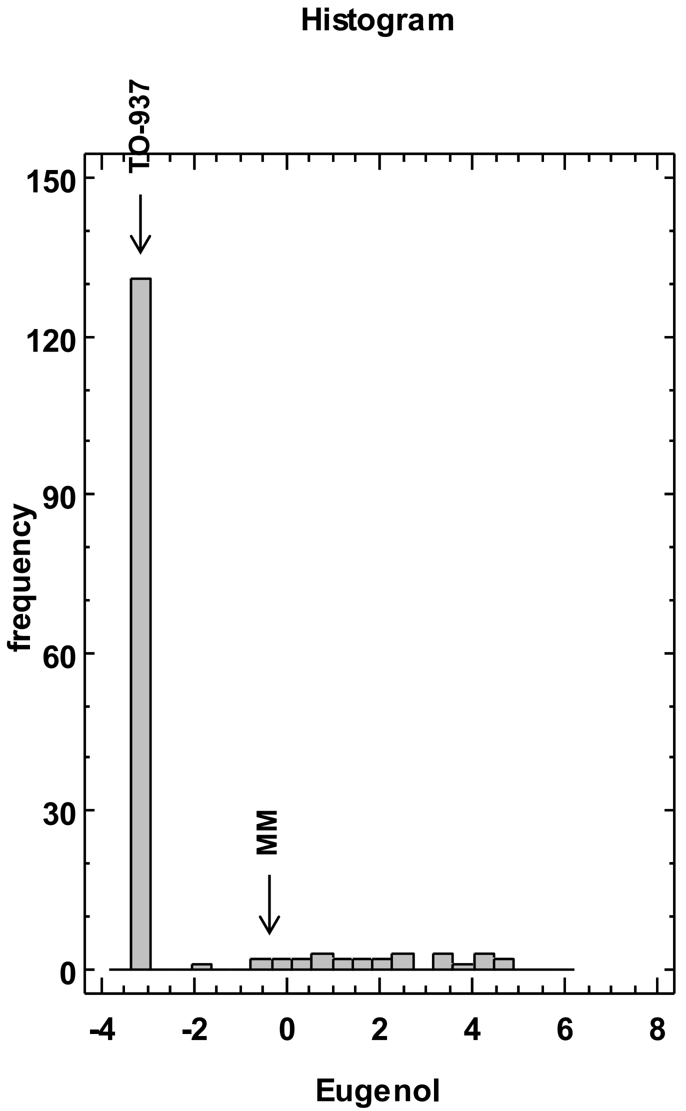

RILs season 1

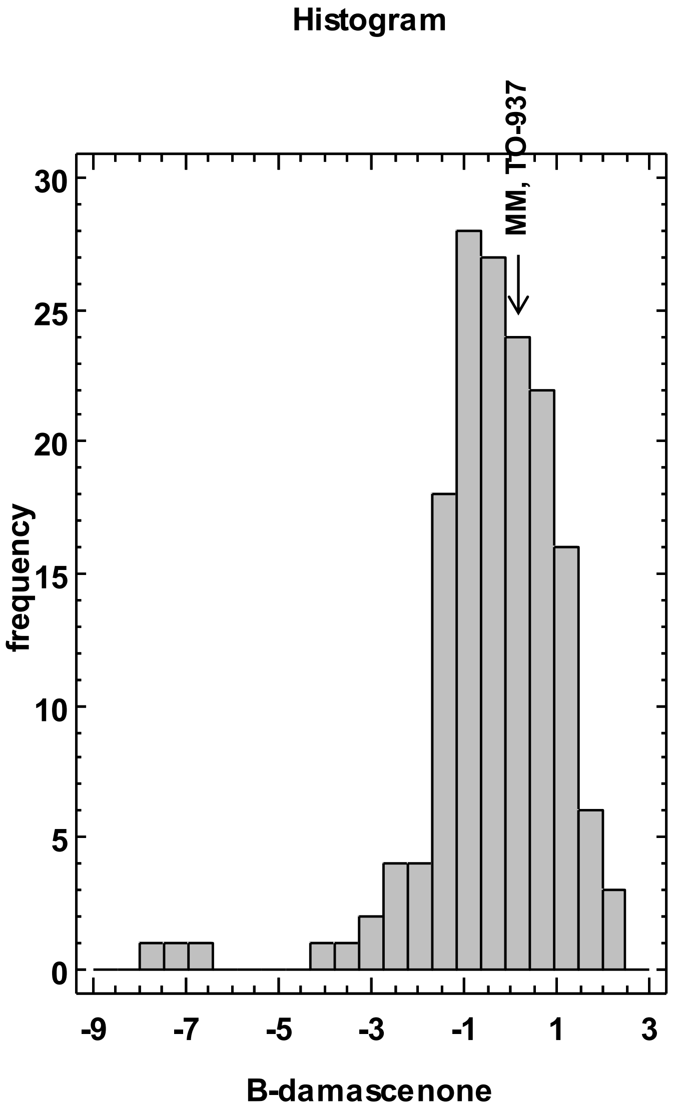

RILs season 1

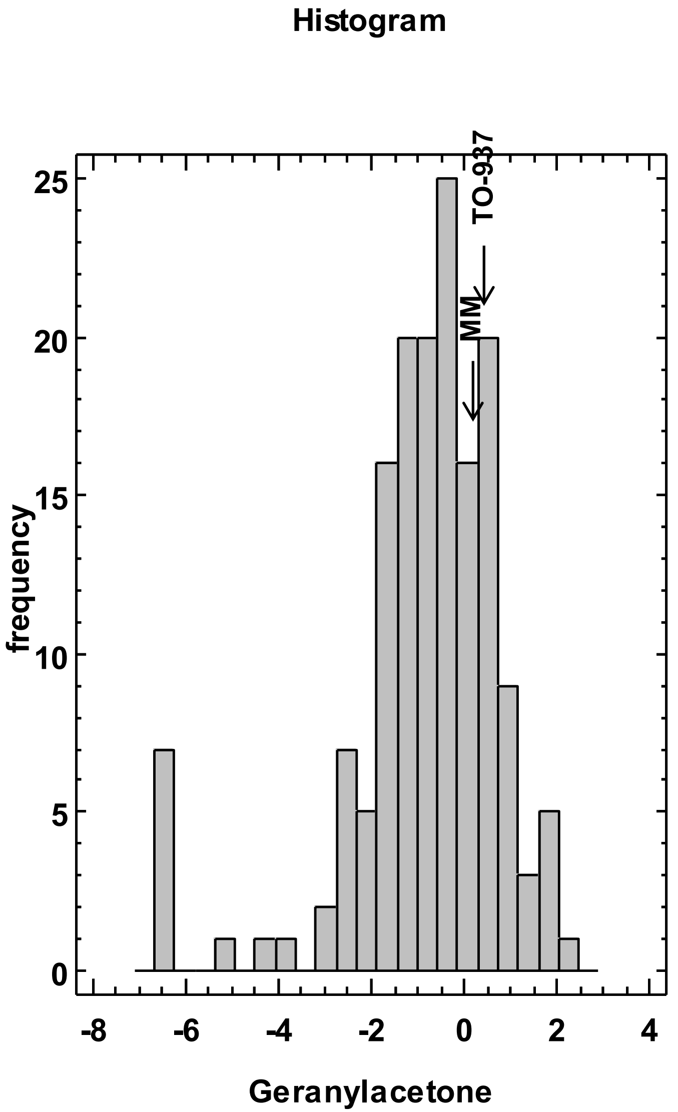

RILs season 2

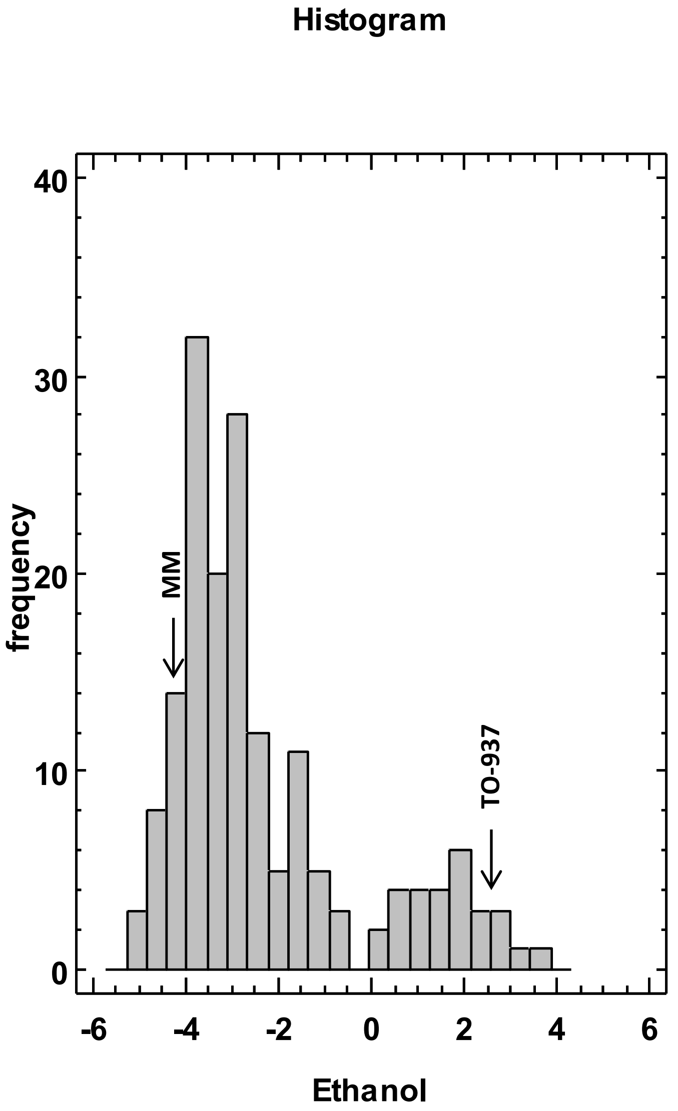

RILs season 2

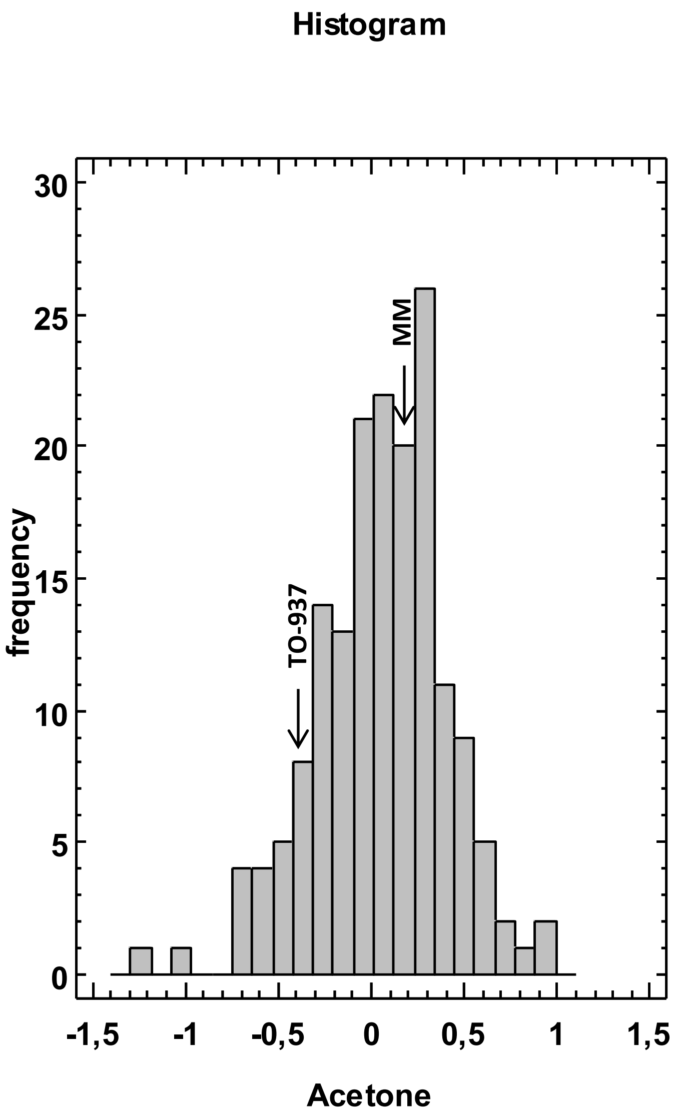

RILs season 2

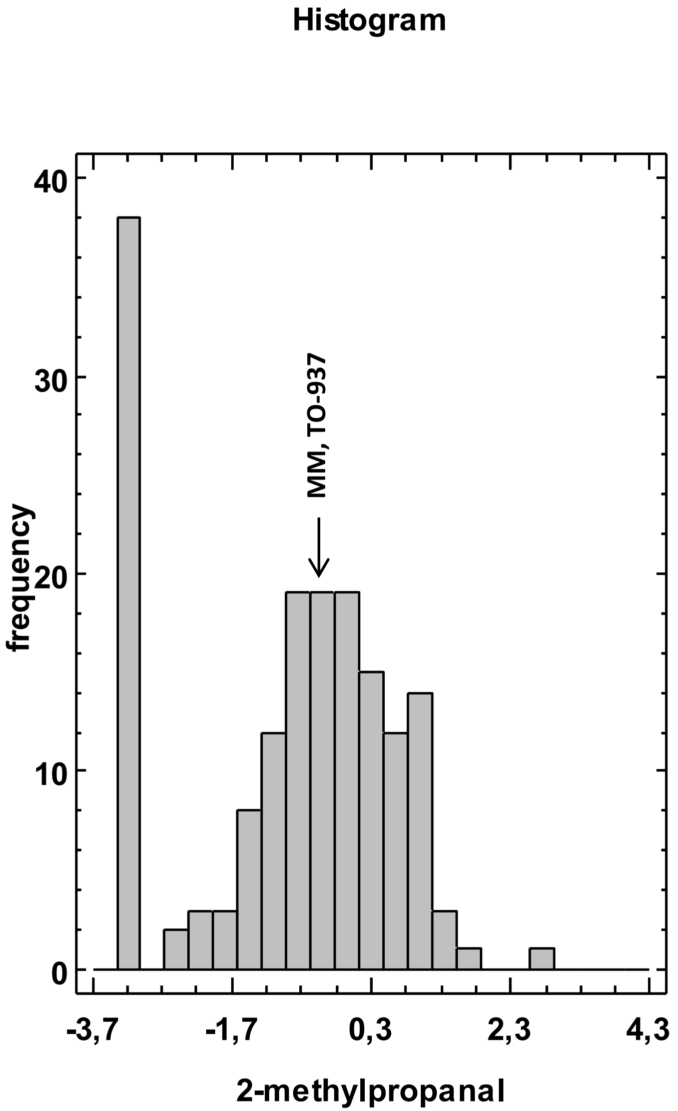

RILs season 2

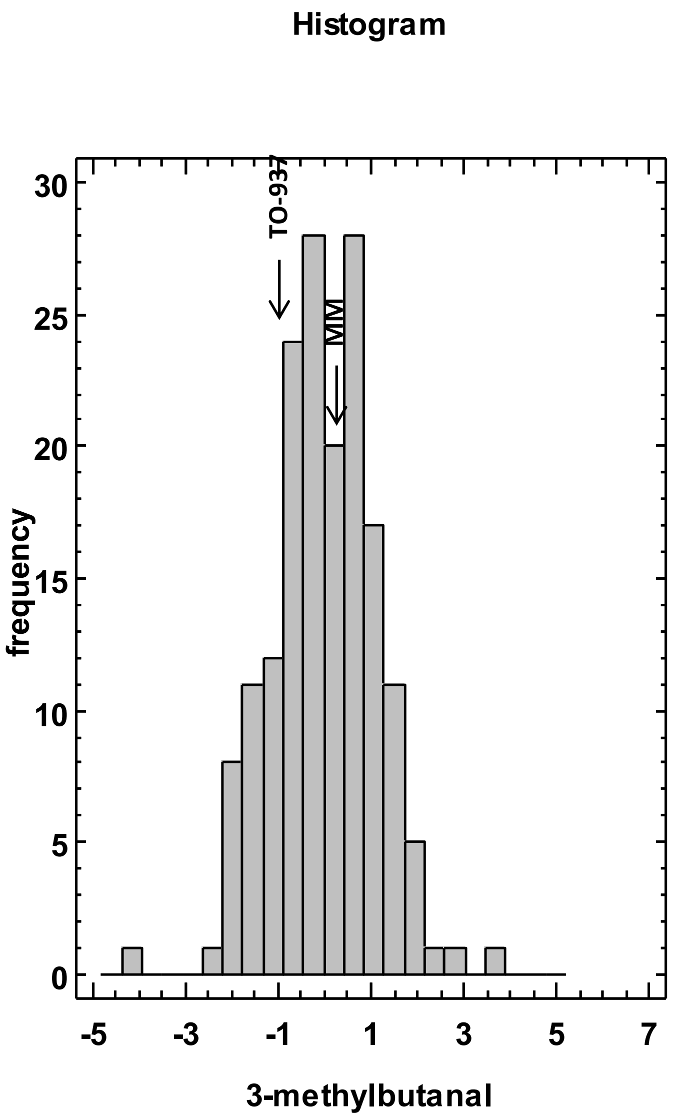

RILs season 2

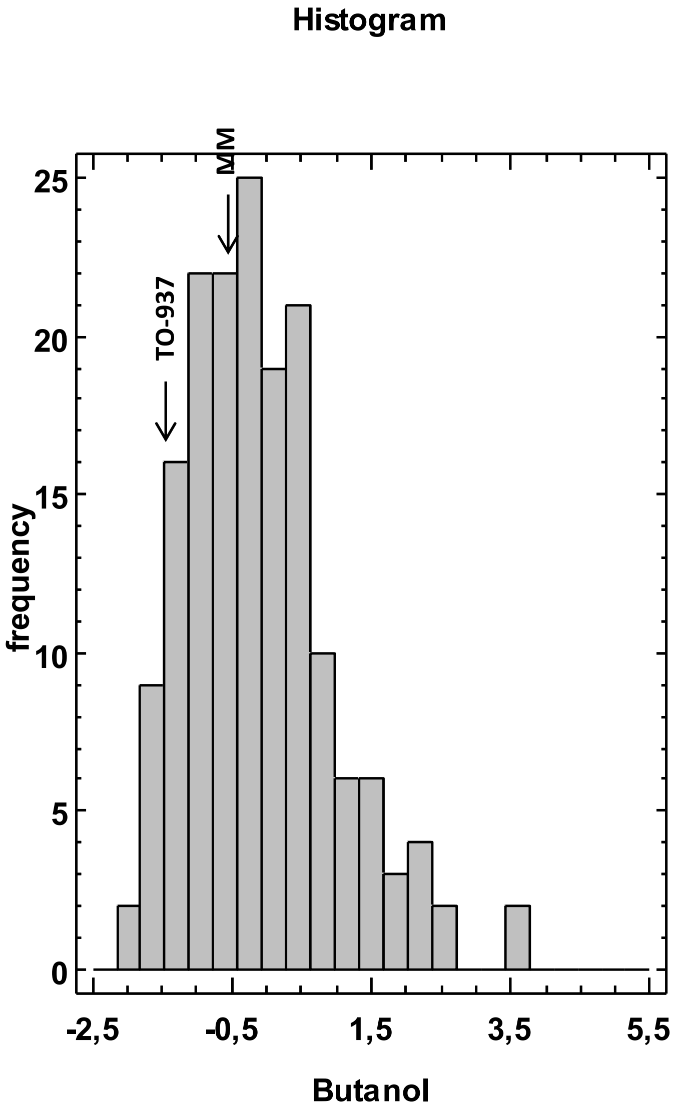

RILs season 2

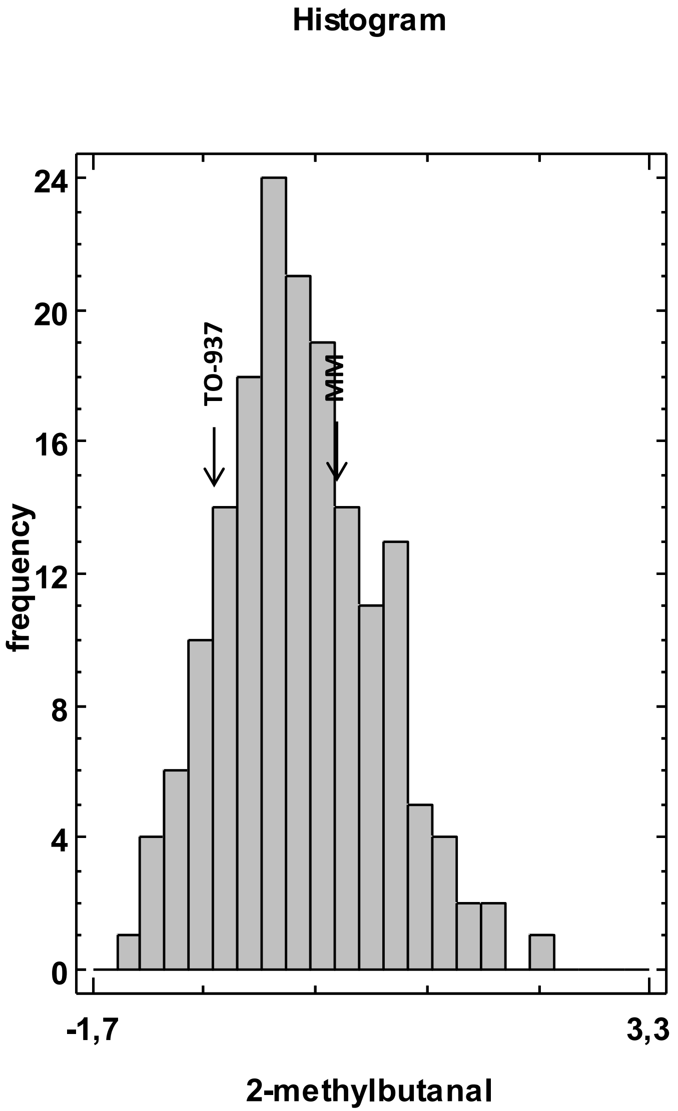

RILs season 2

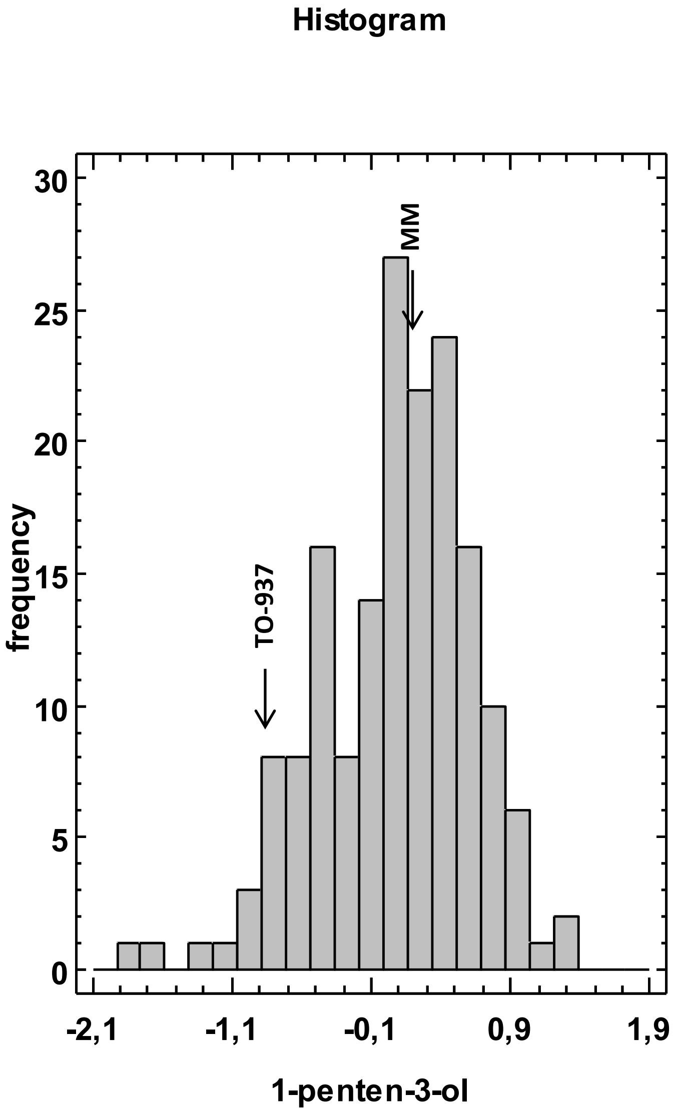

RILs season 2

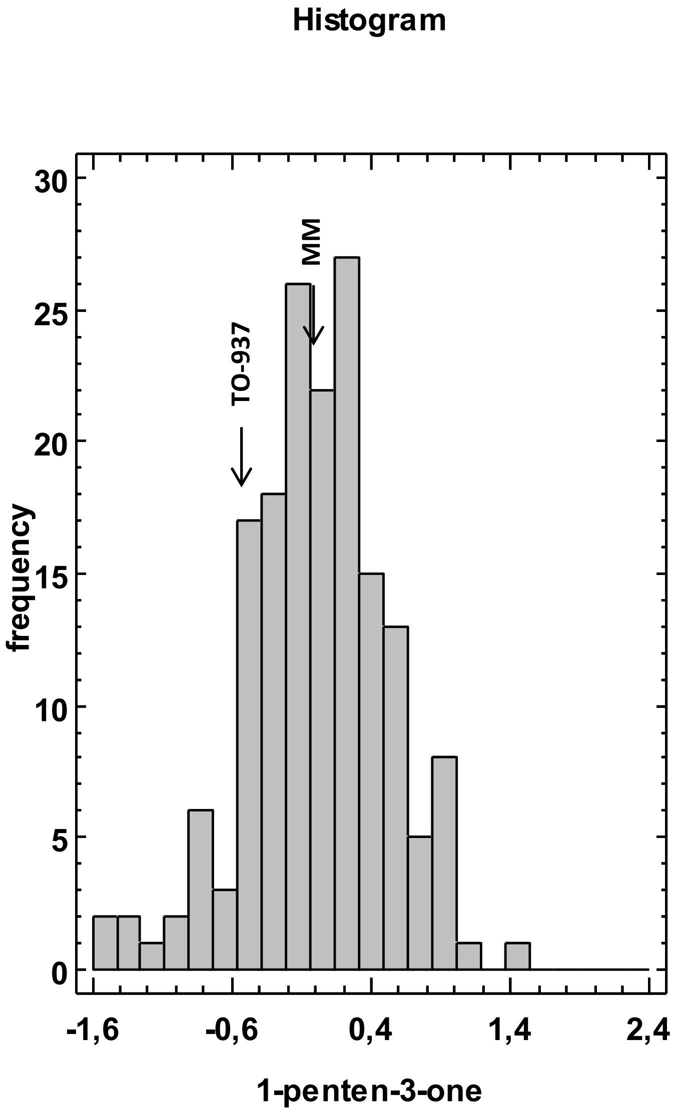

RILs season 2

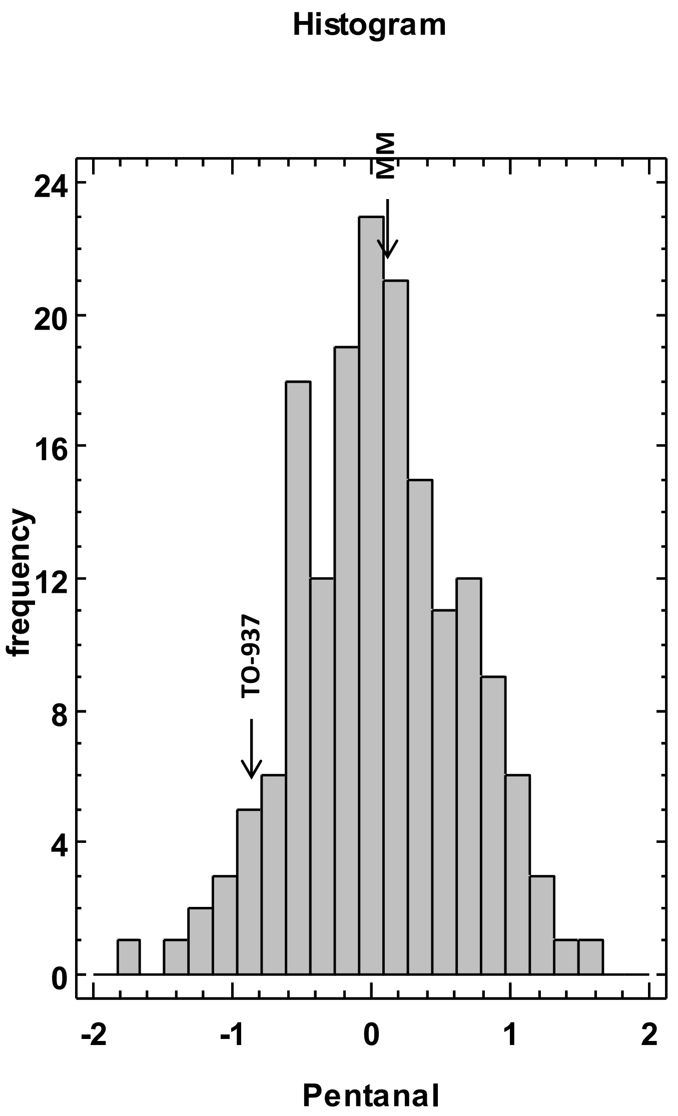

RILs season 2

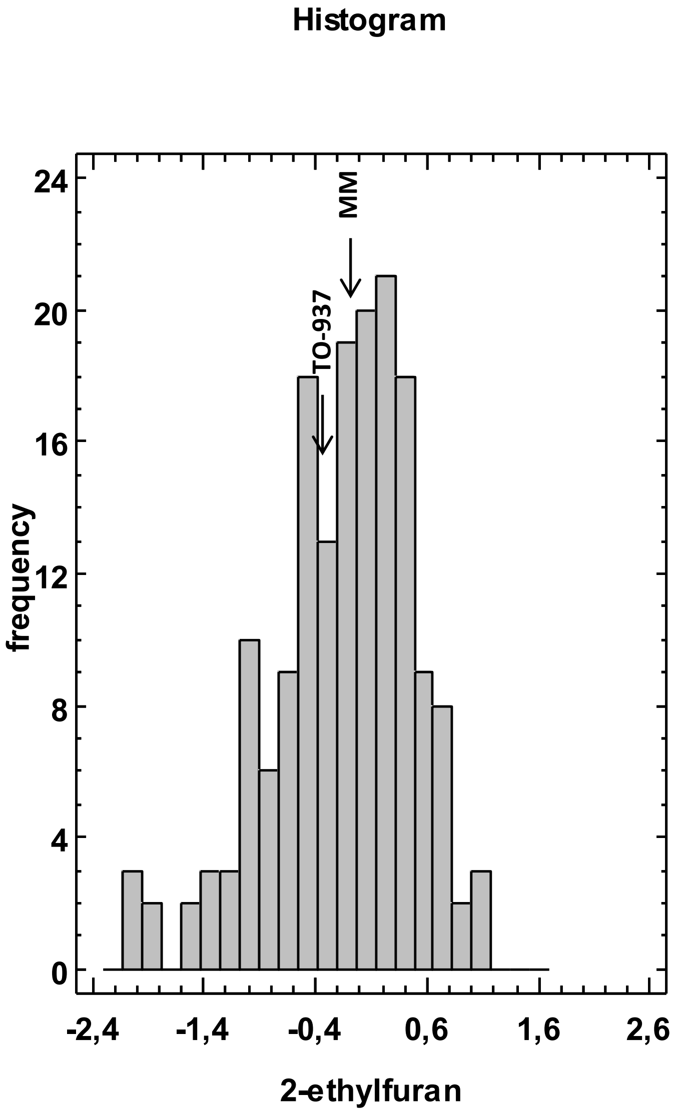

RILs season 2

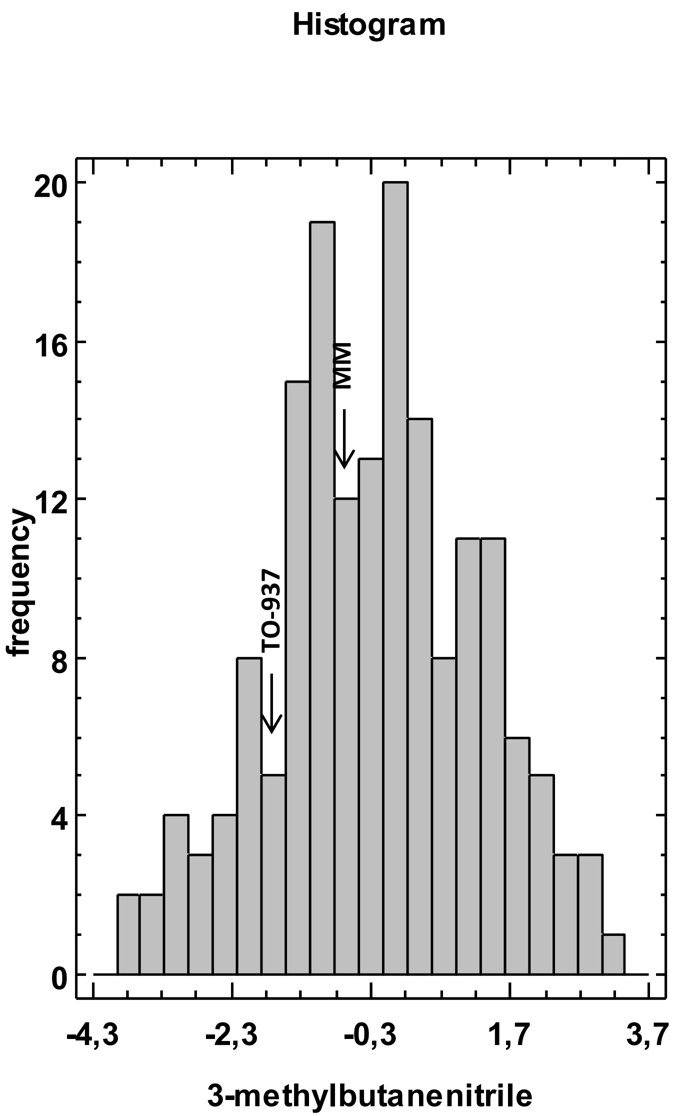

RILs season 2

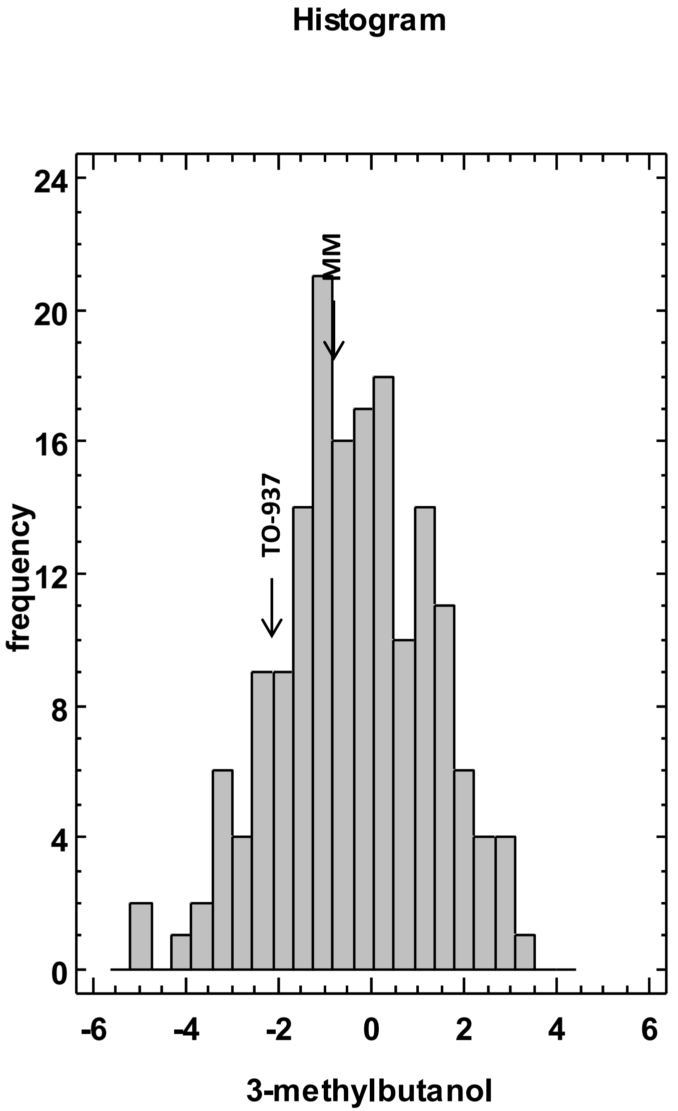

RILs season 2

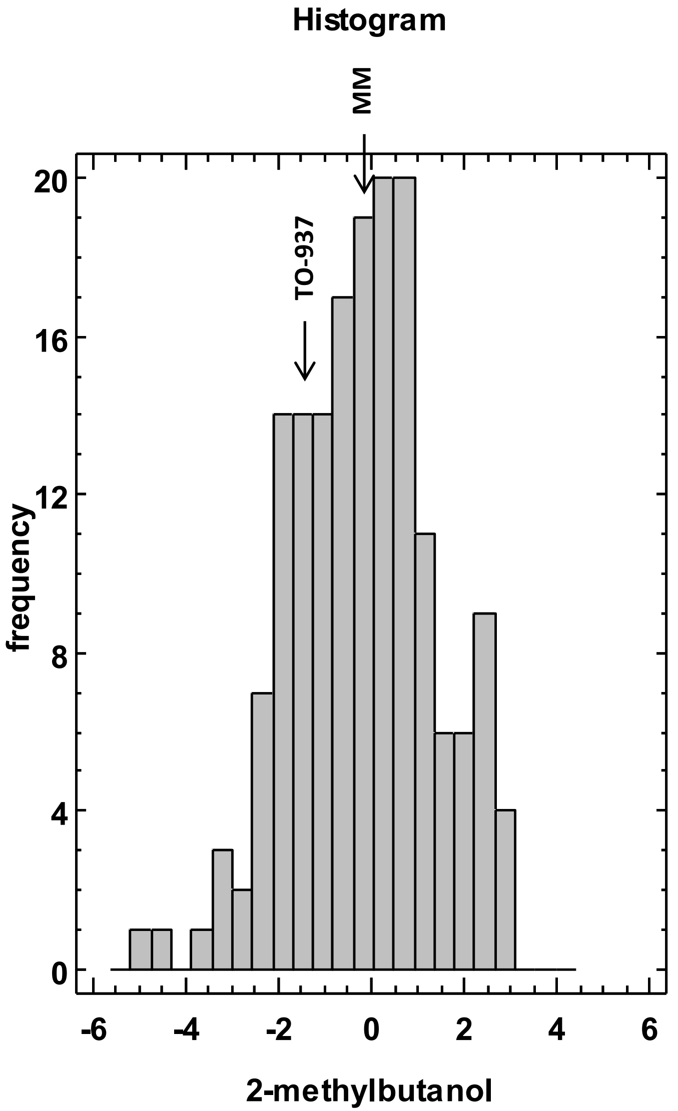

RILs season 2

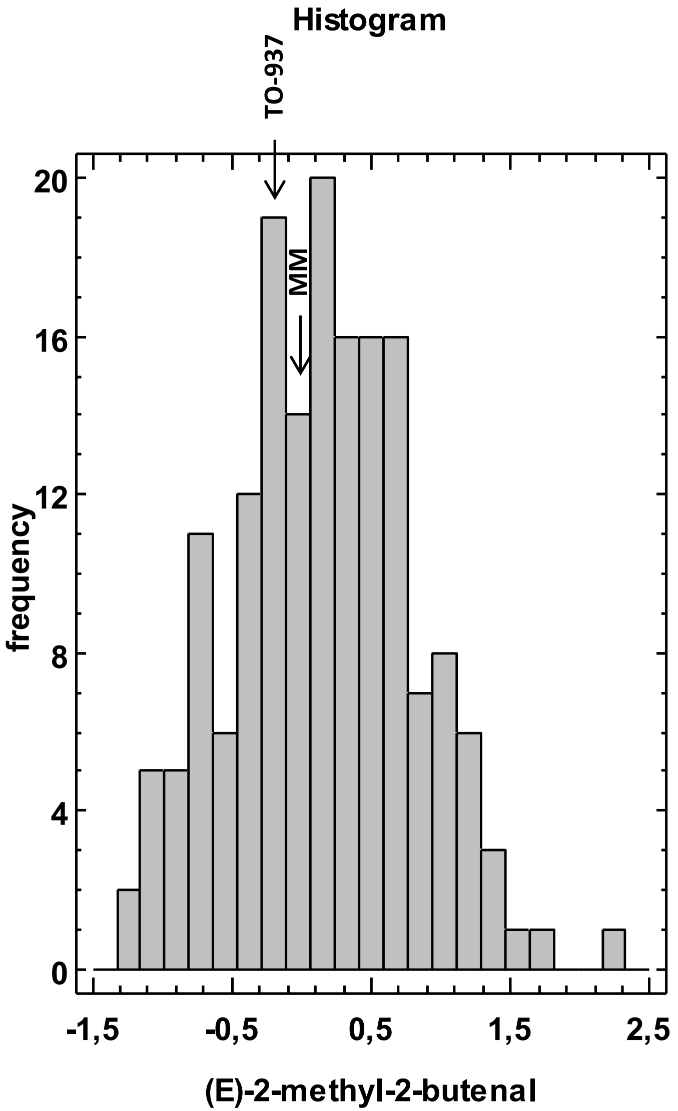

RILs season 2

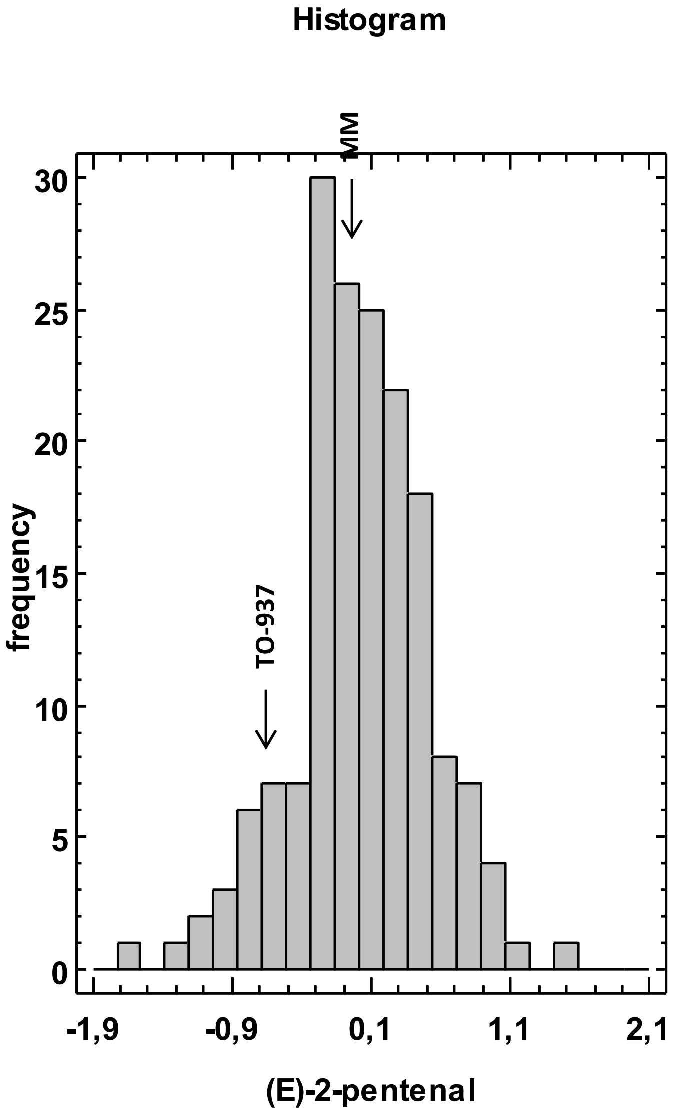

RILs season 2

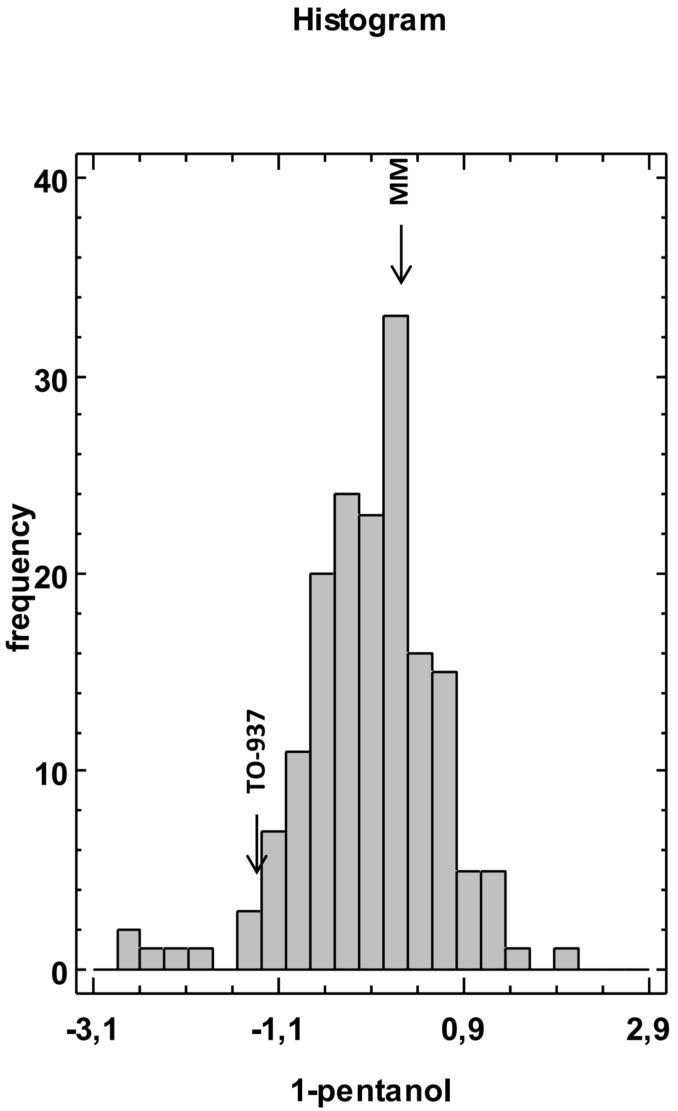

RILs season 2

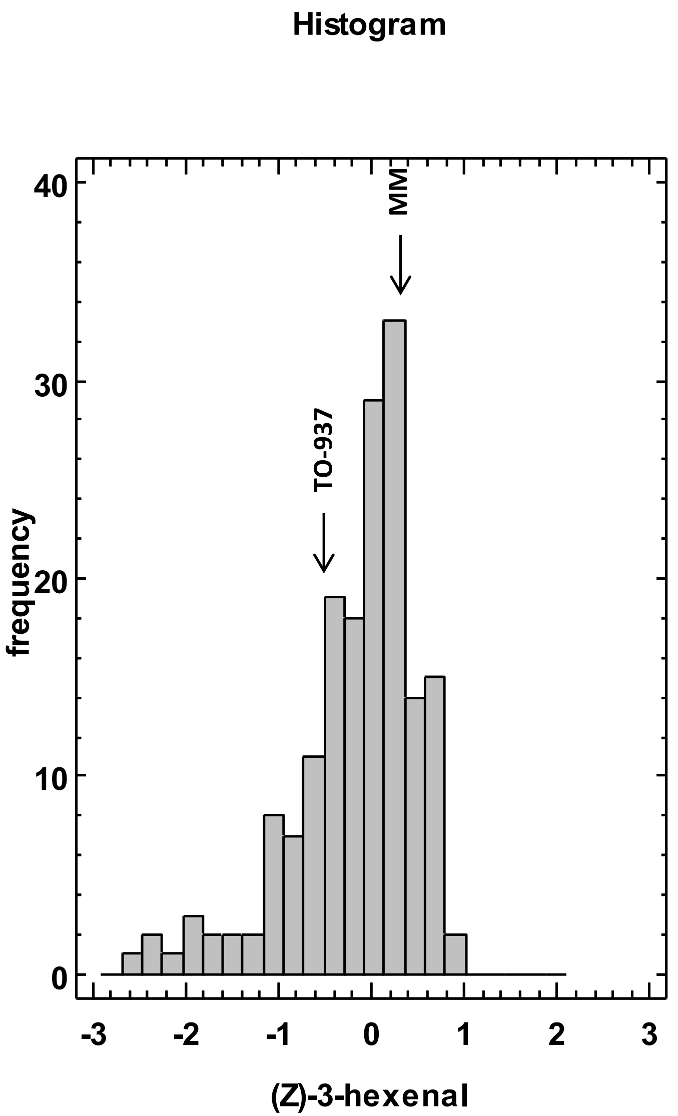

RILs season 2

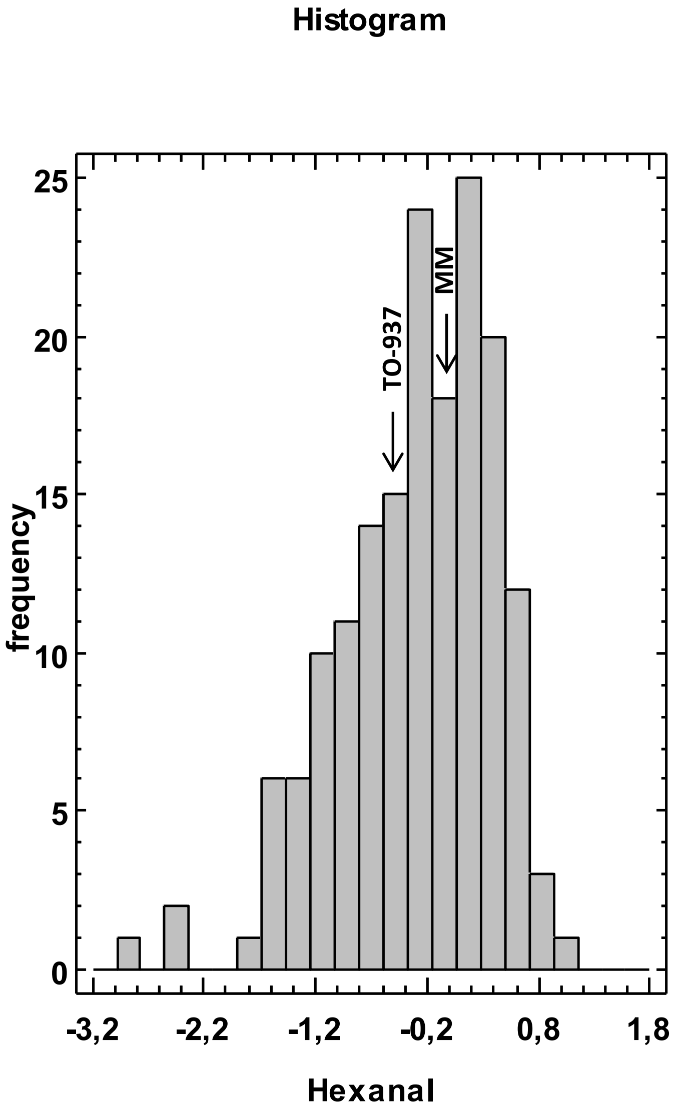

RILs season 2

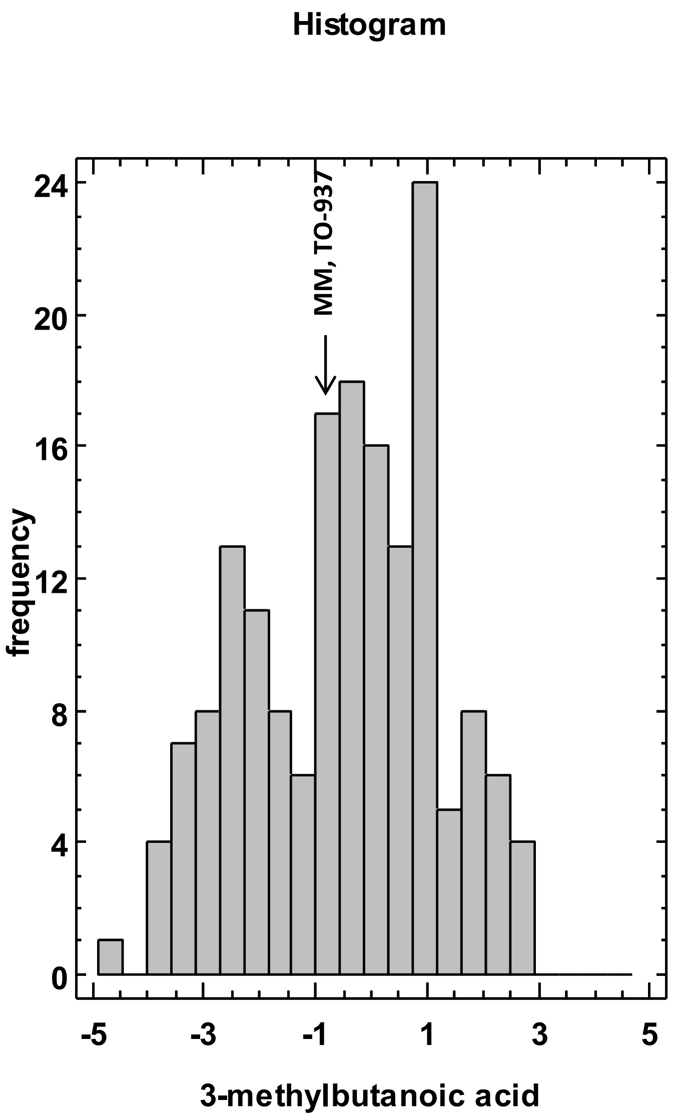

RILs season 2

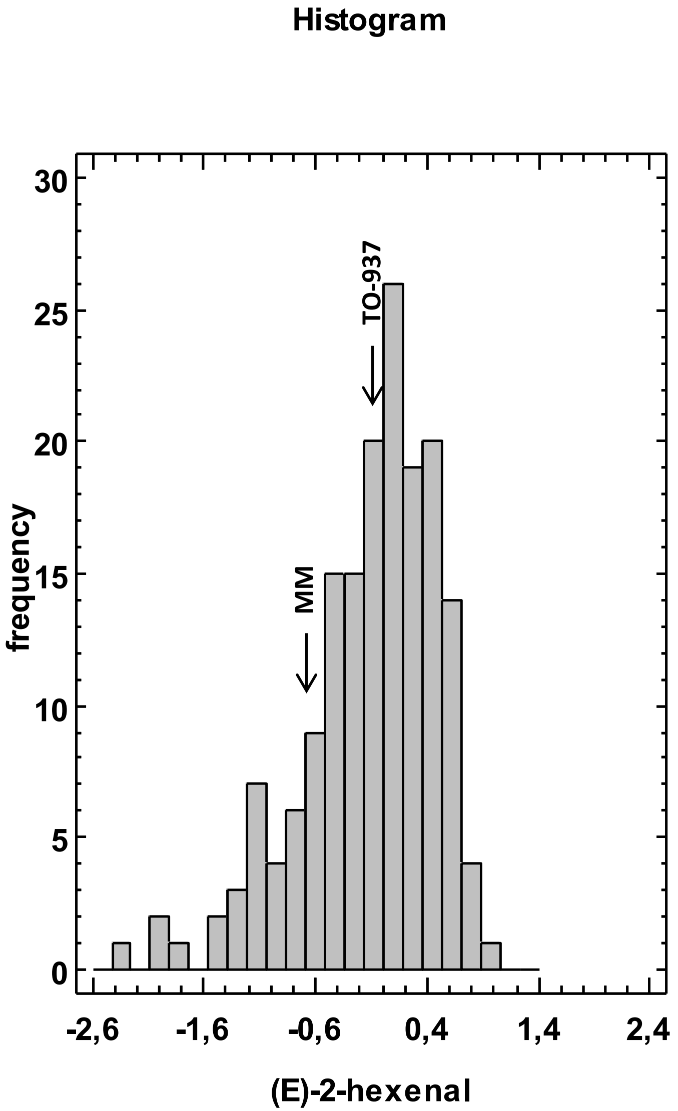

RILs season 2

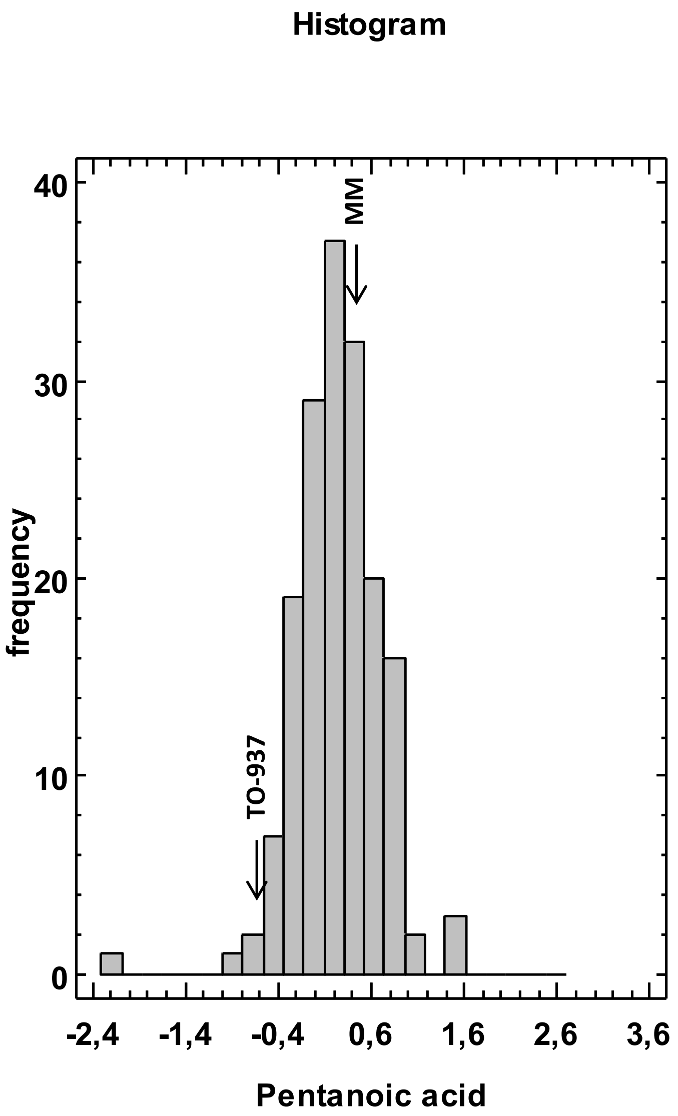

RILs season 2

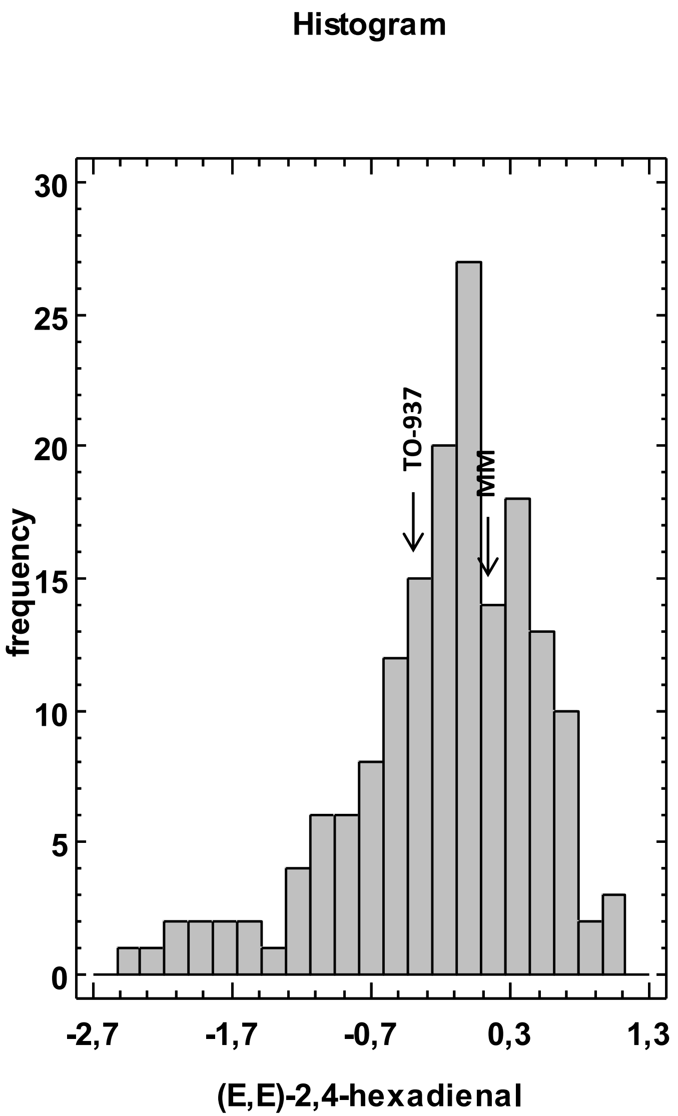

RILs season 2

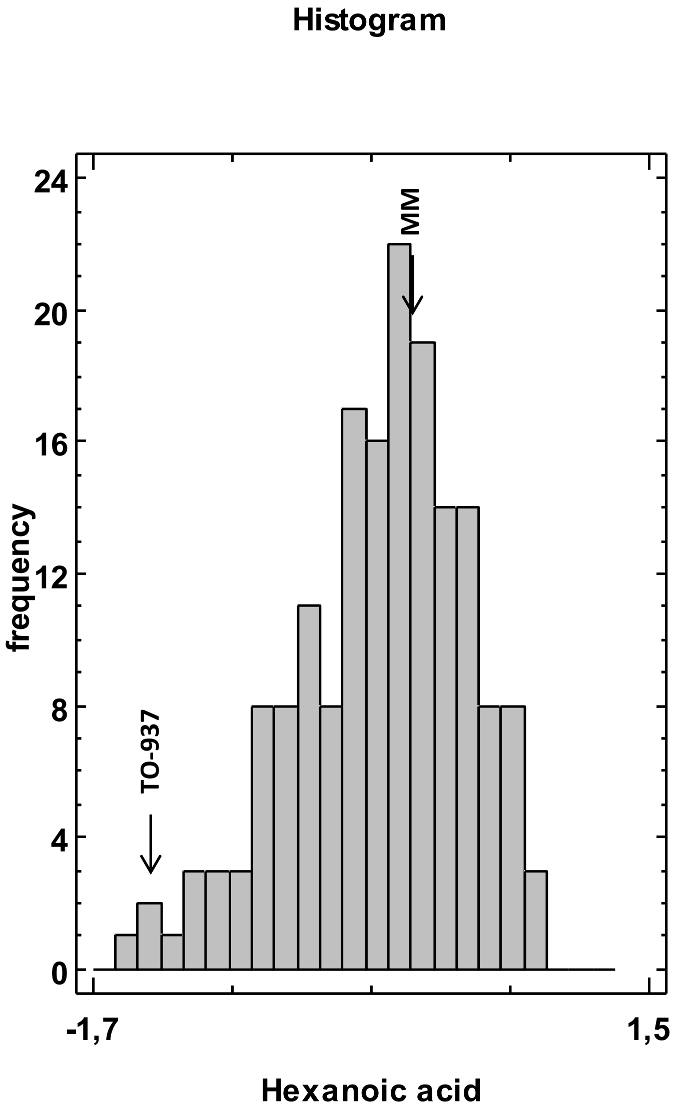

RILs season 2

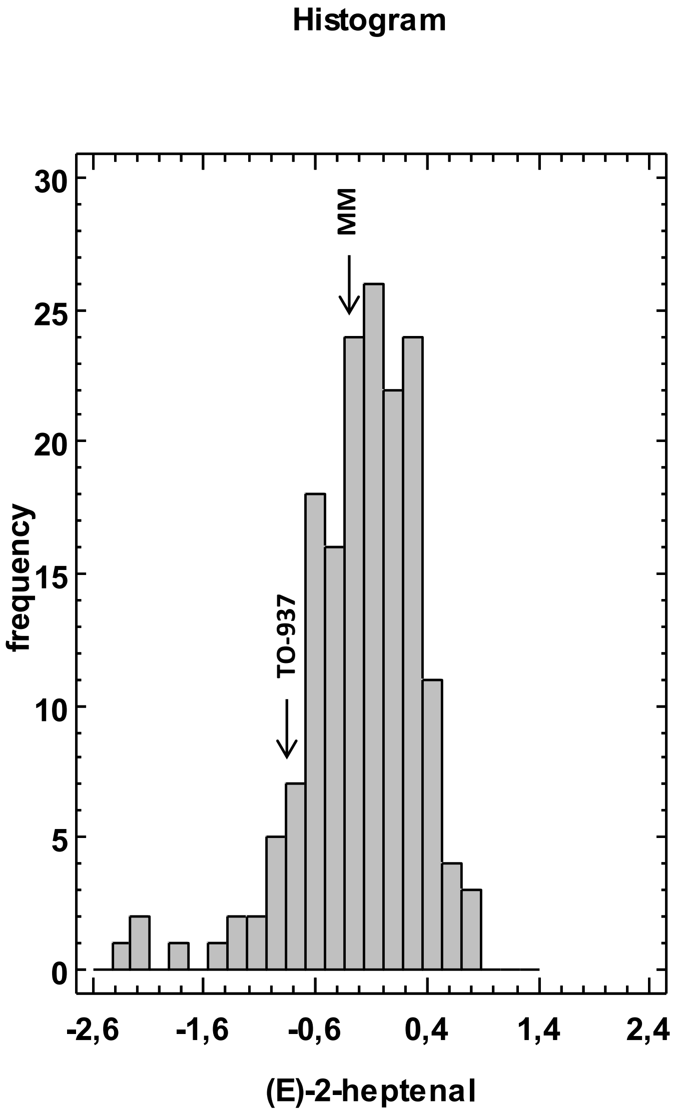

RILs season 2

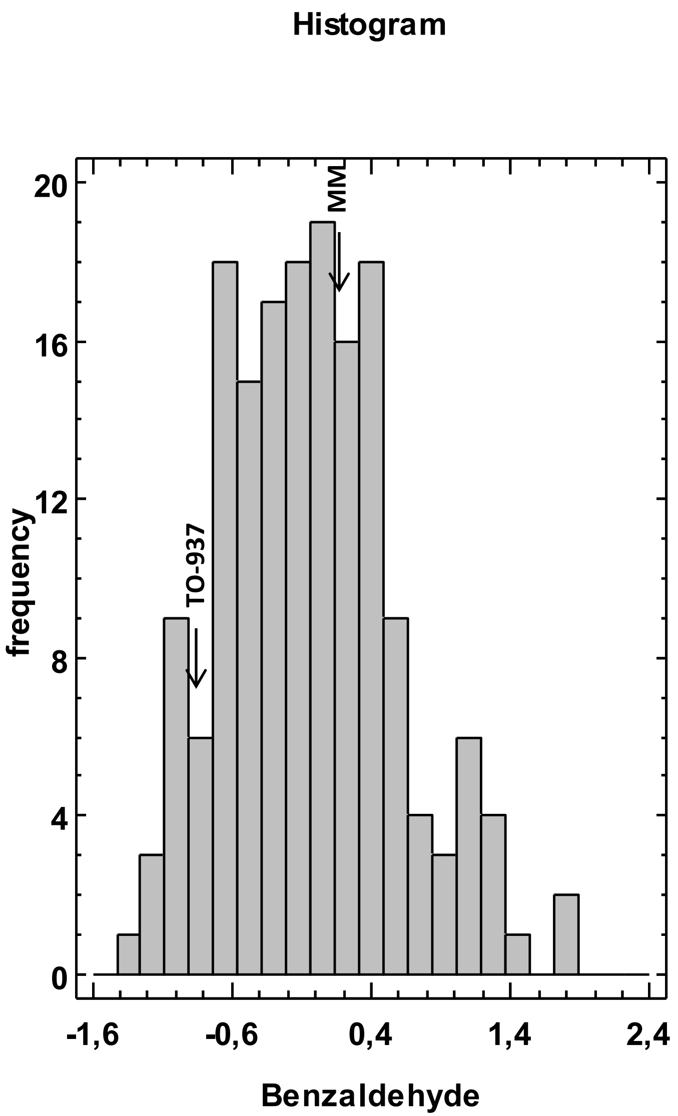

RILs season 2

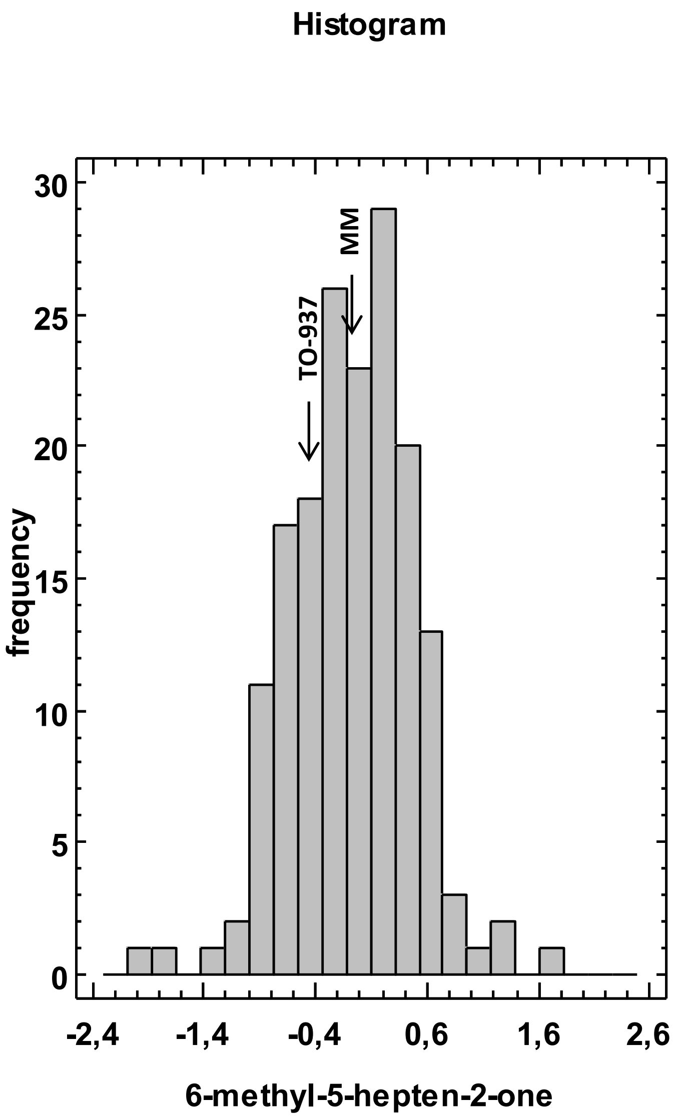

RILs season 2

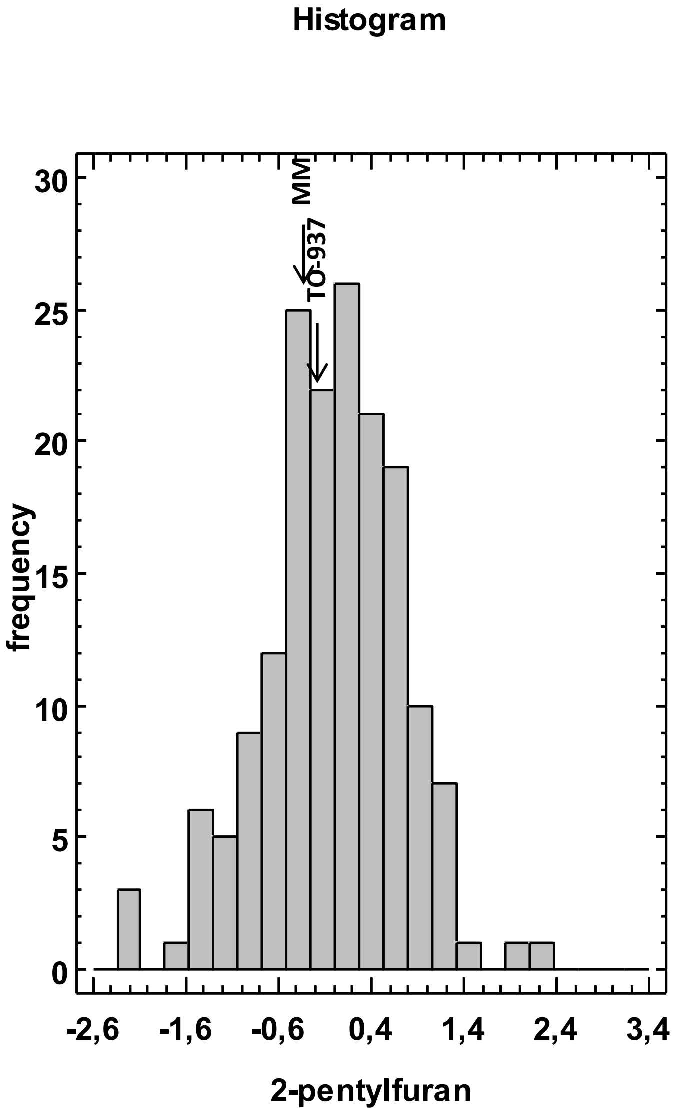

RILs season 2

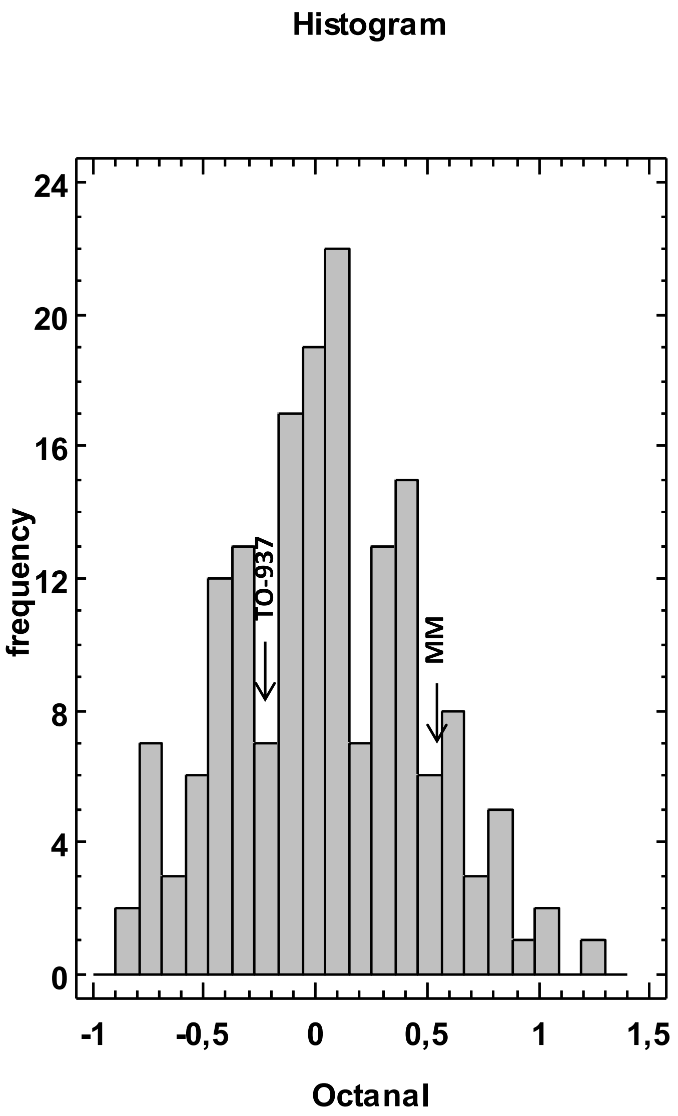

RILs season 2

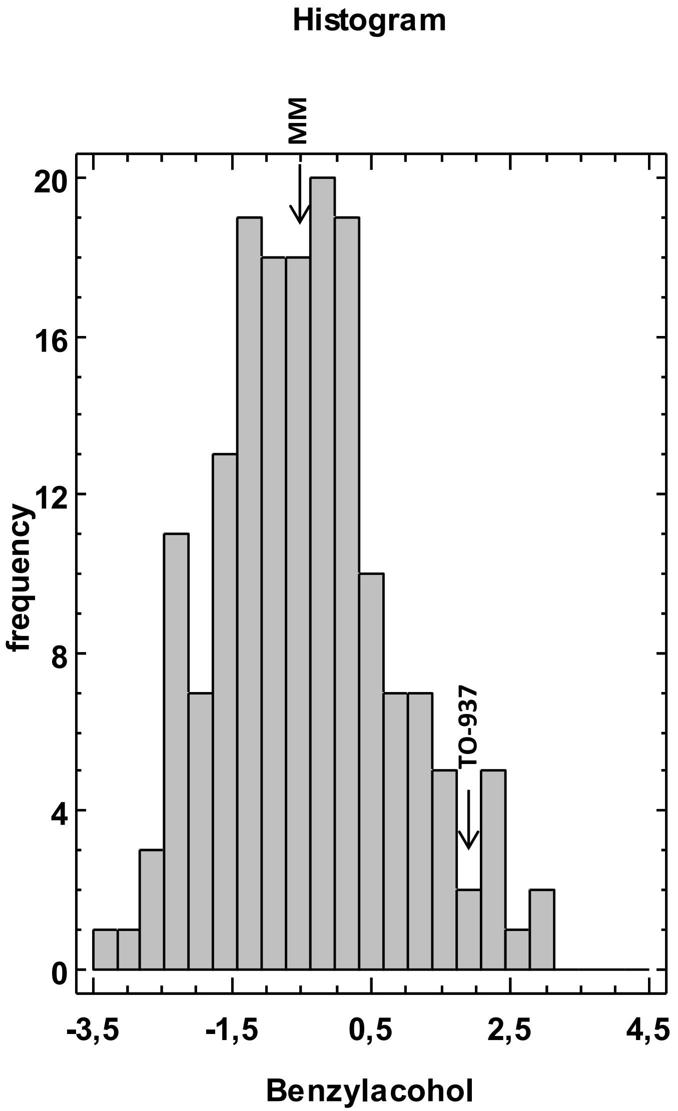

RILs season 2

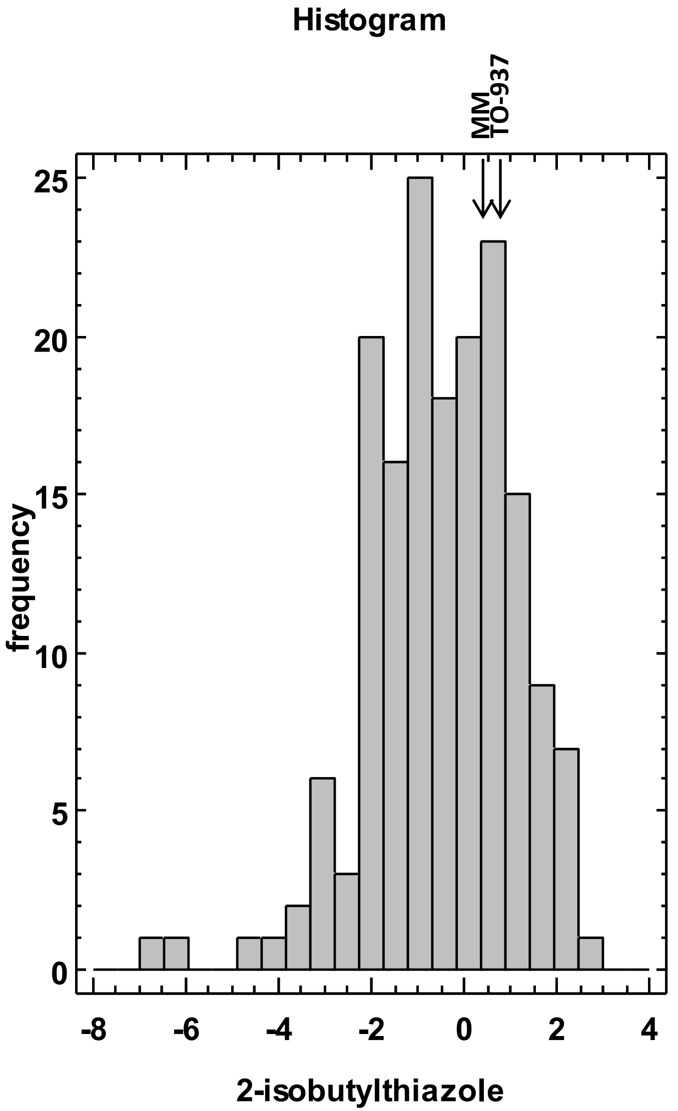

RILs season 2

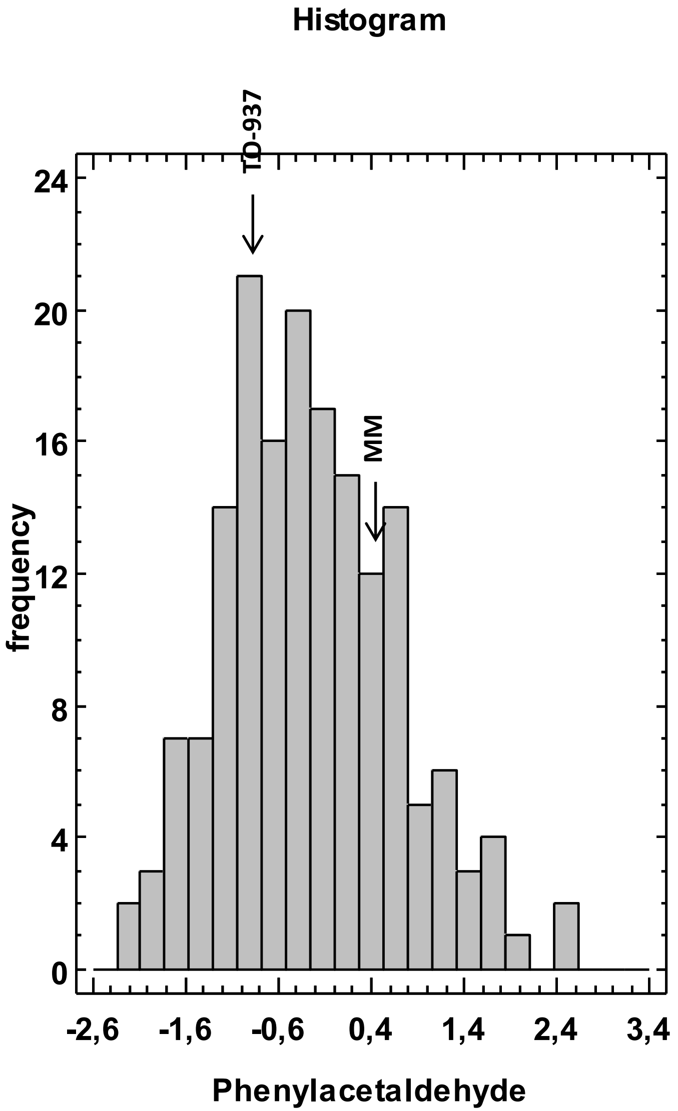

RILs season 2

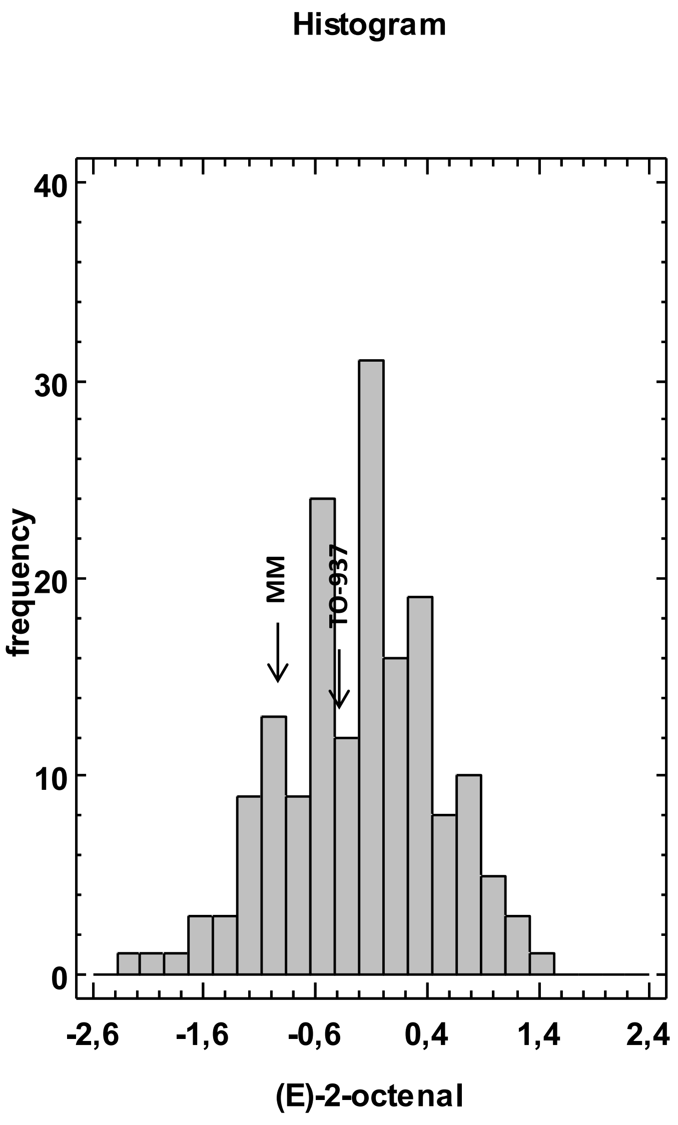

RILs season 2

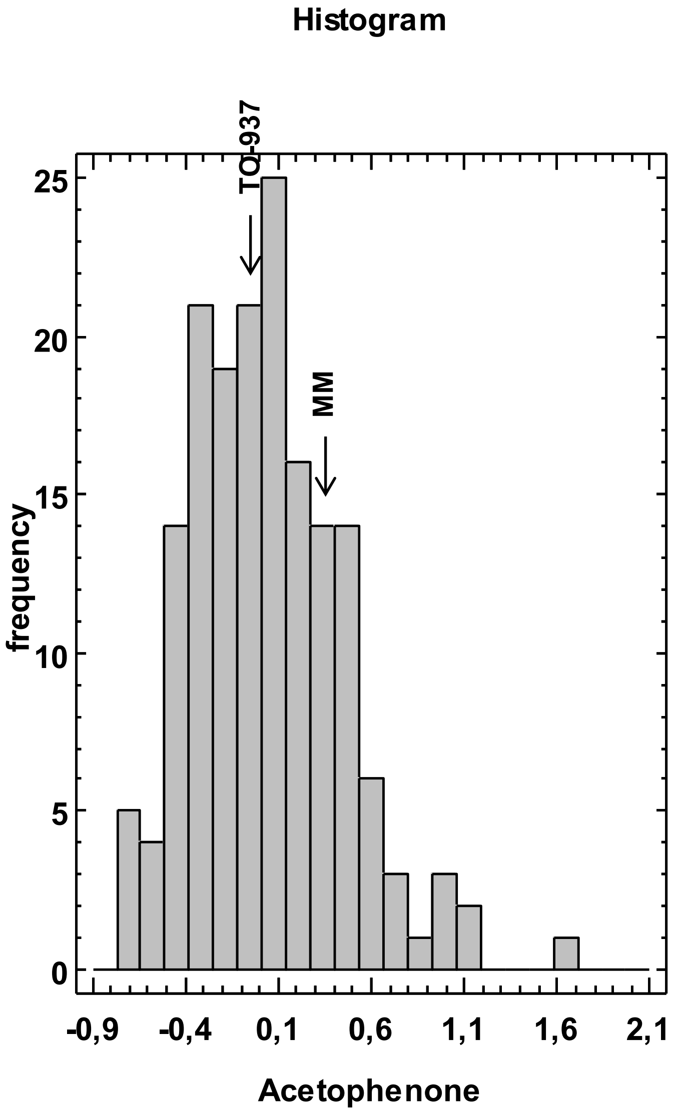

RILs season 2

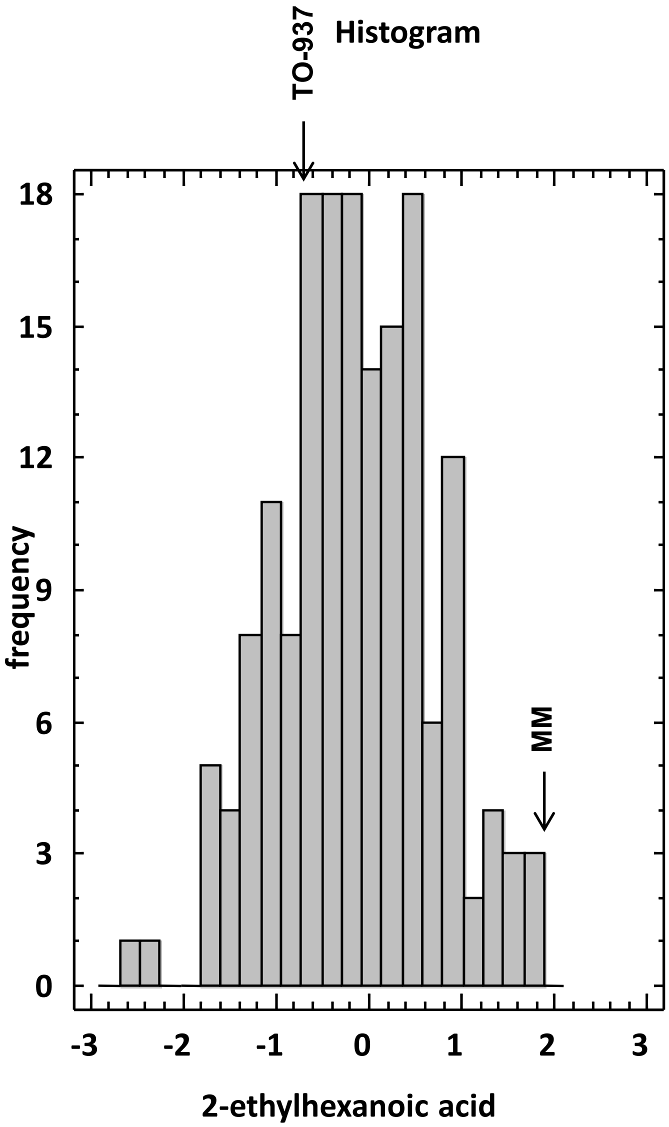

RILs season 2

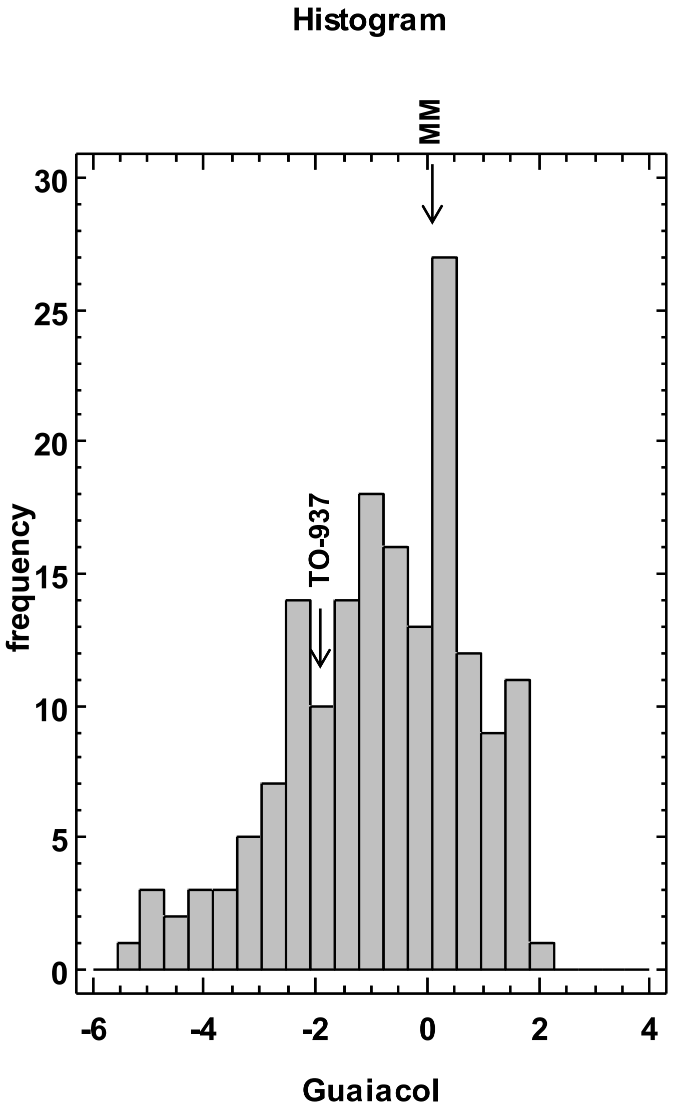

RILs season 2

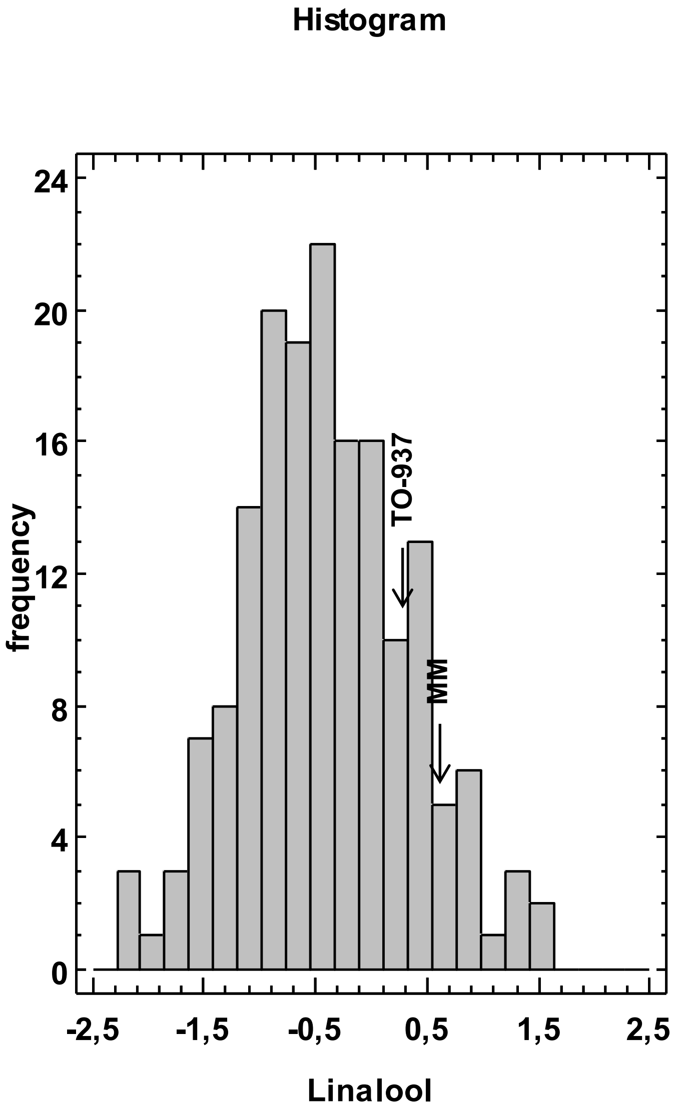

RILs season 2

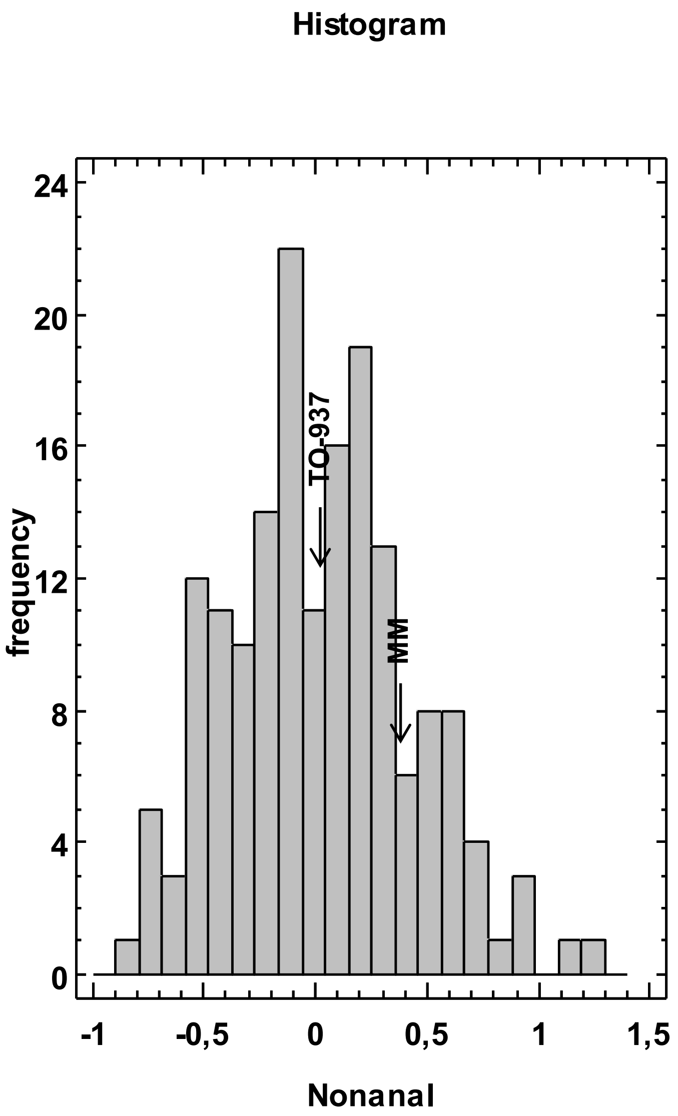

RILs season 2

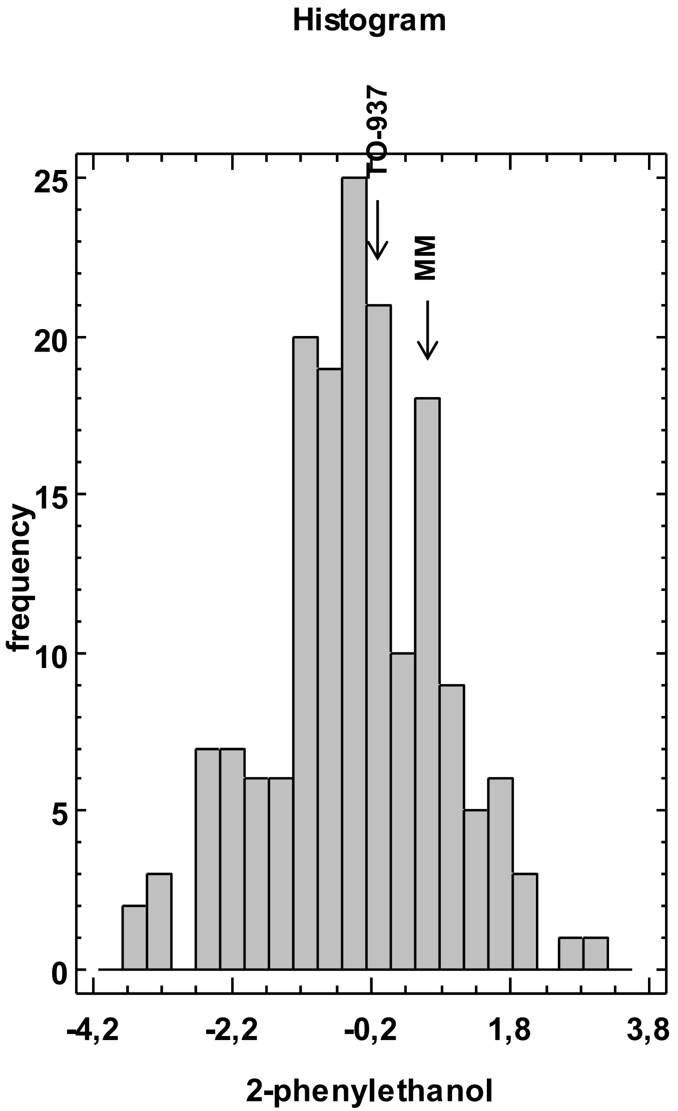

RILs season 2

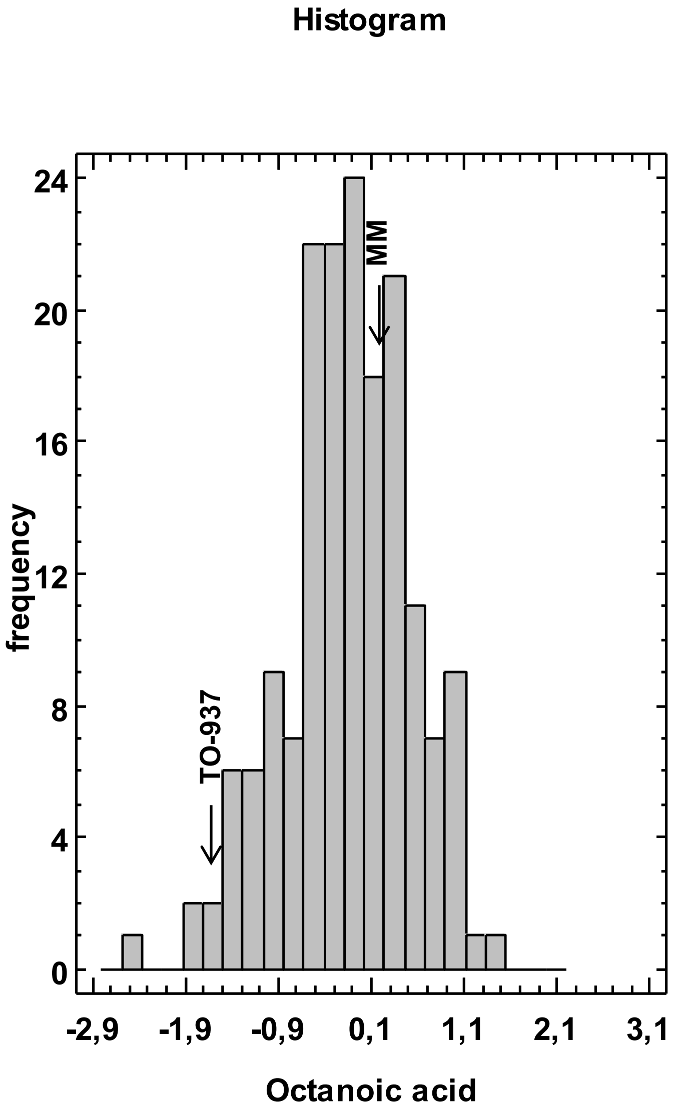

RILs season 2

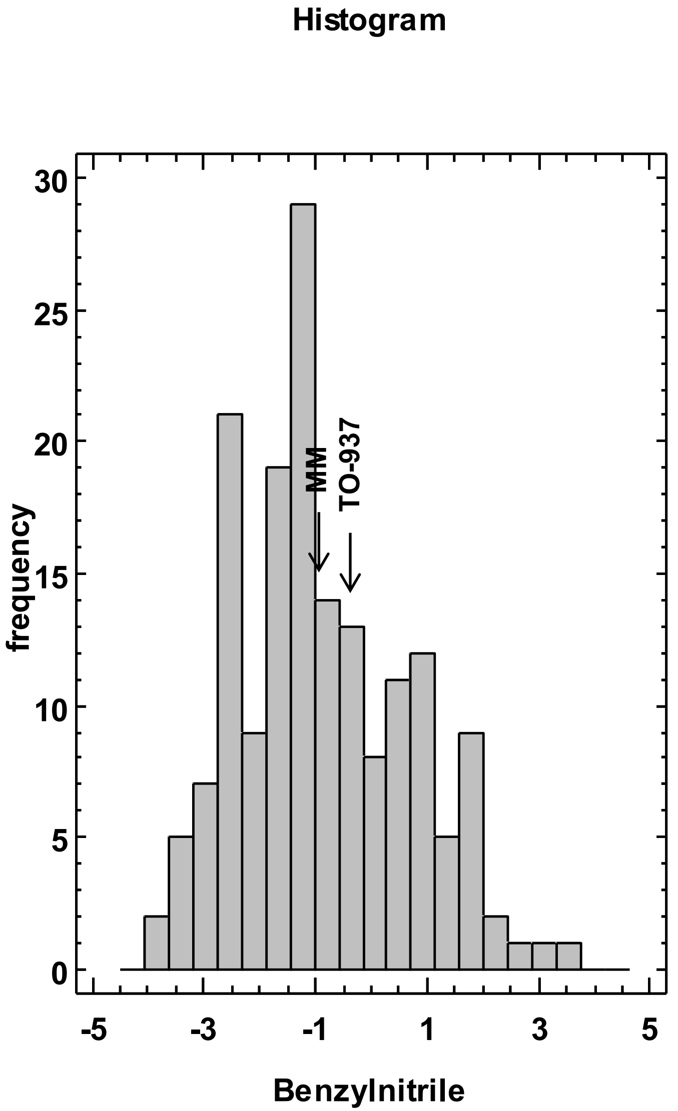

RILs season 2

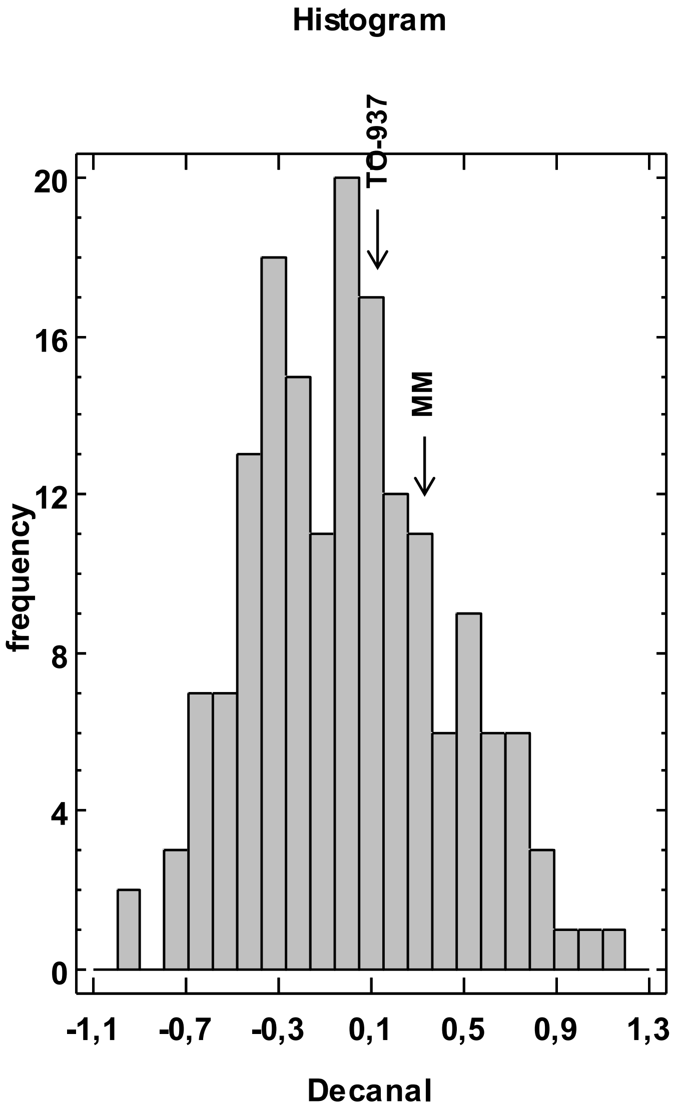

RILs season 2

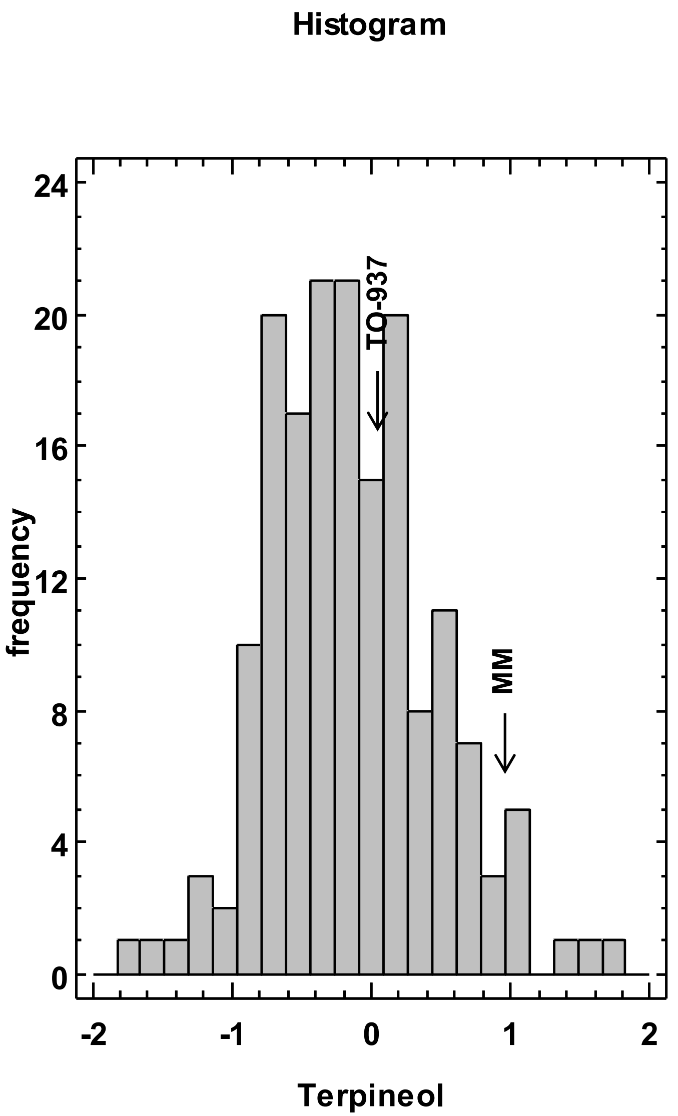

RILs season 2

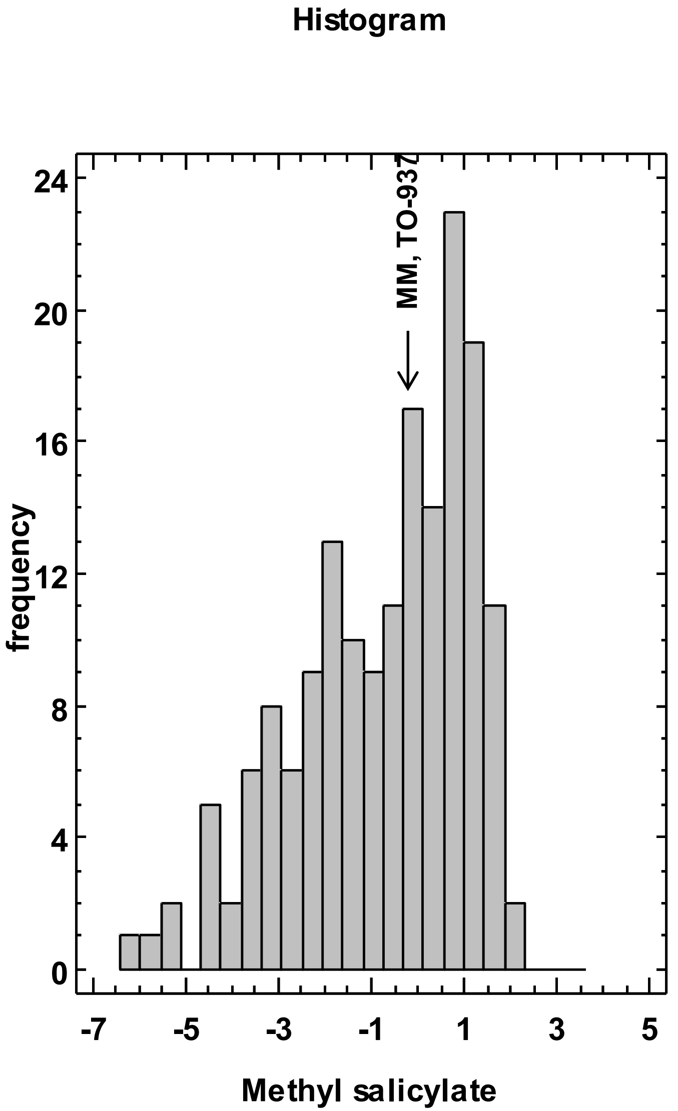

RILs season 2

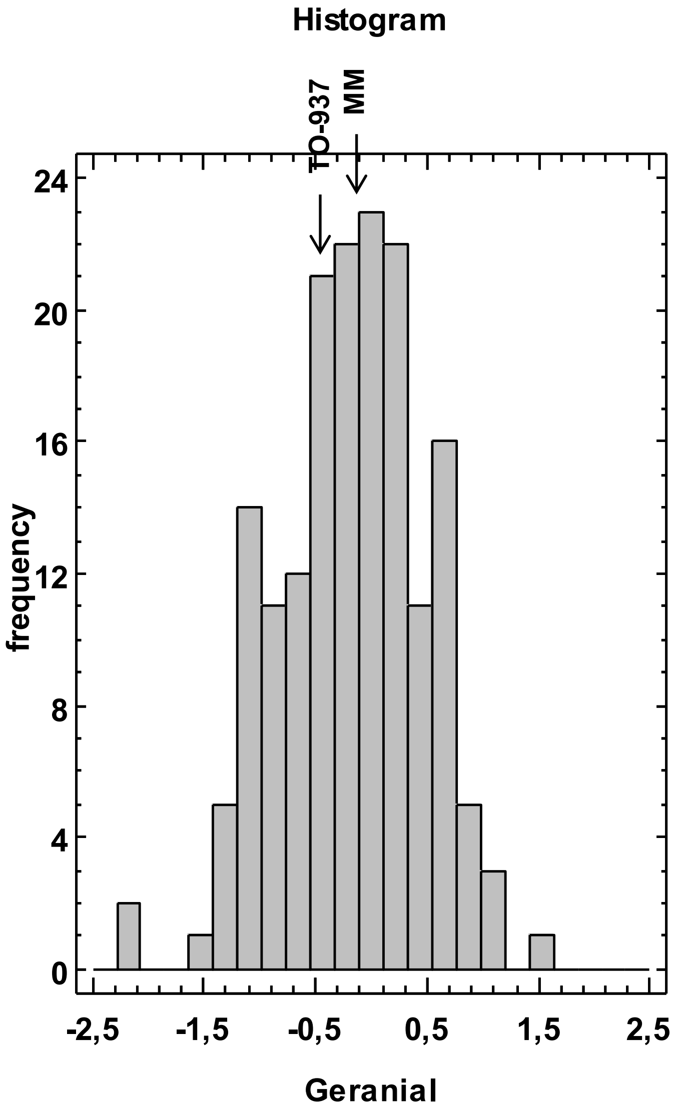

RILs season 2

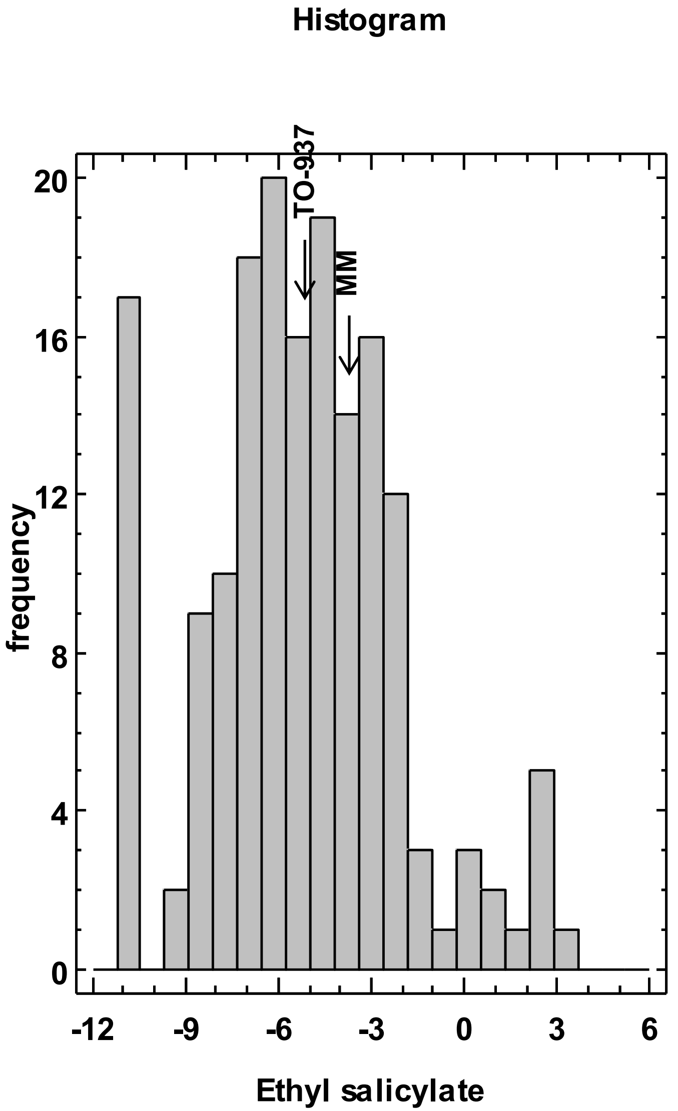

RILs season 2

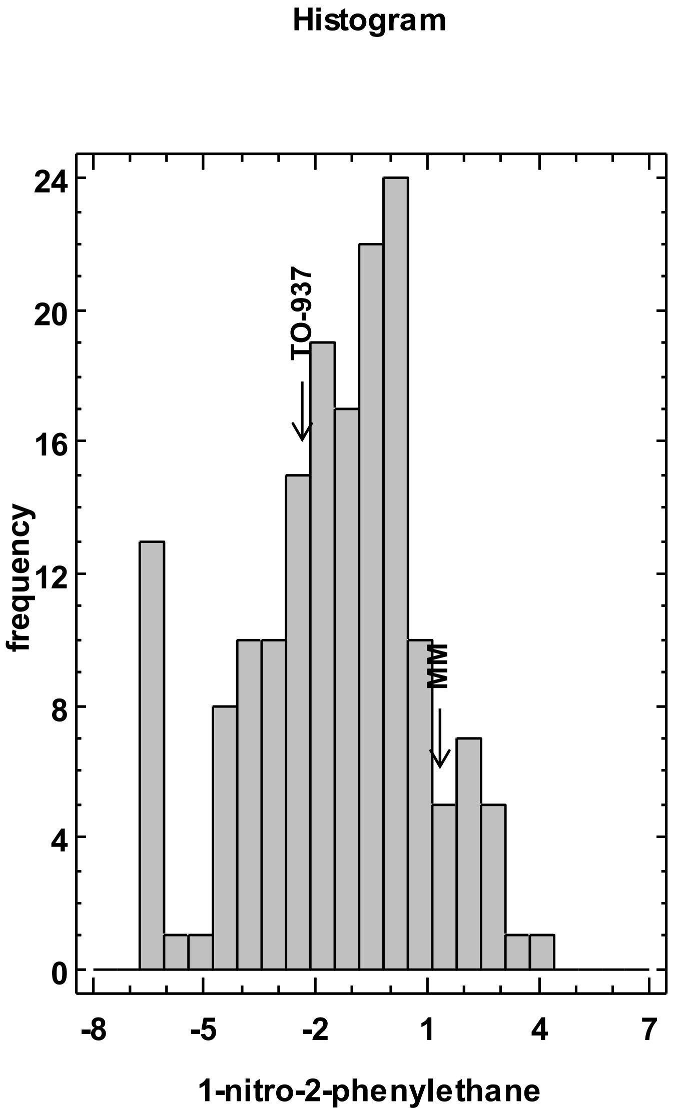

RILs season 2

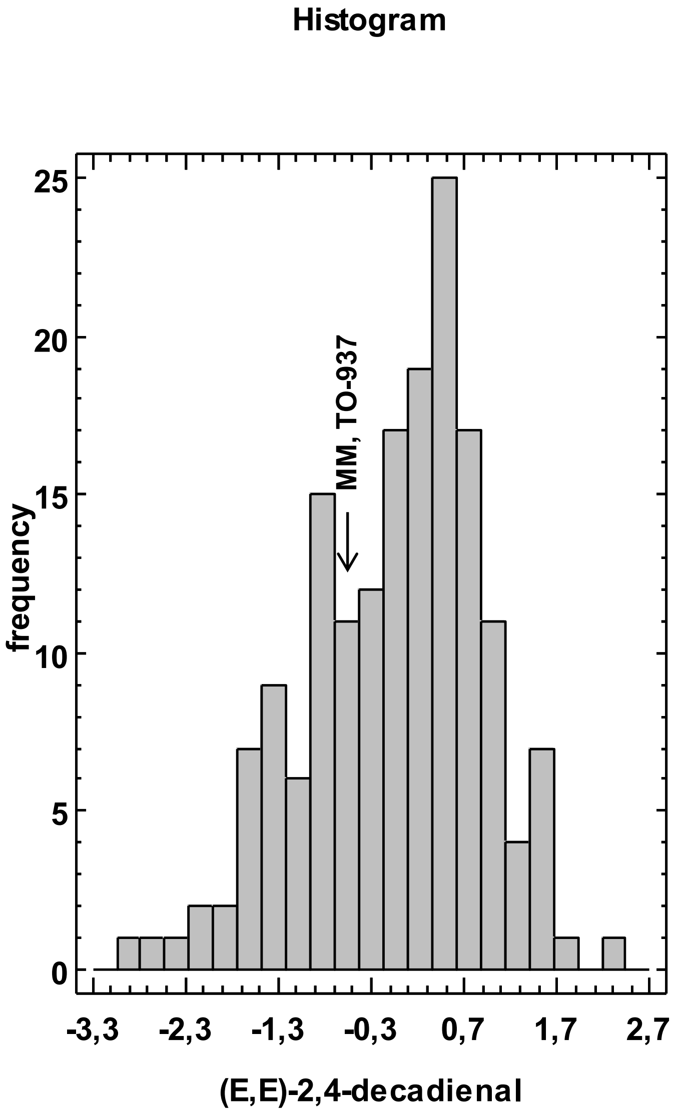

RILs season 2

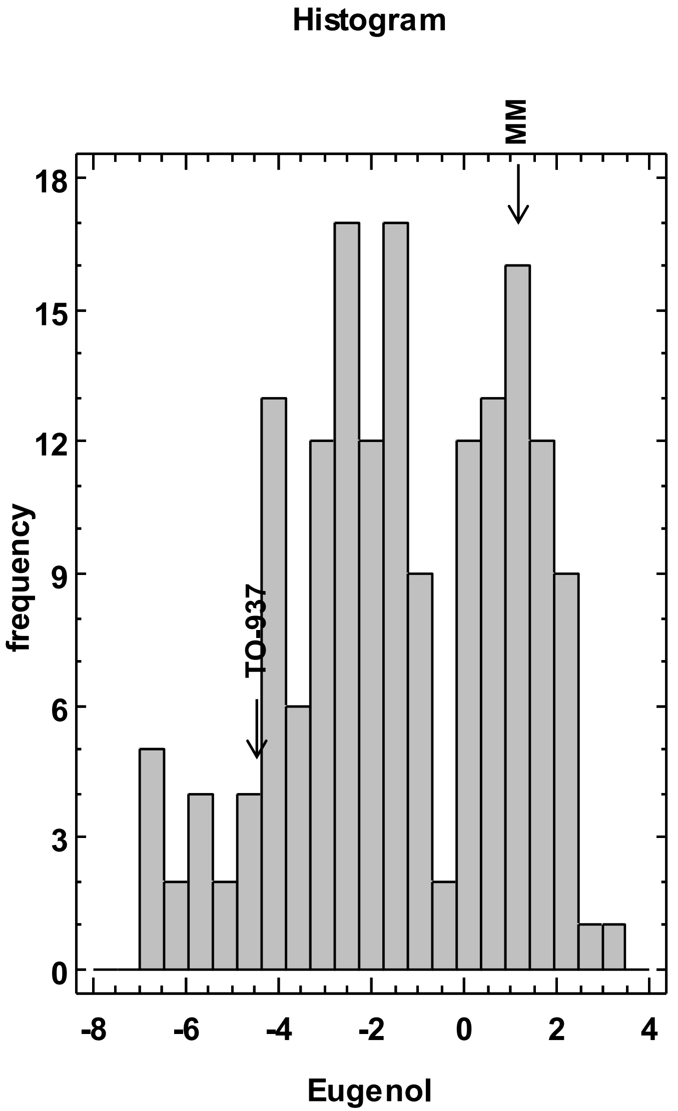

RILs season 2

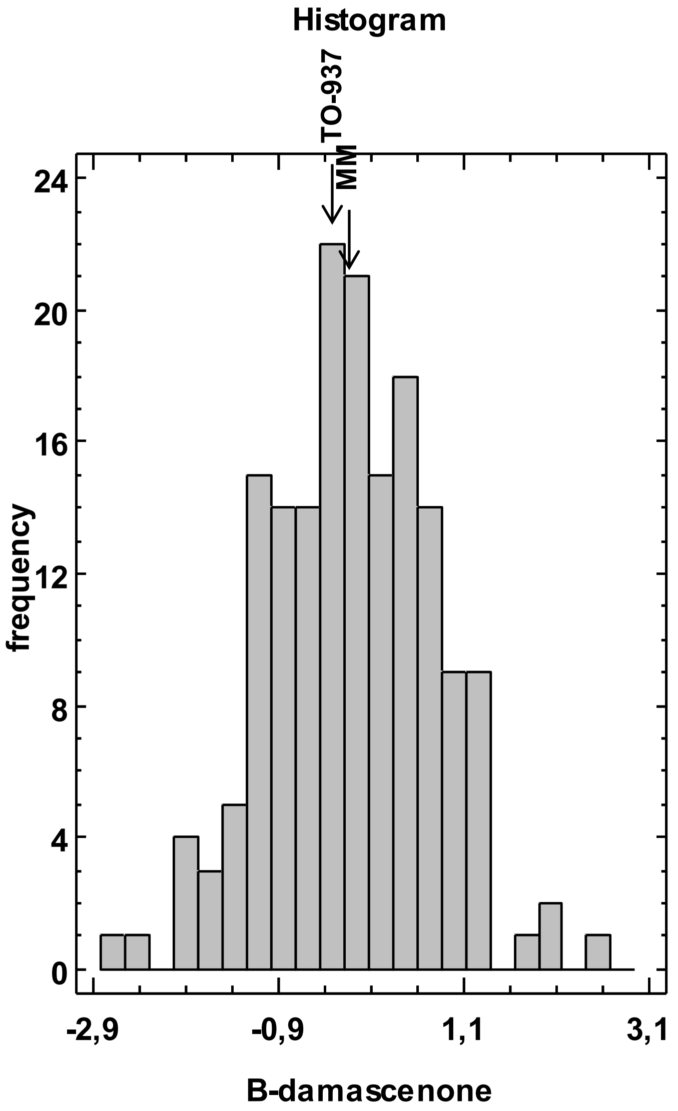

RILs season 2

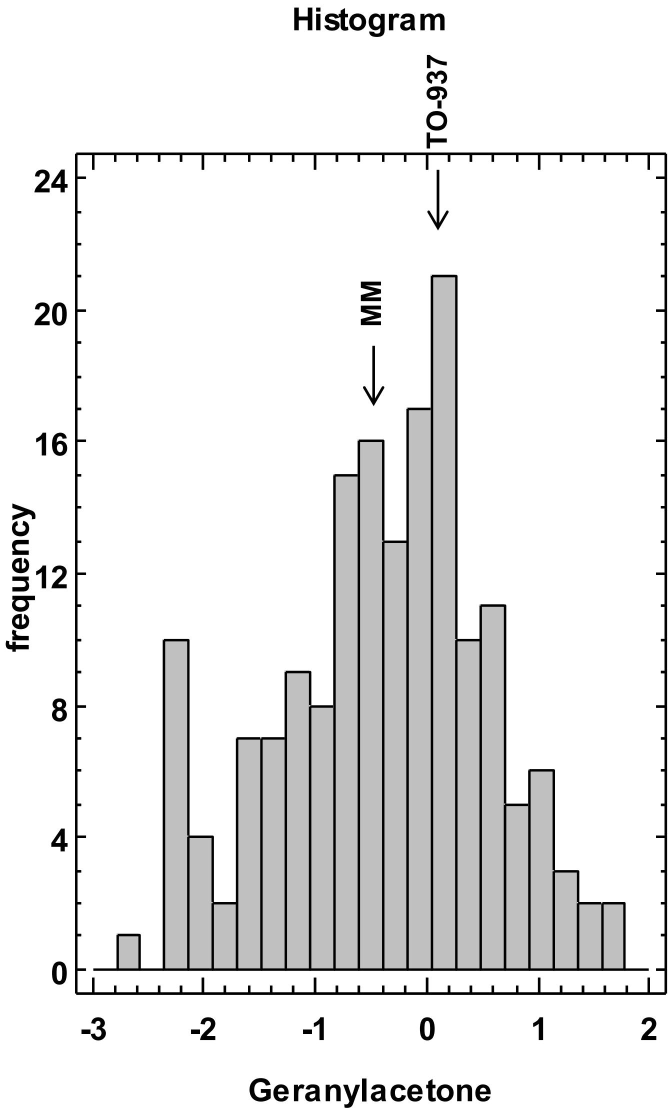

RILs season 2

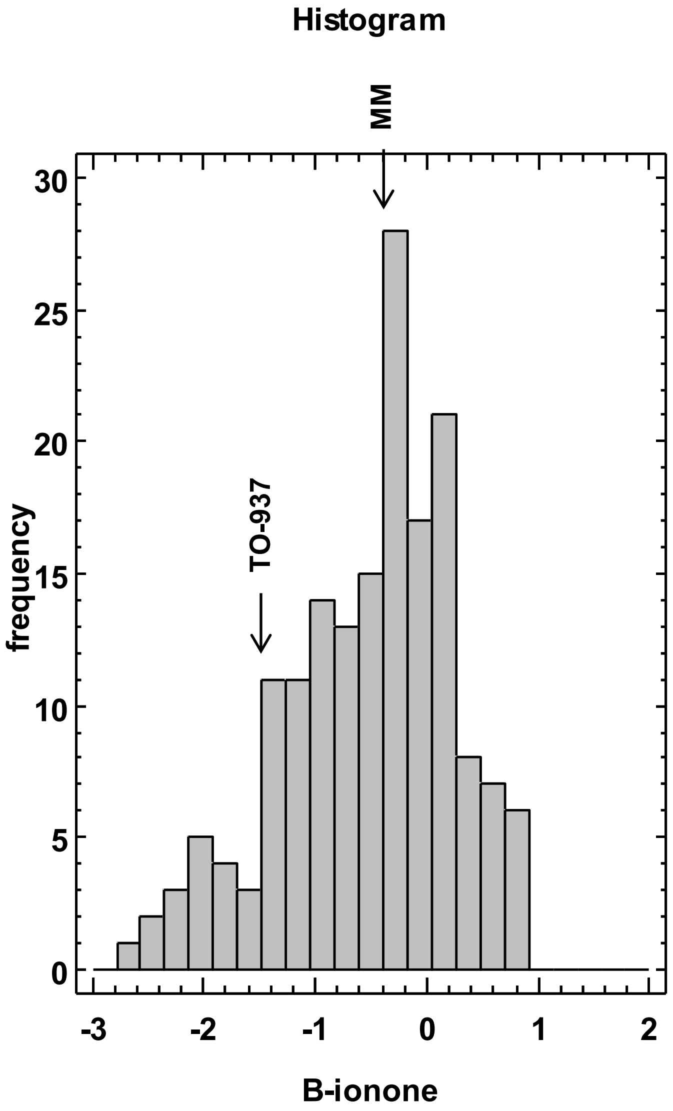

RILs season 2

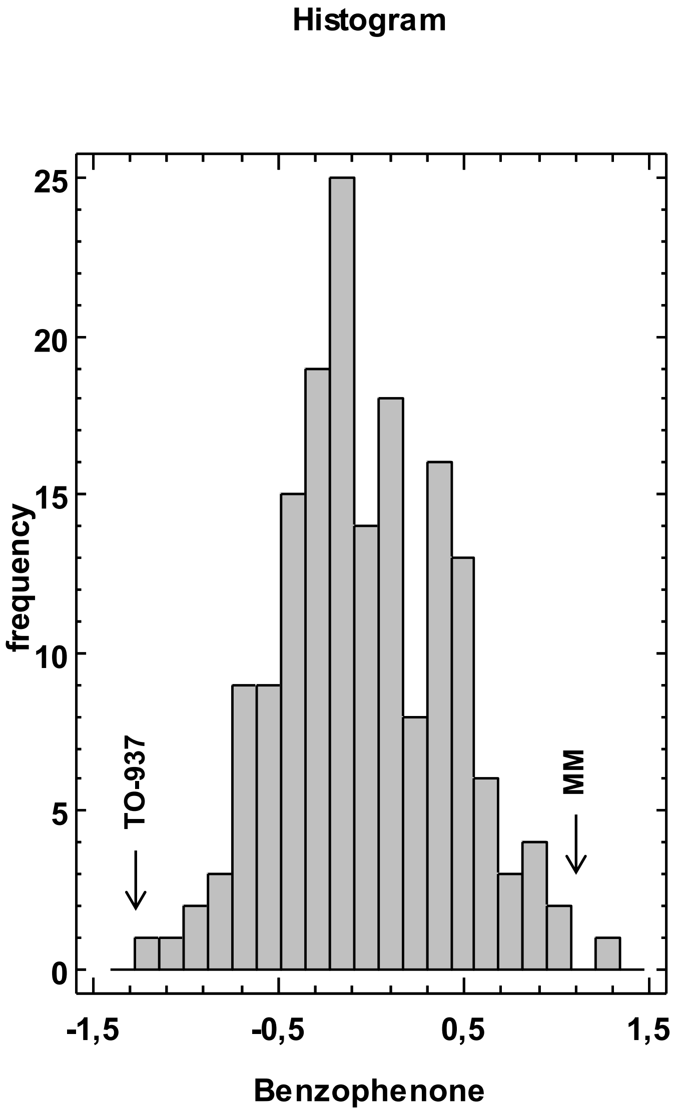

RILs season 3

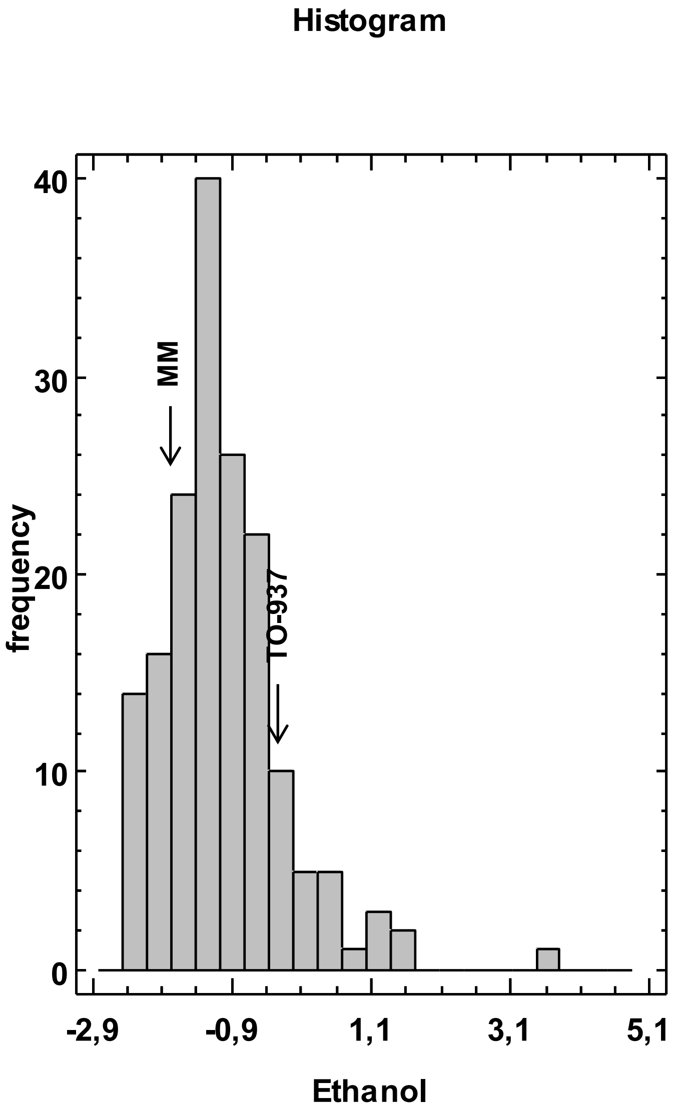

RILs season 3

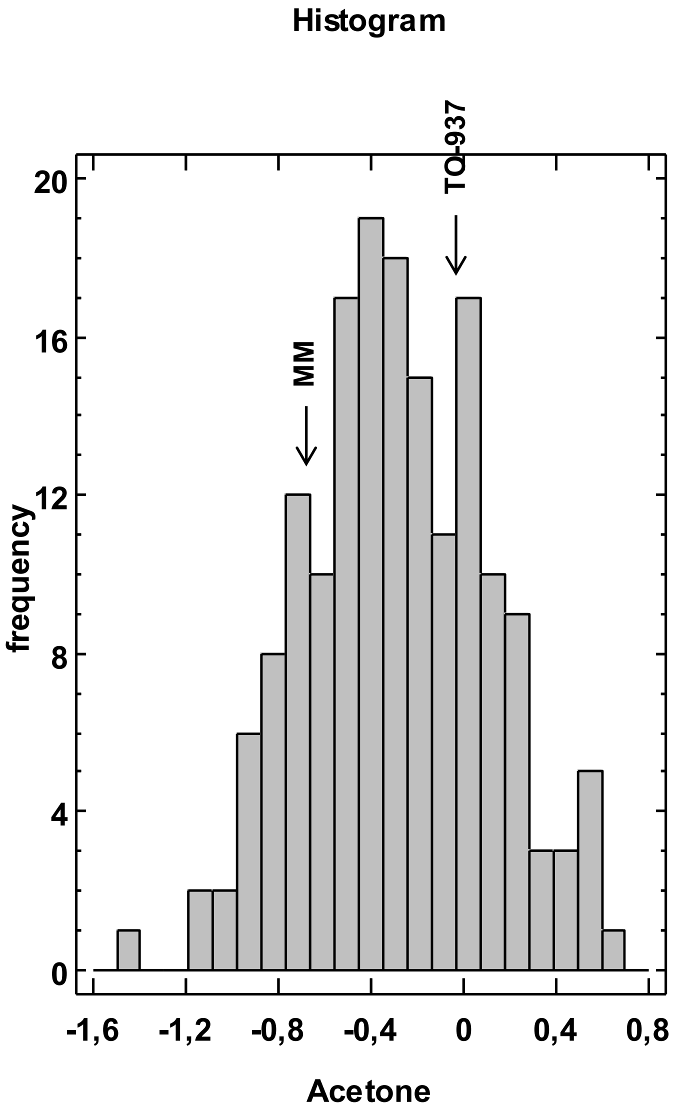

RILs season 3

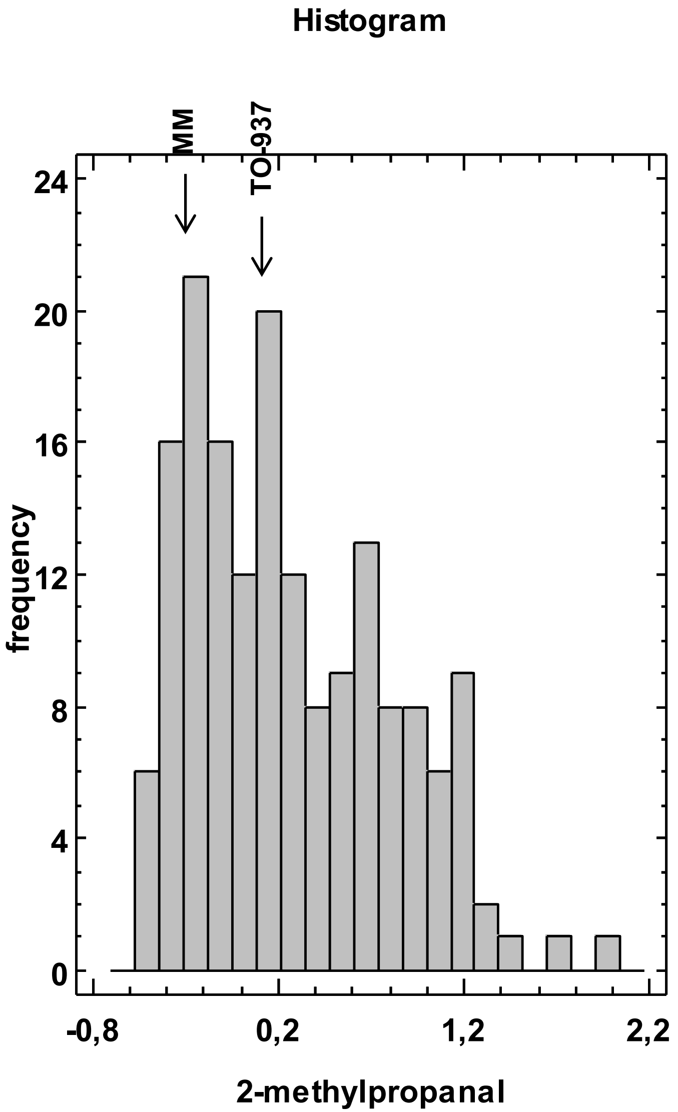

RILs season 3

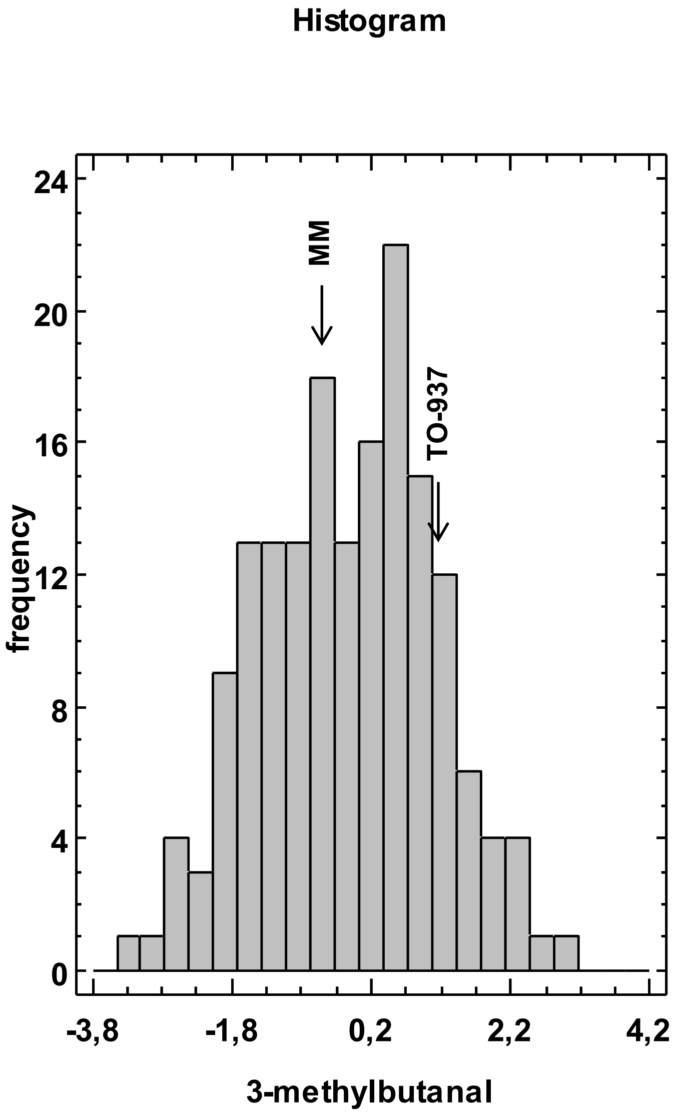

RILs season 3

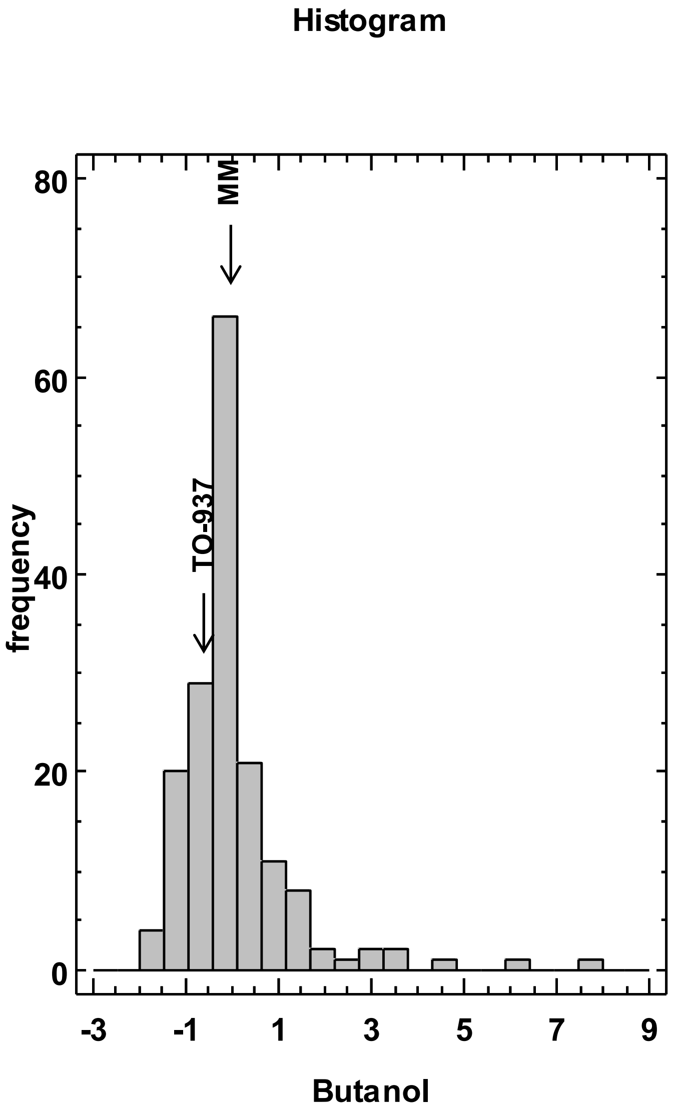

RILs season 3

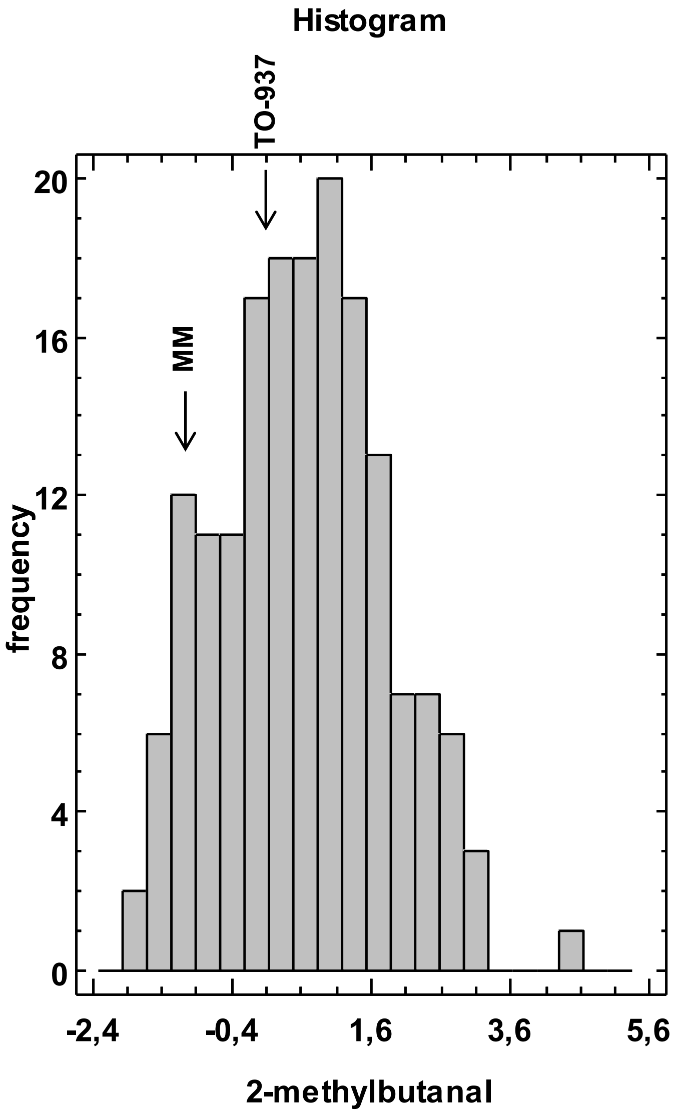

RILs season 3

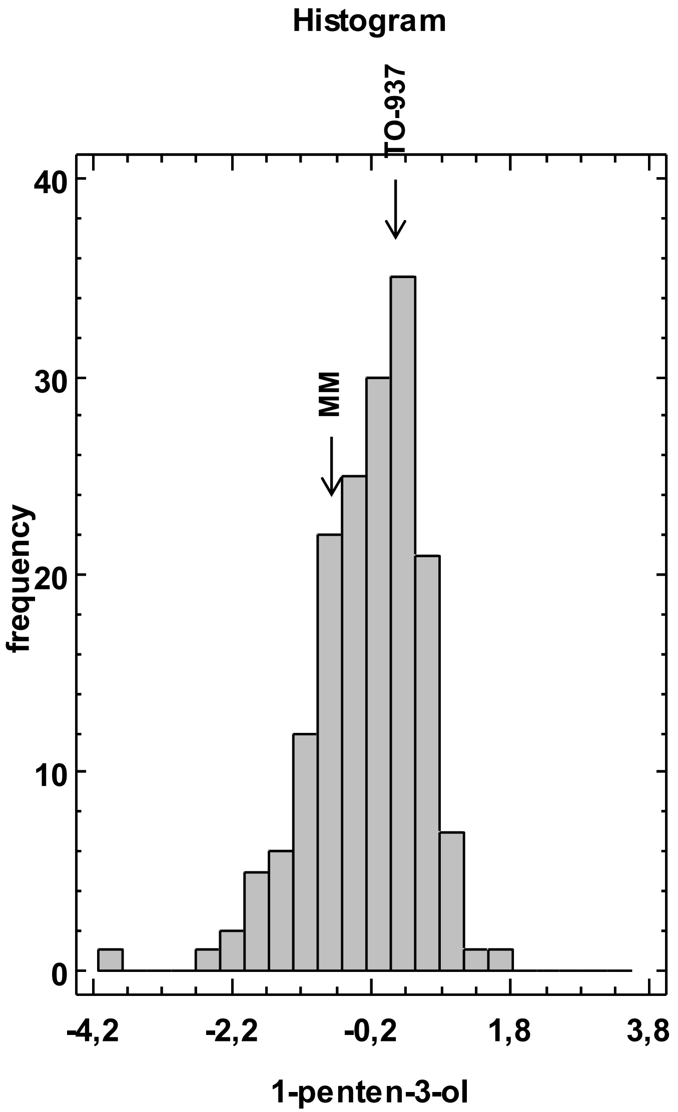

RILs season 3

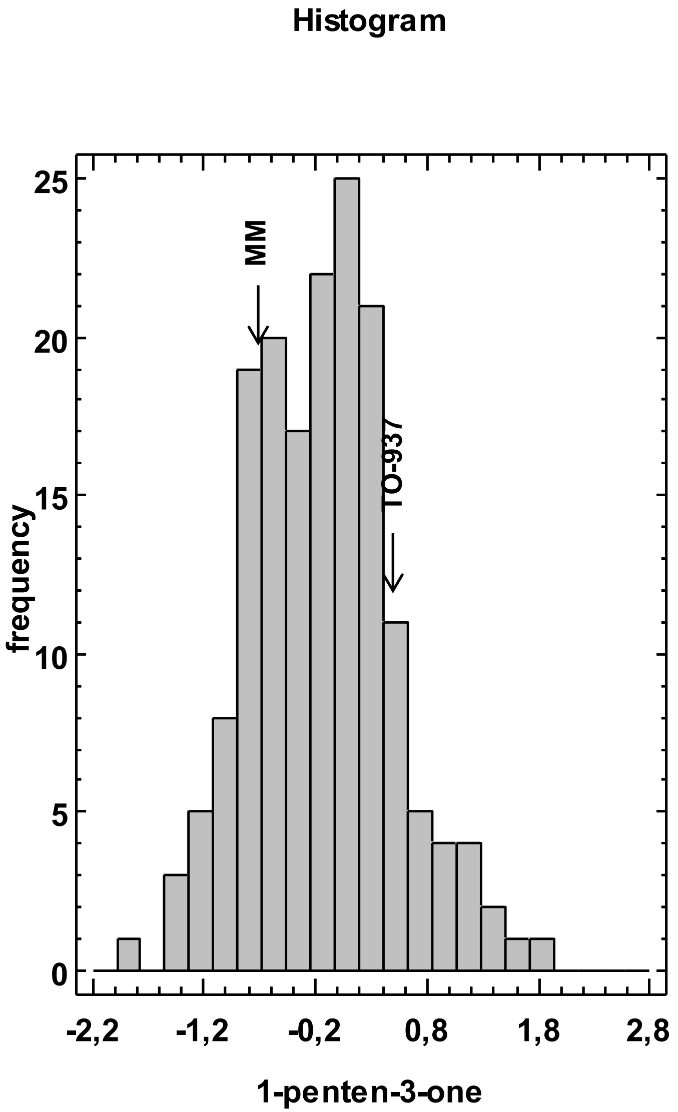

RILs season 3

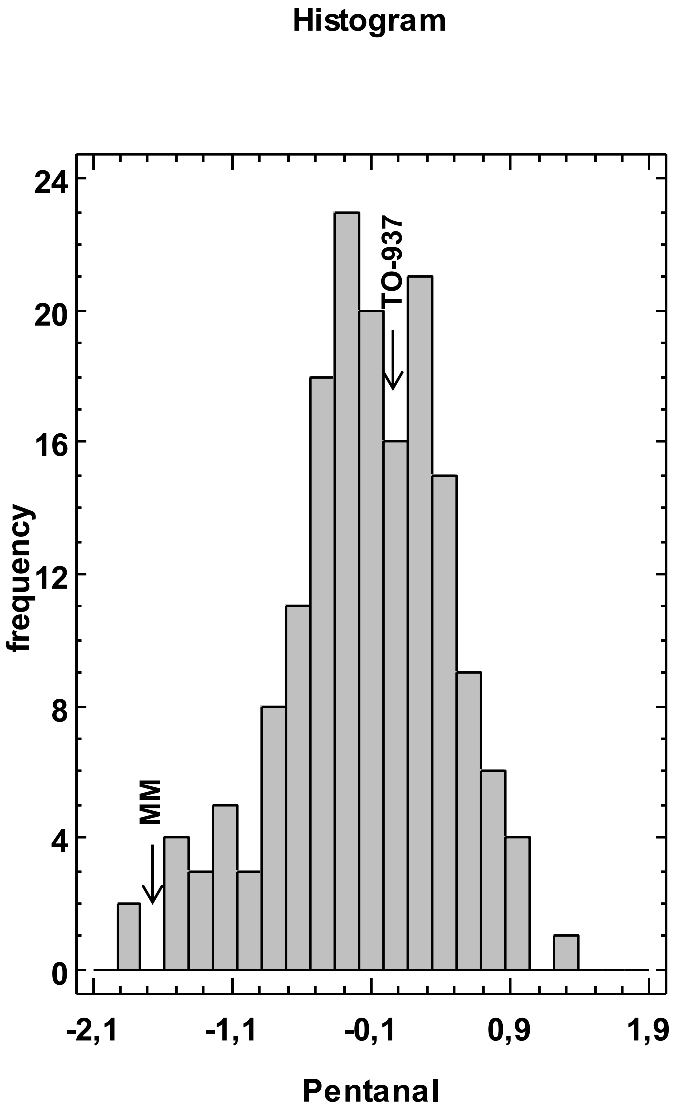

RILs season 3

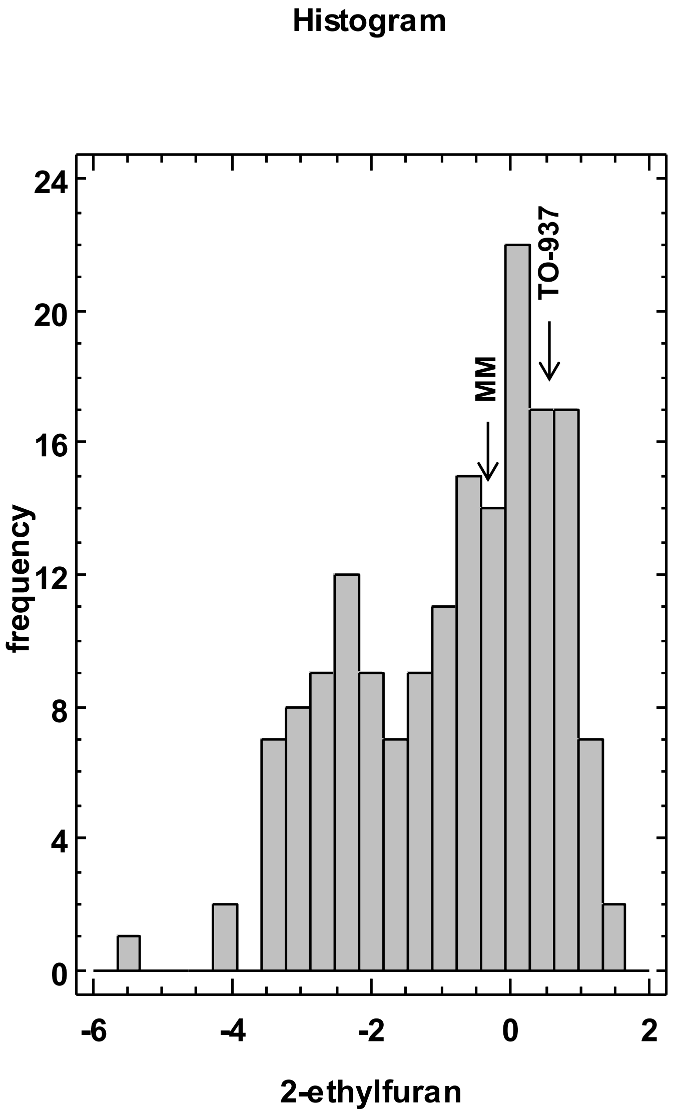

RILs season 3

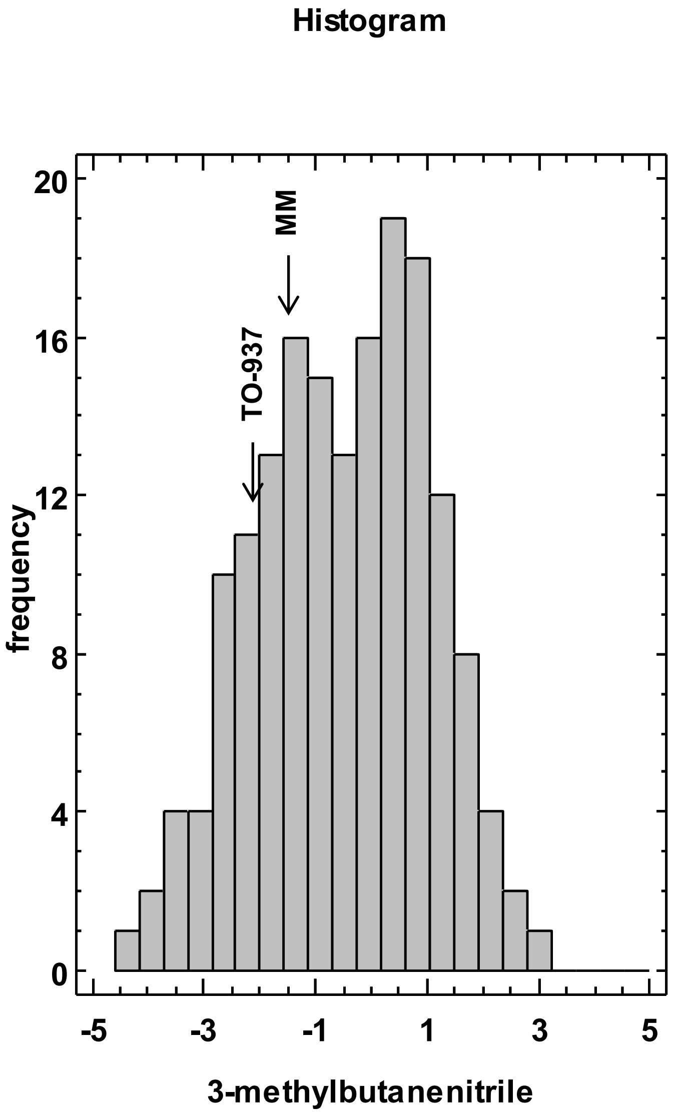

RILs season 3

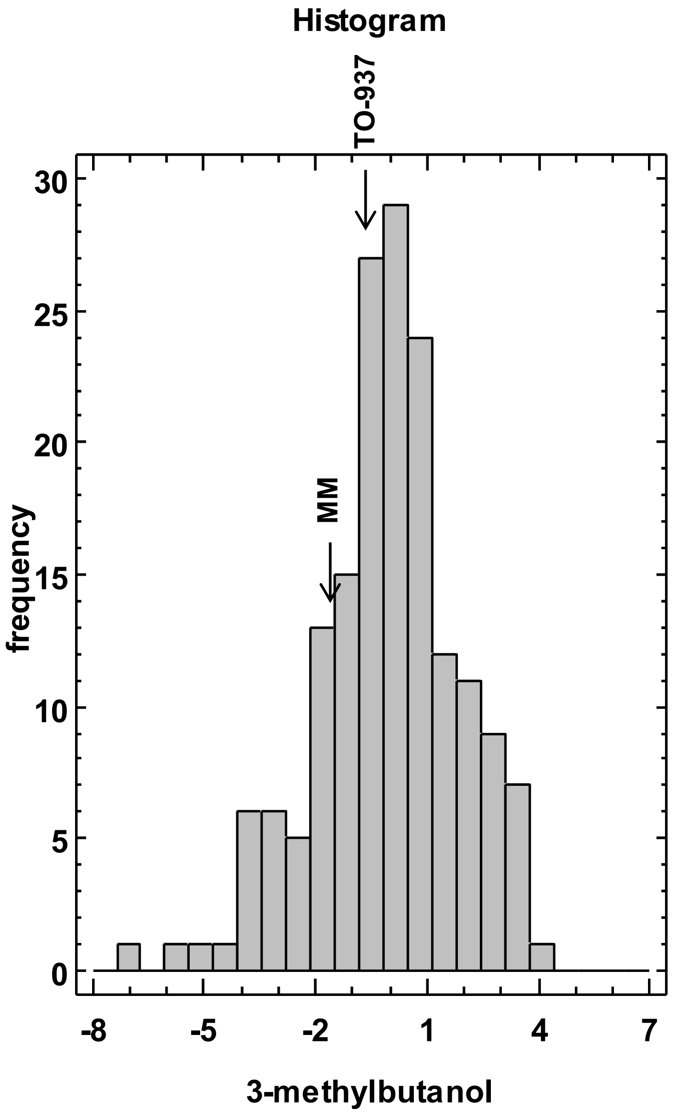

RILs season 3

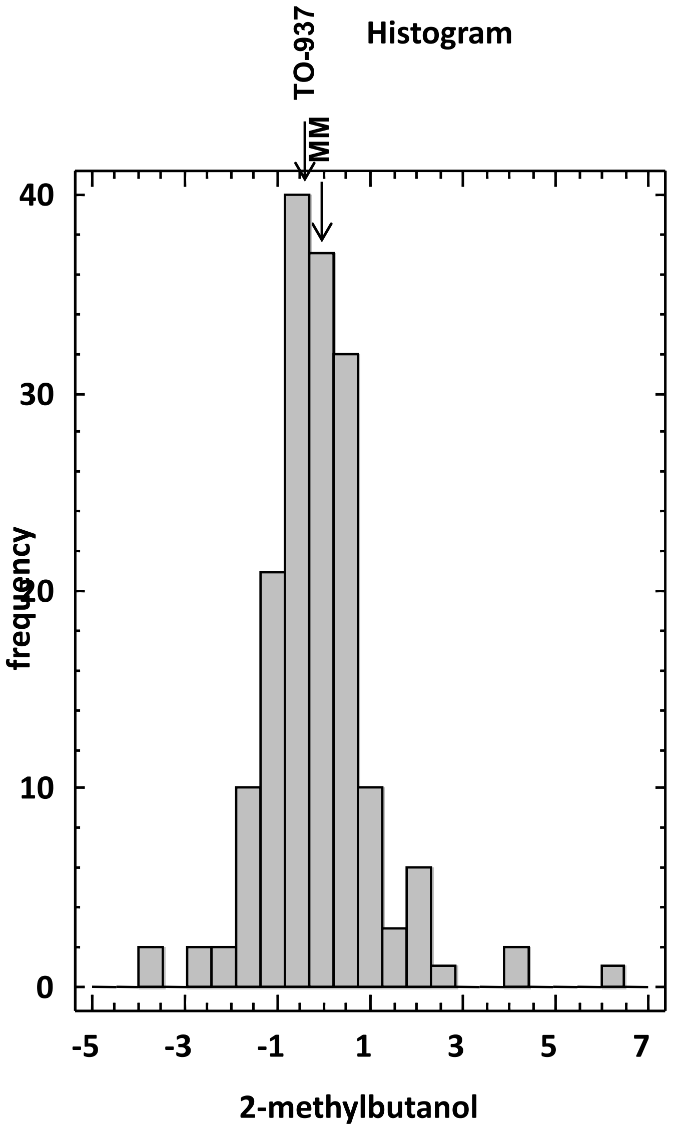

RILs season 3

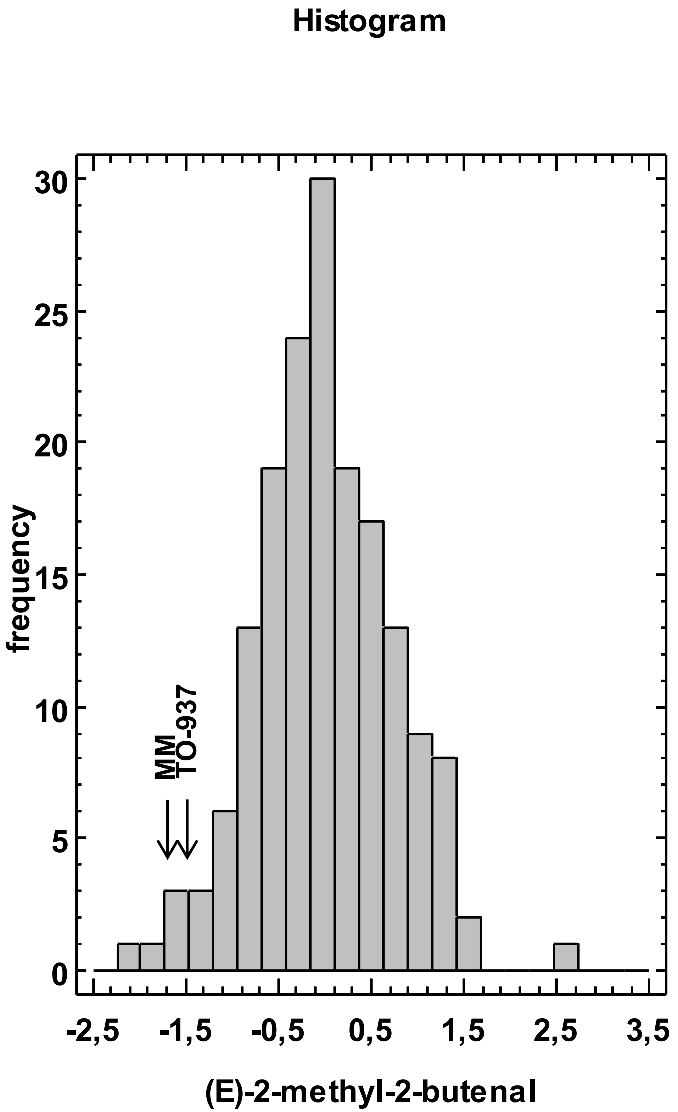

RILs season 3

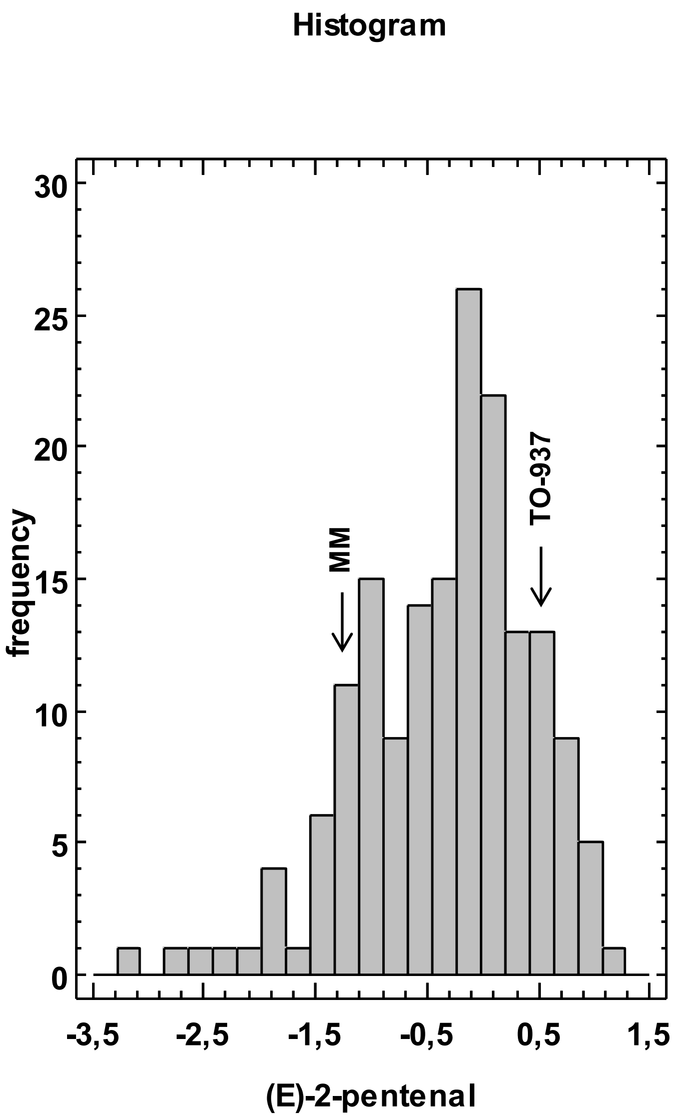

RILs season 3

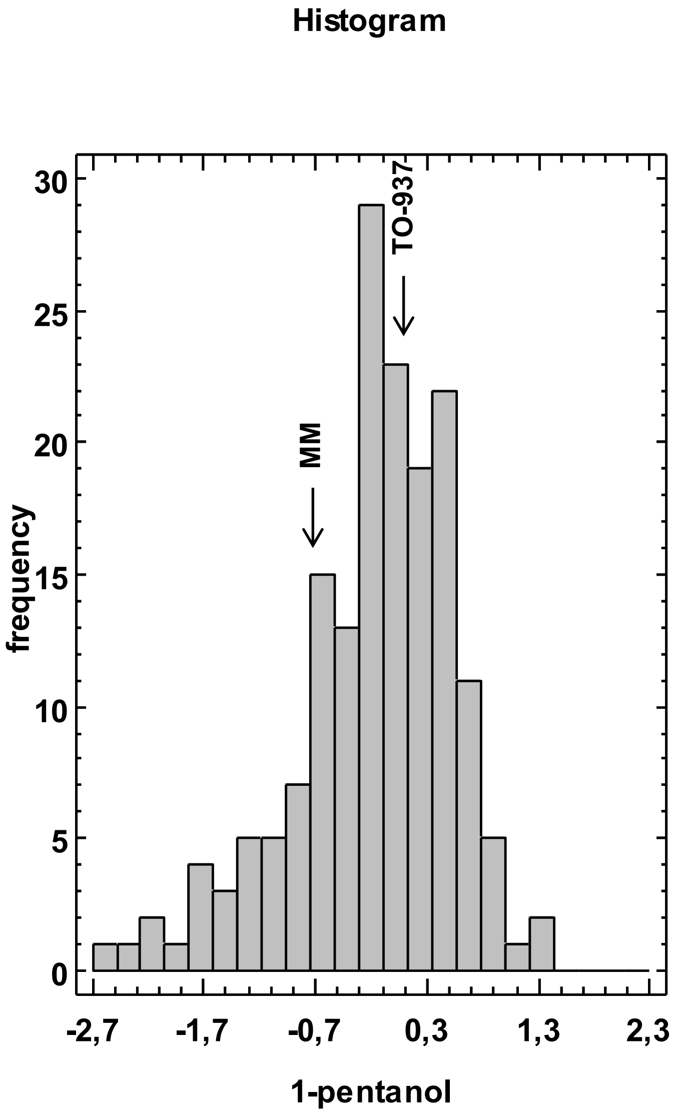

RILs season 3

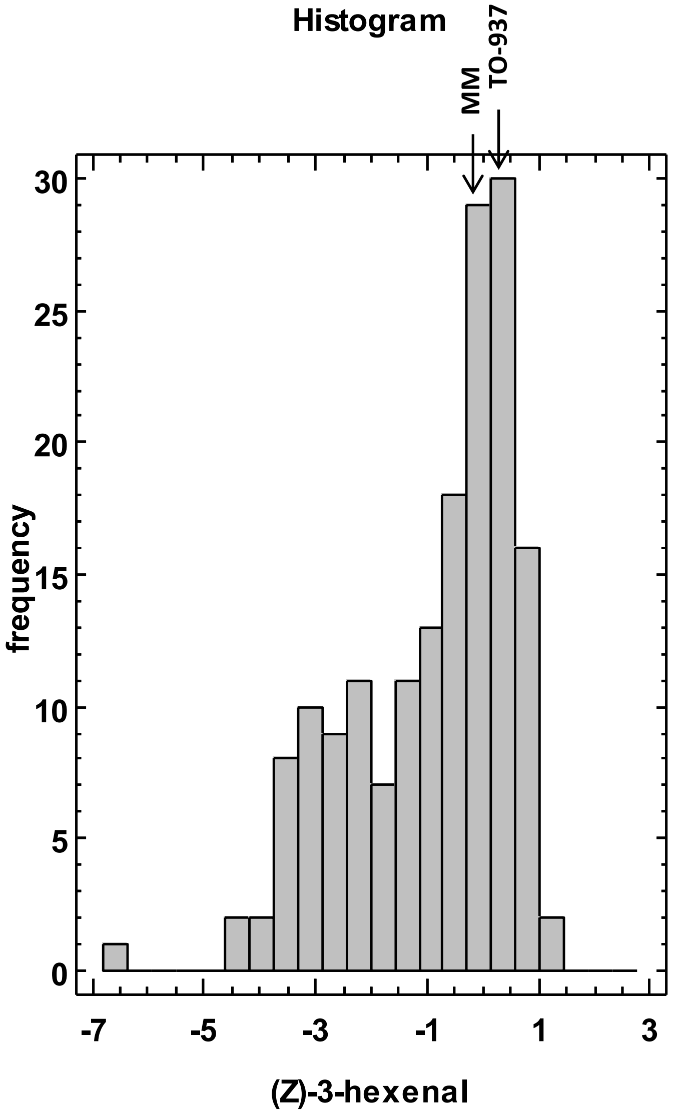

RILs season 3

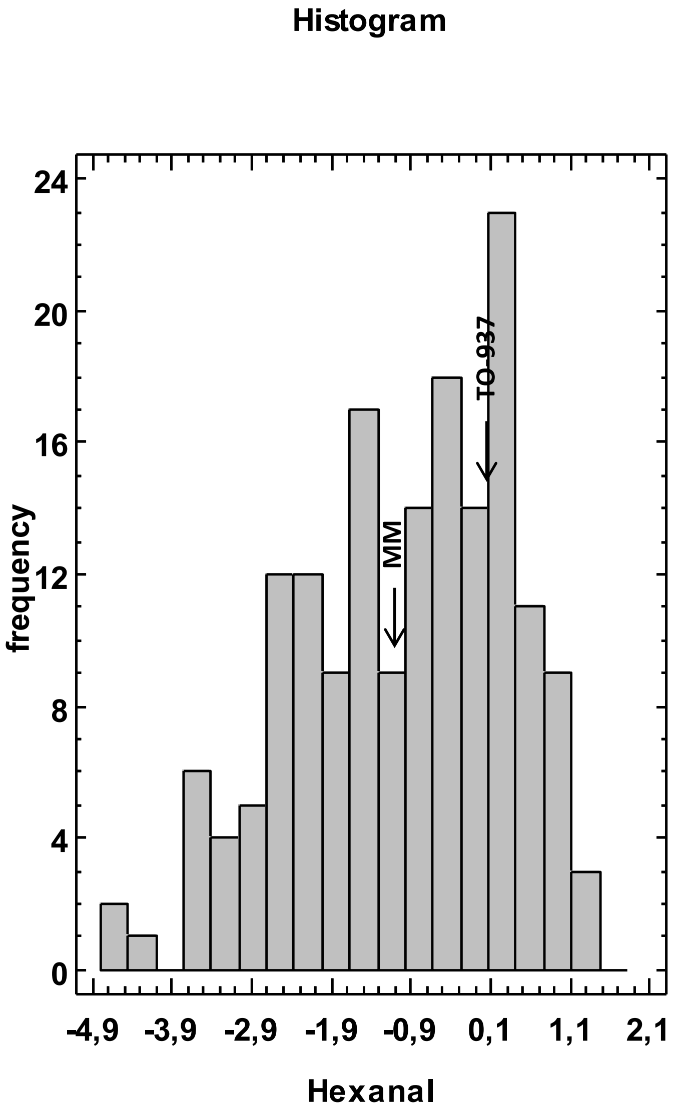

RILs season 3

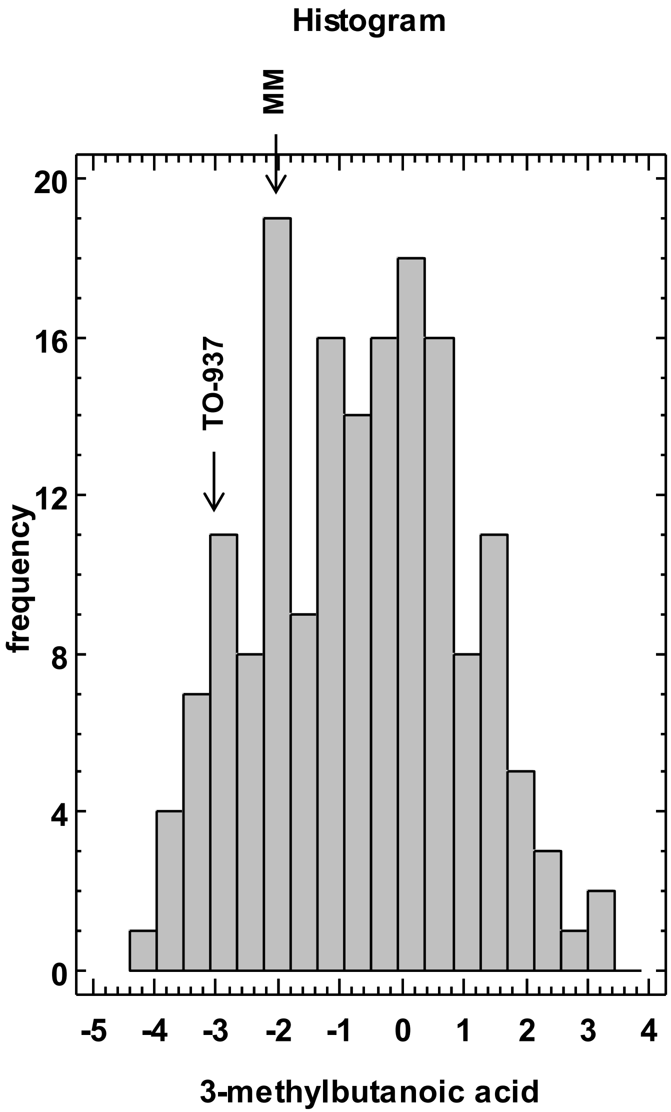

RILs season 3

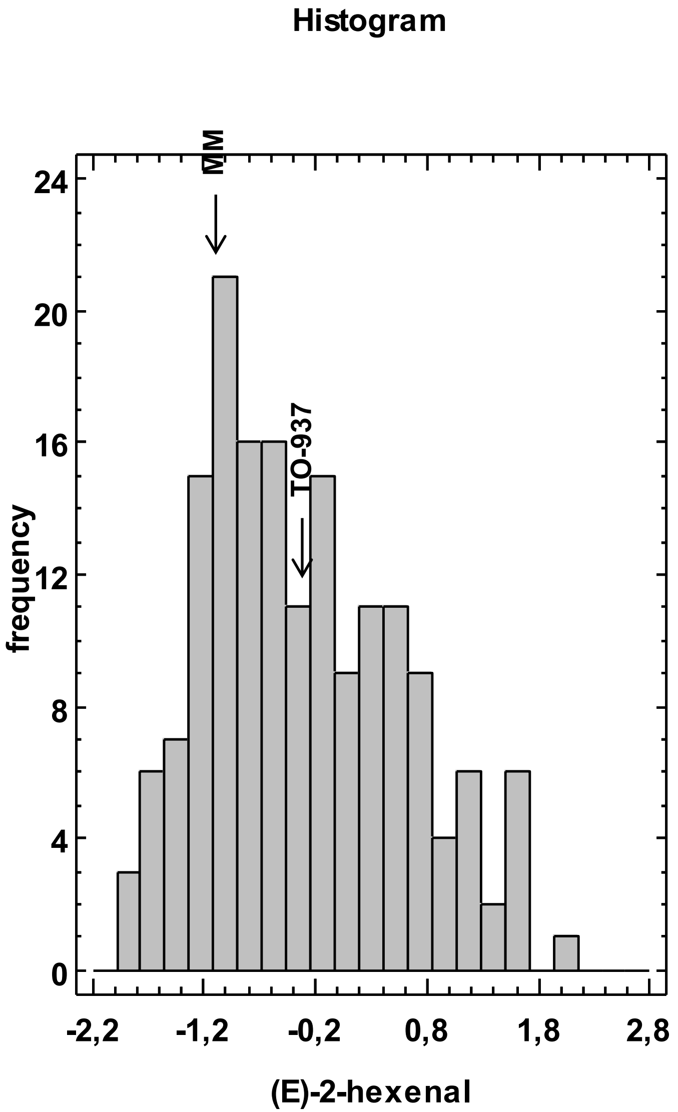

RILs season 3

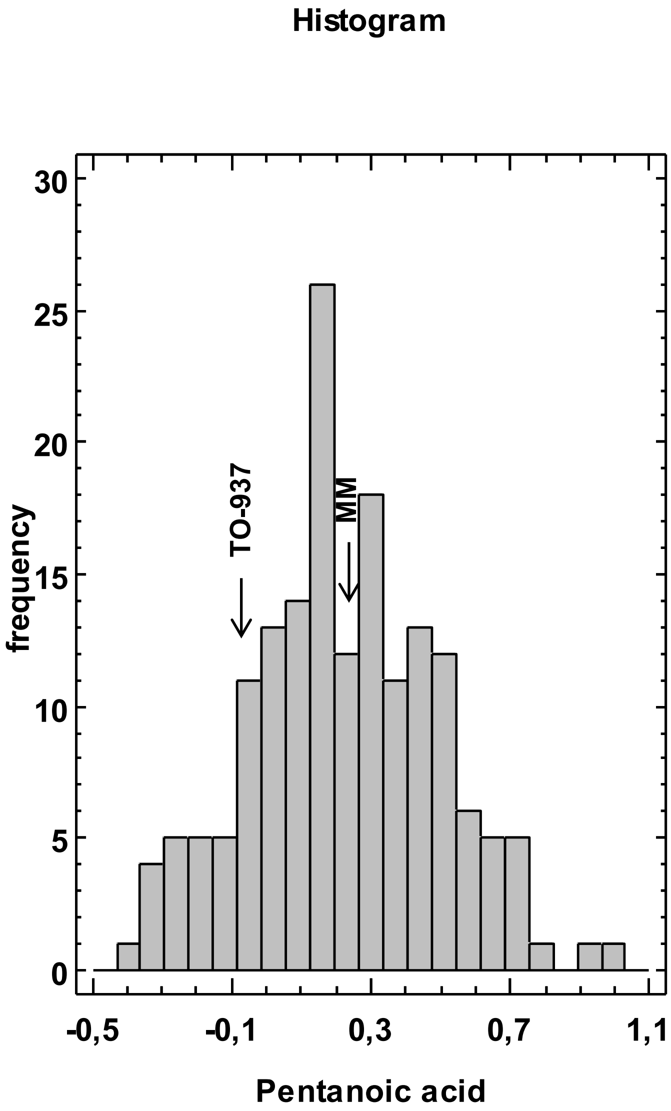

RILs season 3

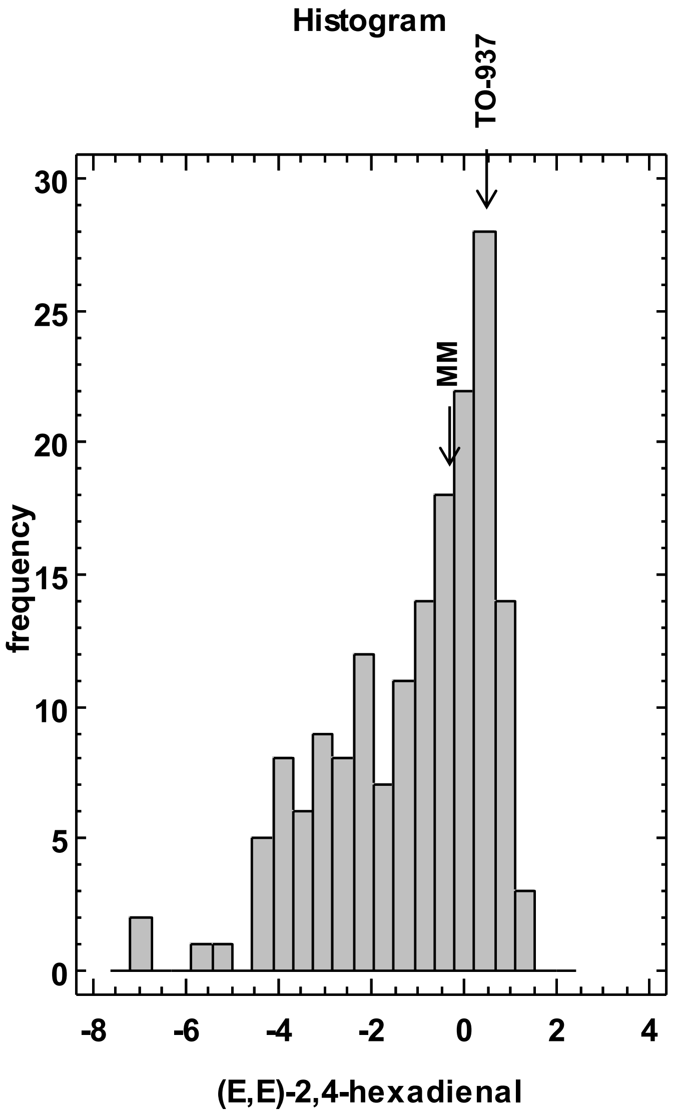

RILs season 3

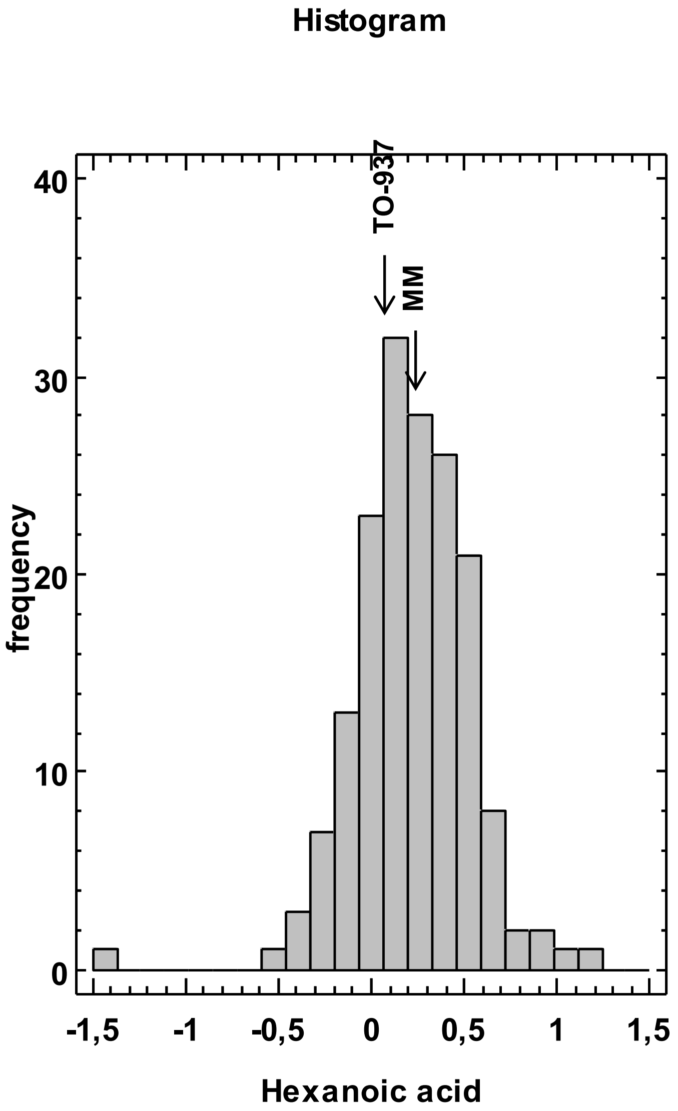

RILs season 3

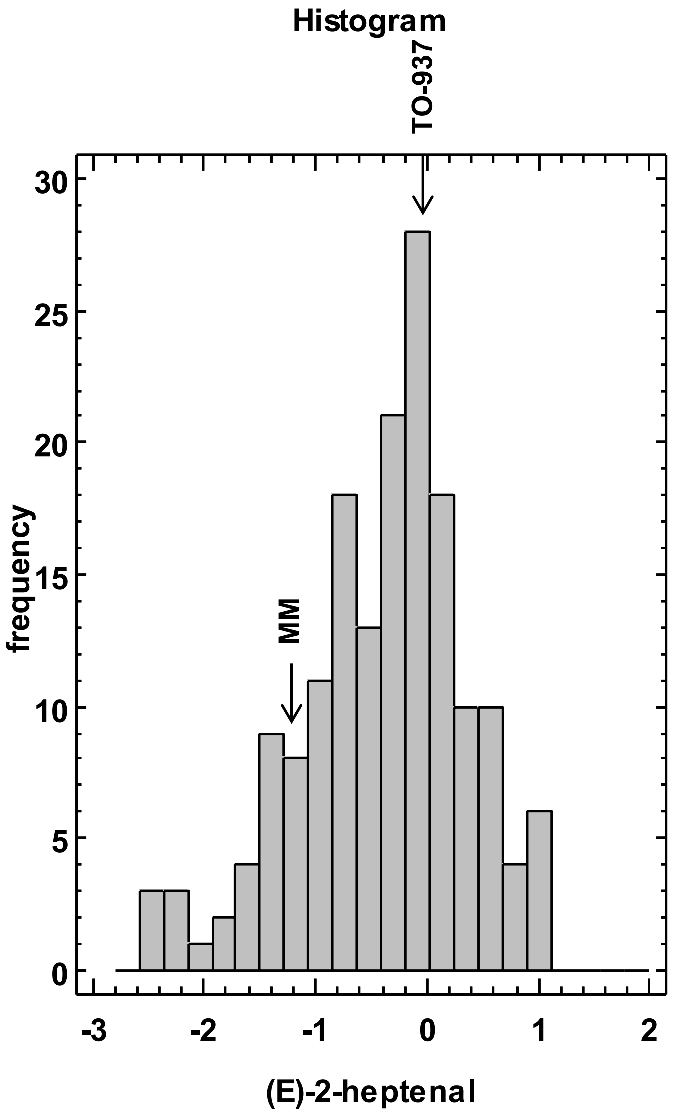

RILs season 3

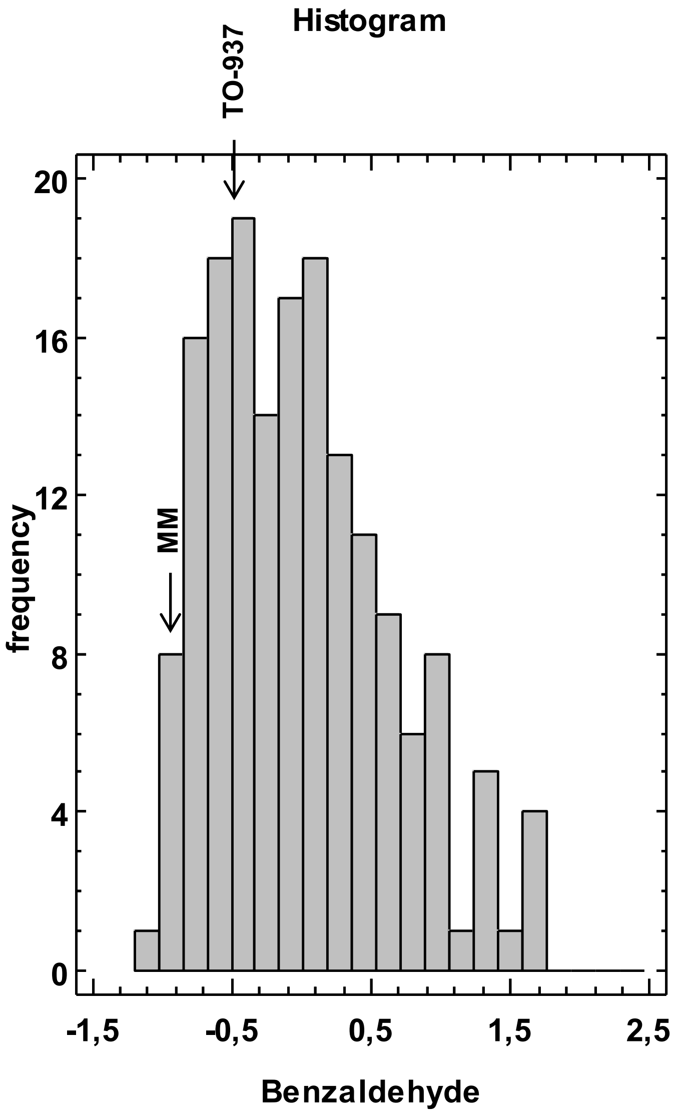

RILs season 3

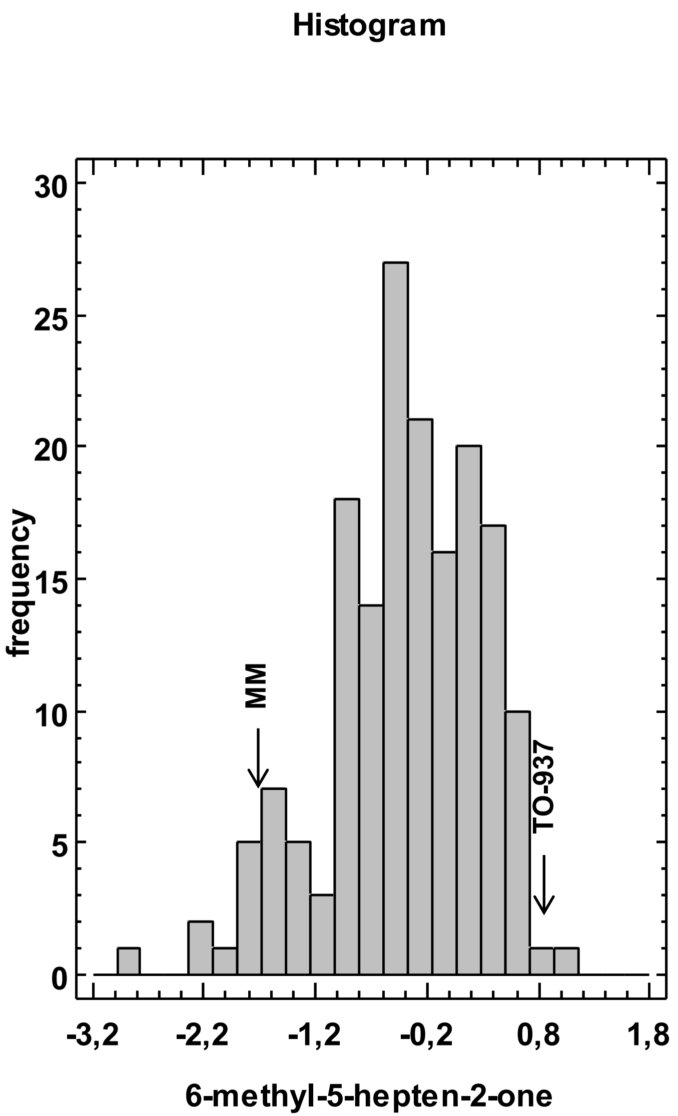

RILs season 3

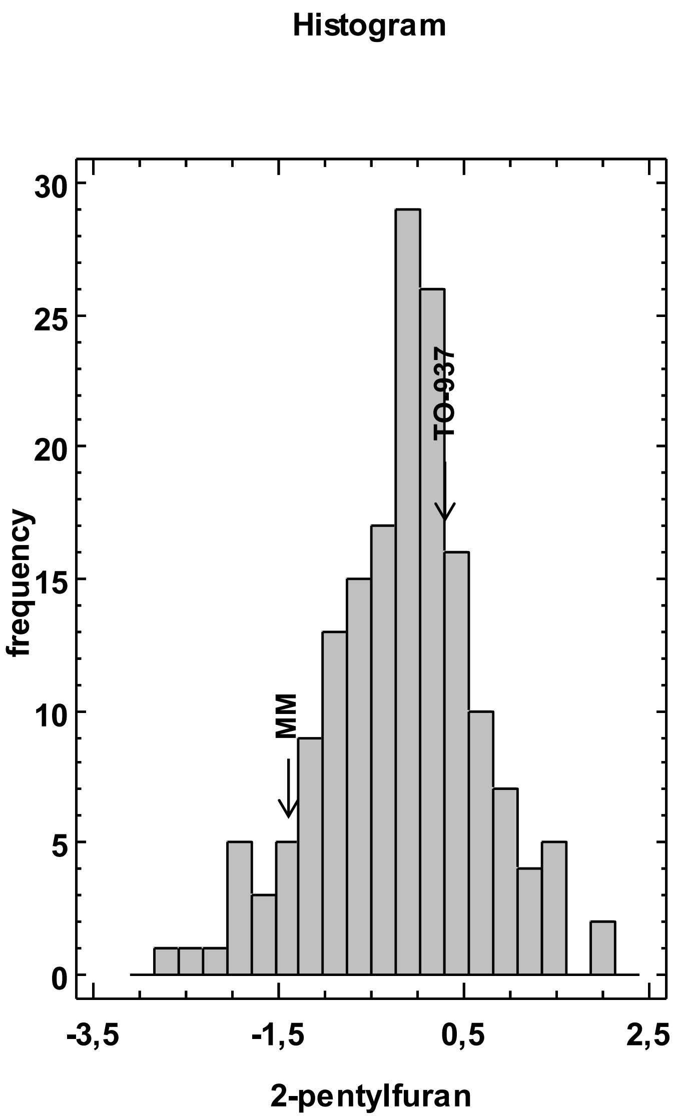

RILs season 3

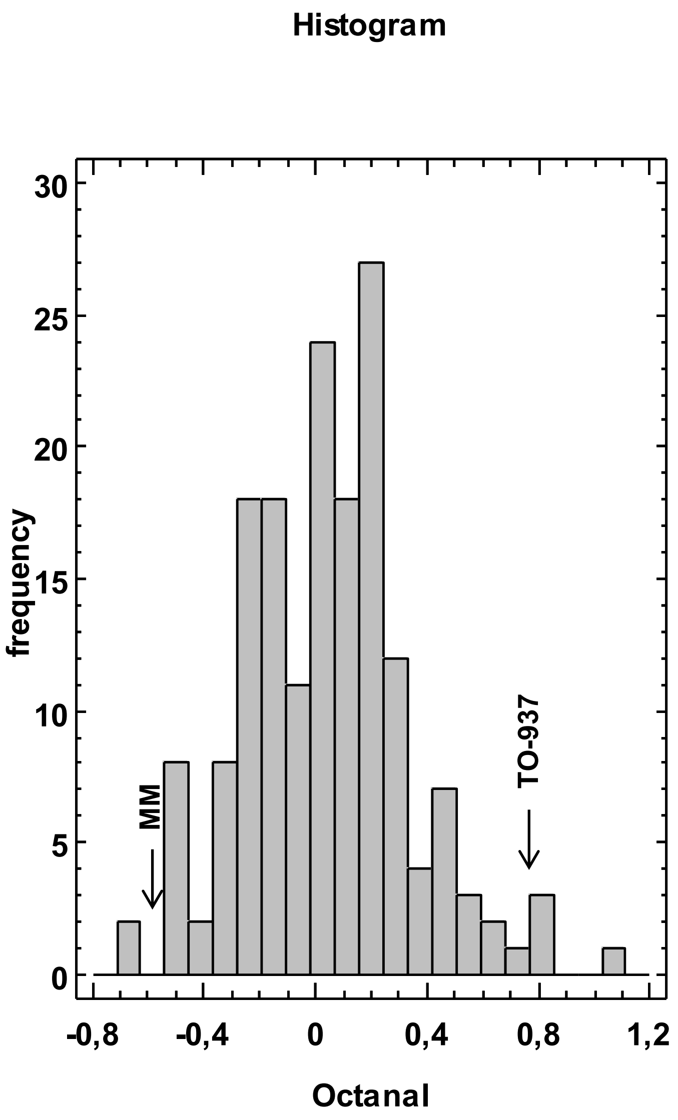

RILs season 3

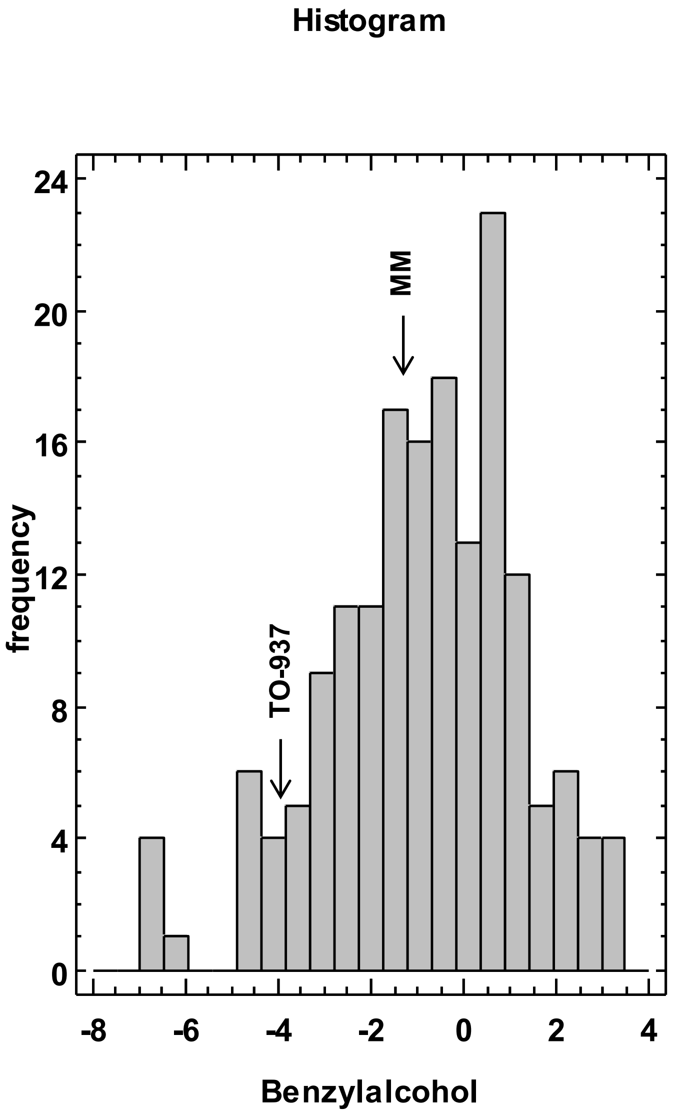

RILs season 3

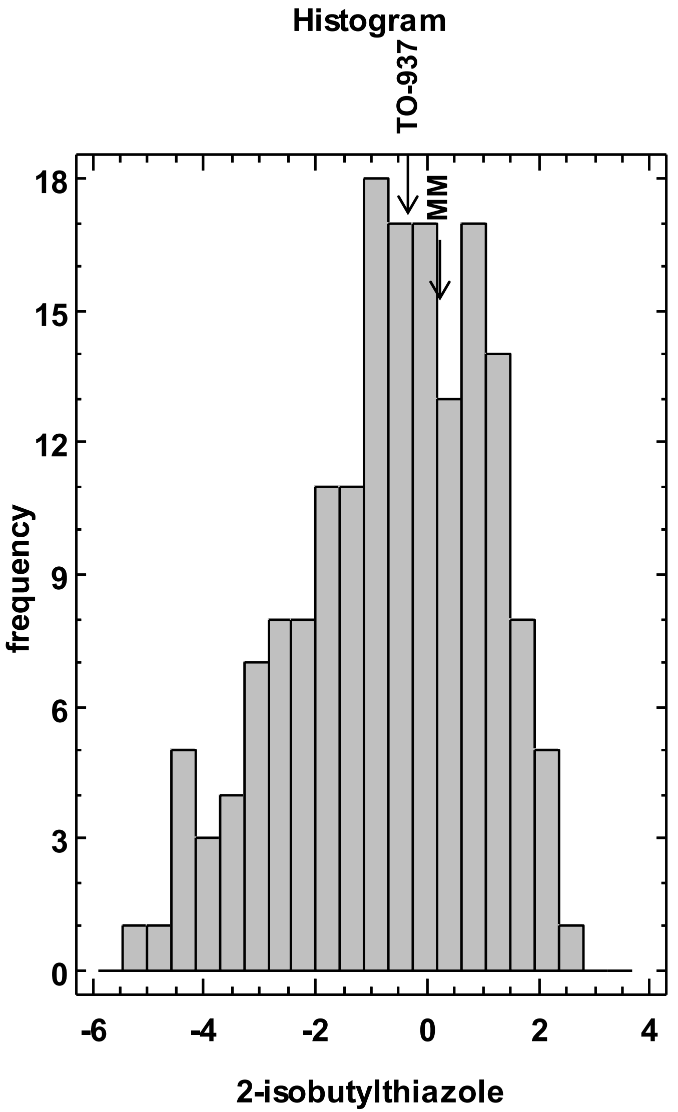

RILs season 3

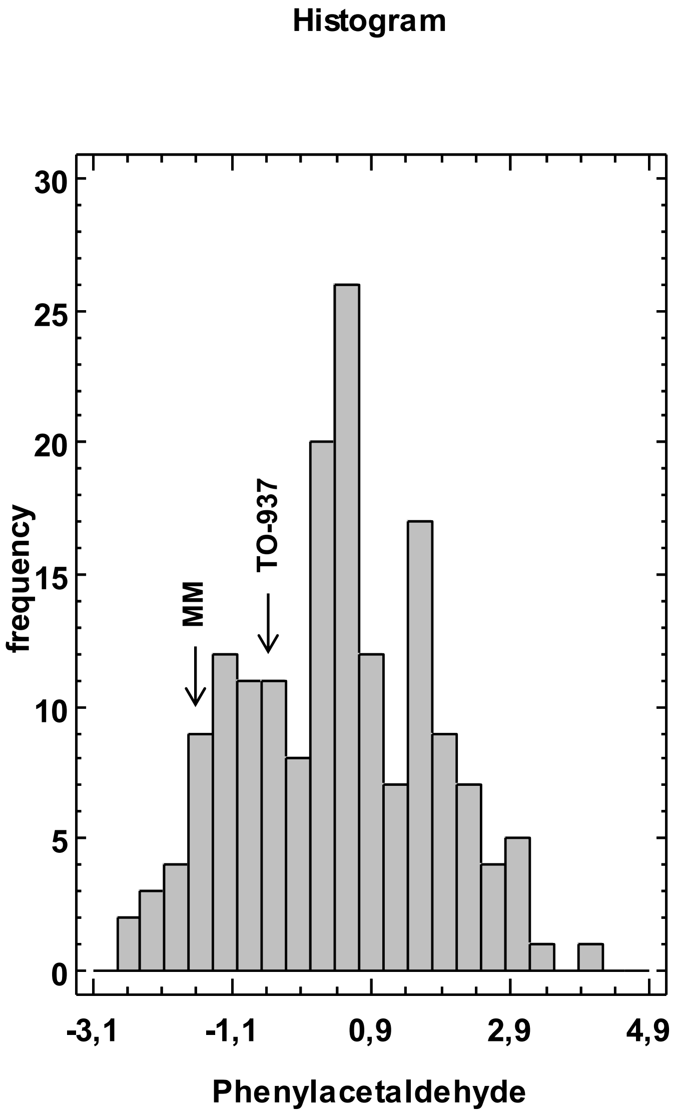

RILs season 3

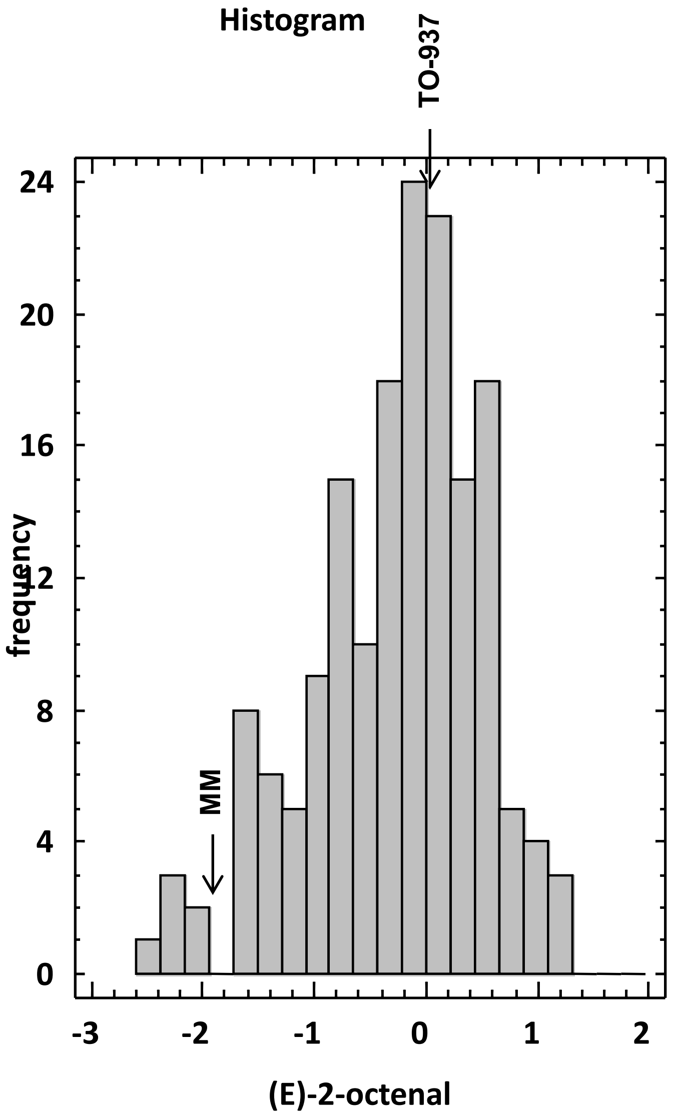

RILs season 3

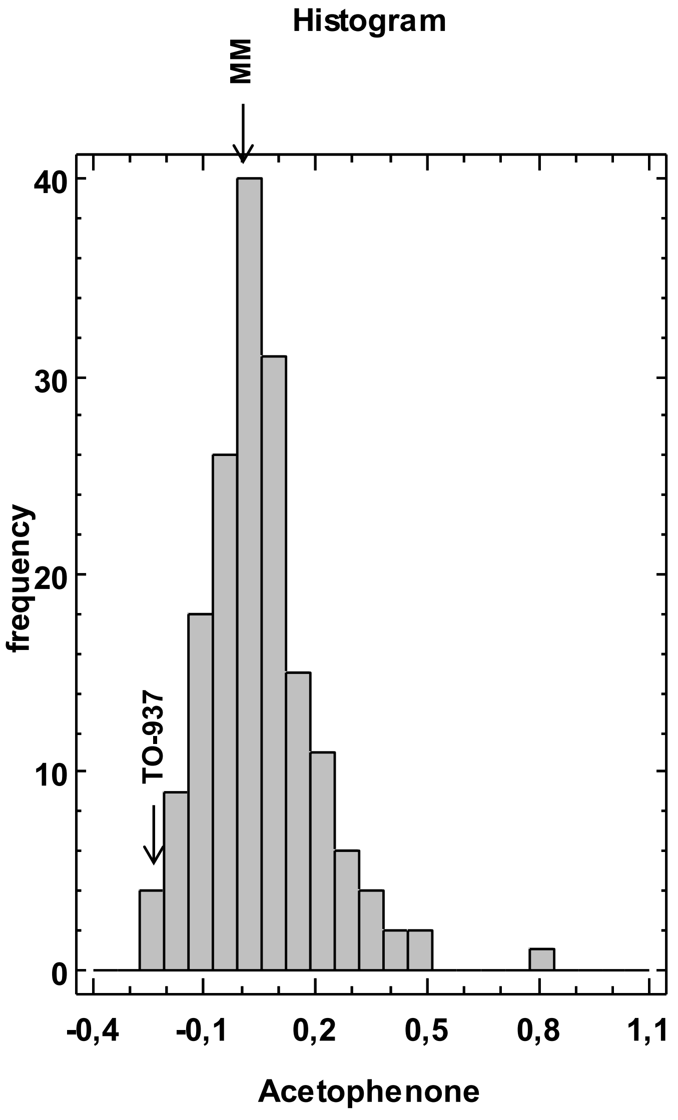

RILs season 3

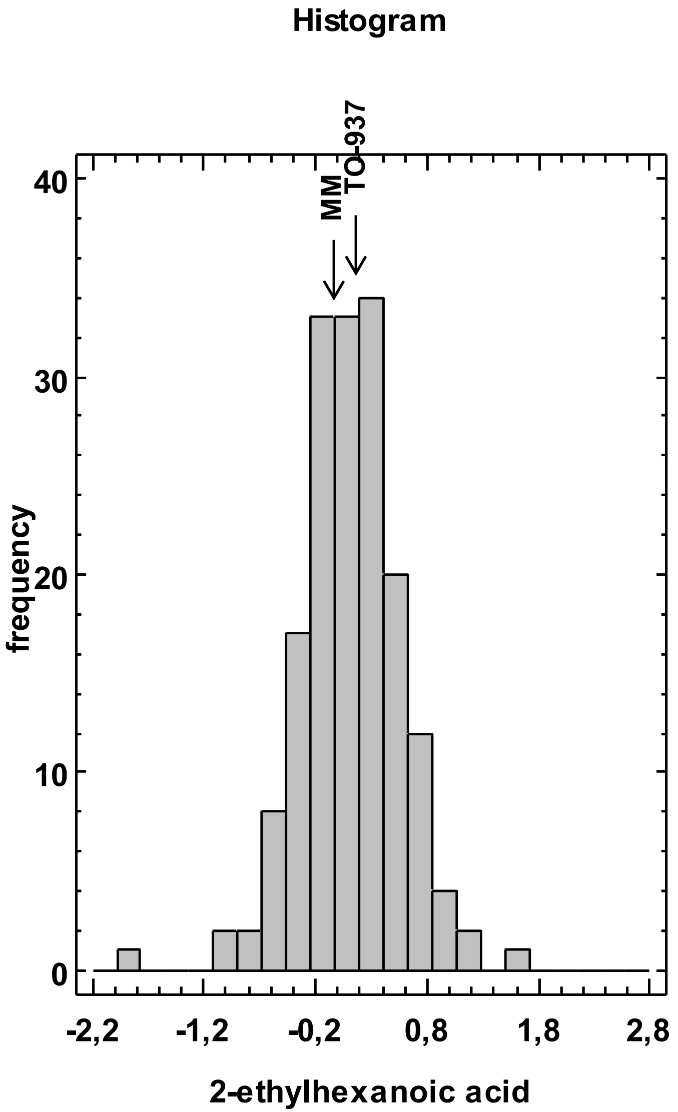

RILs season 3

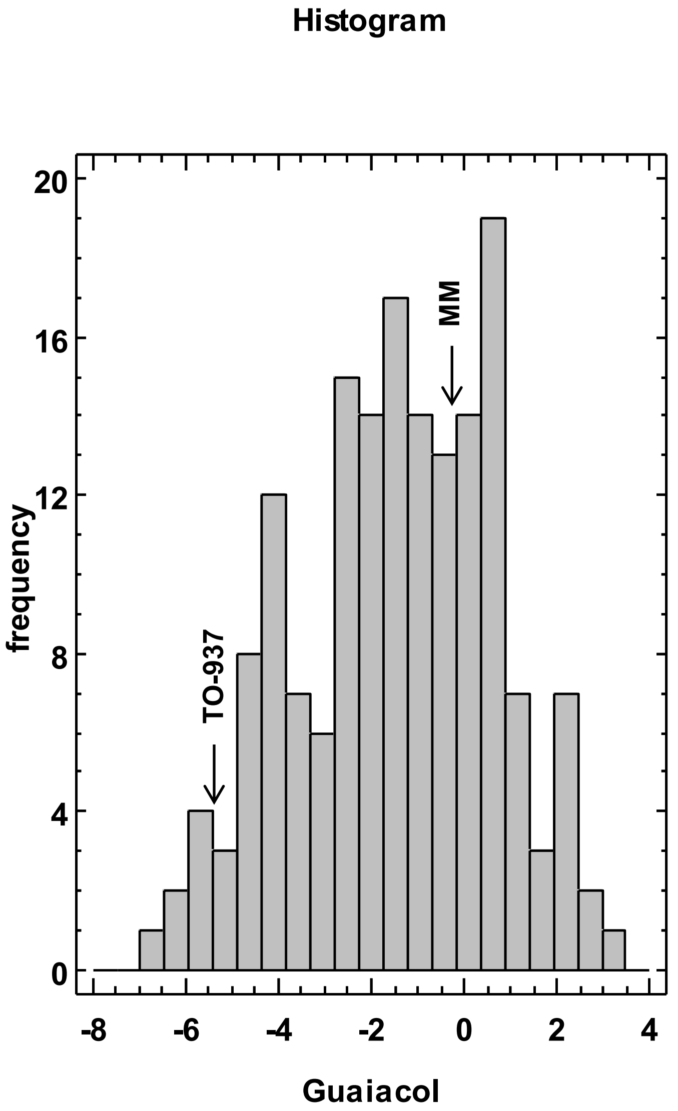

RILs season 3

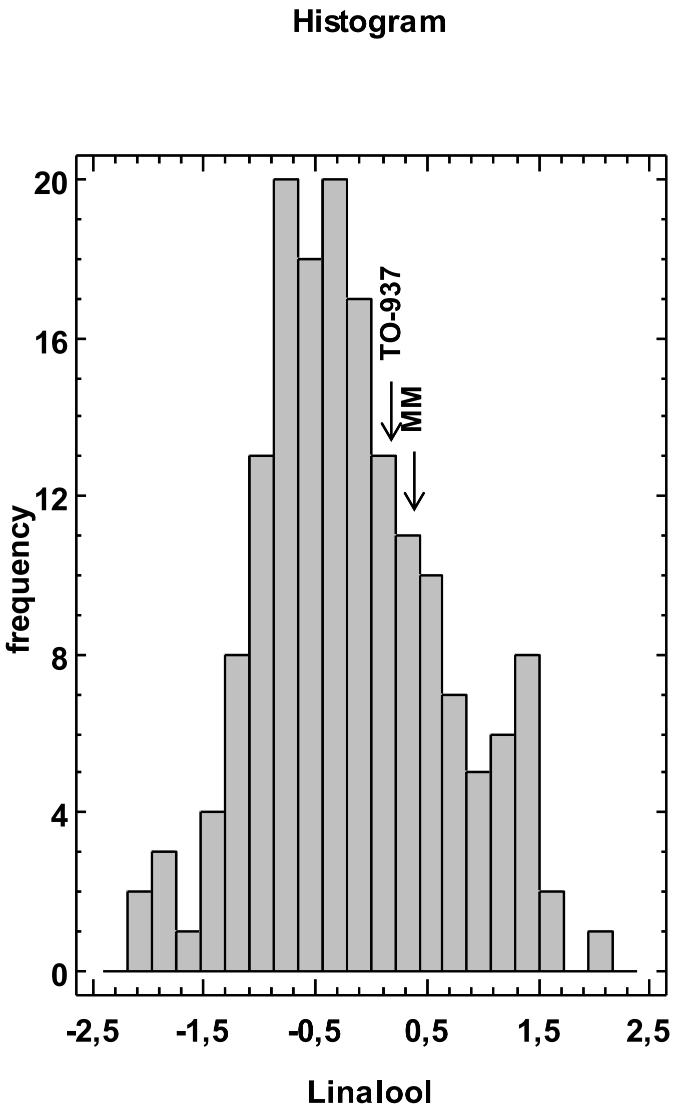

RILs season 3

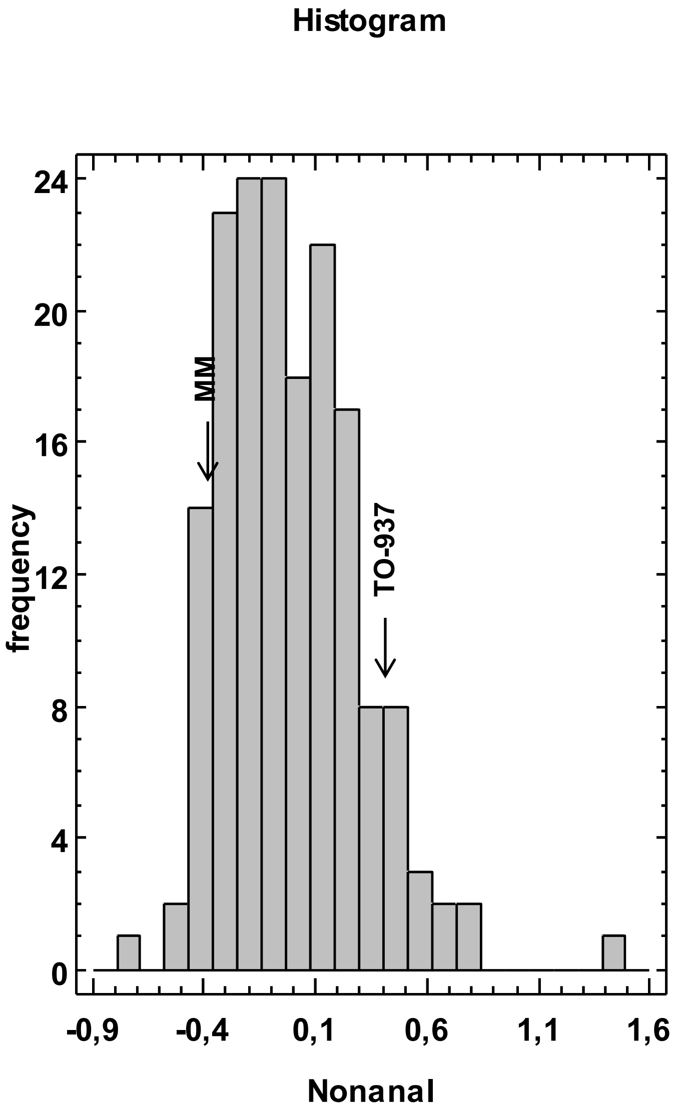

RILs season 3

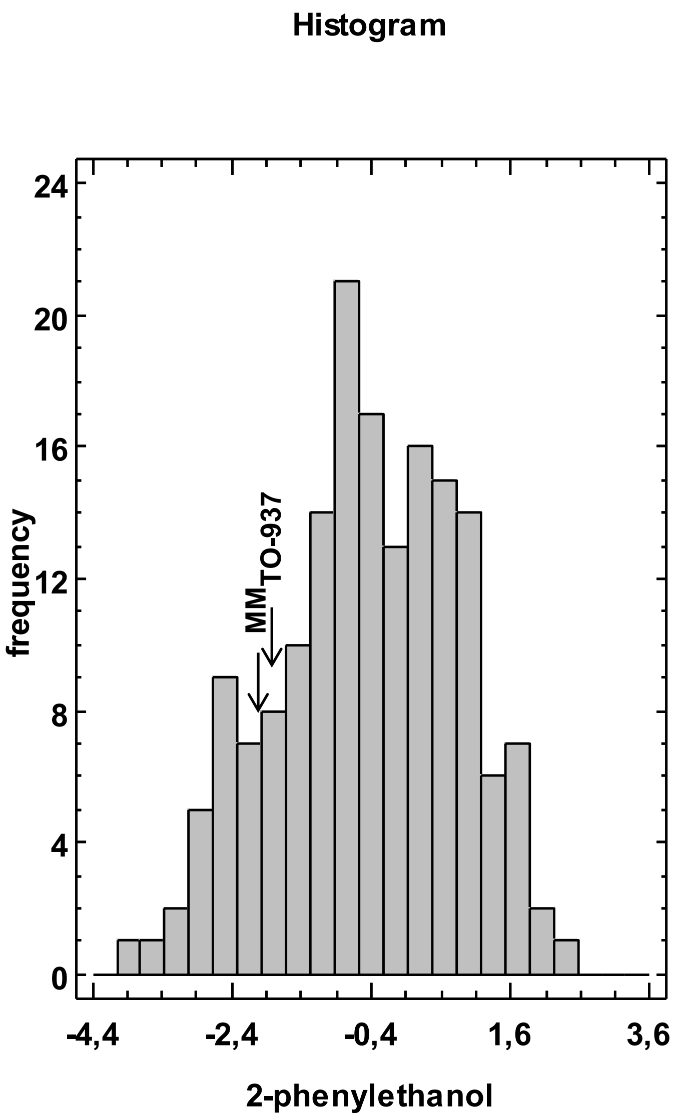

RILs season 3

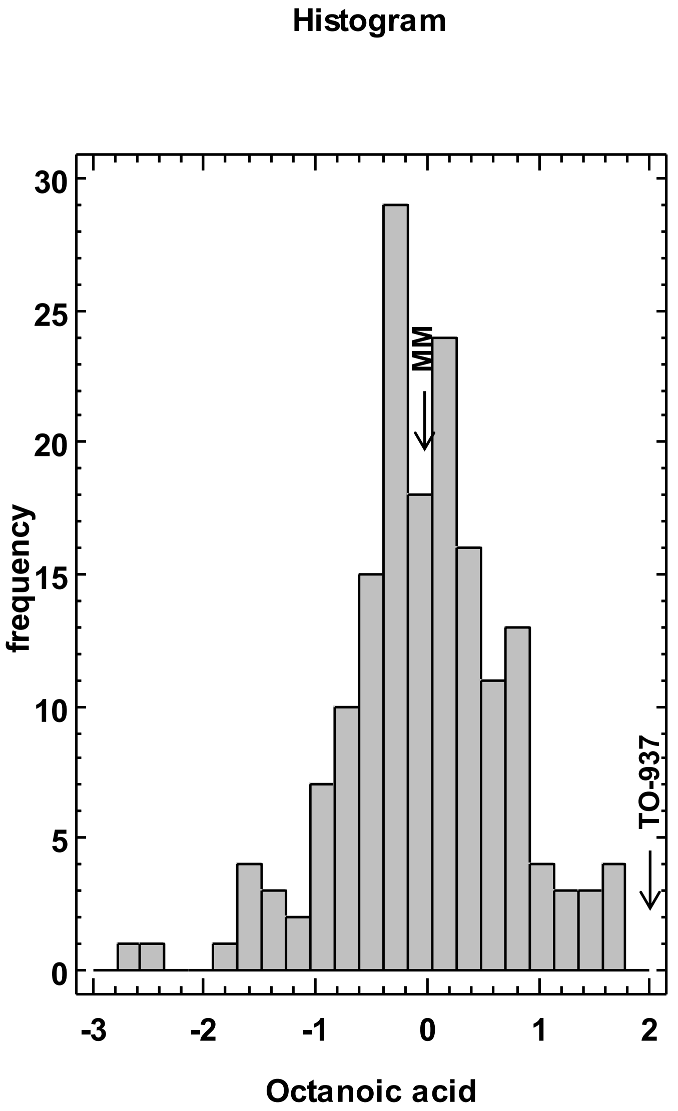

RILs season 3

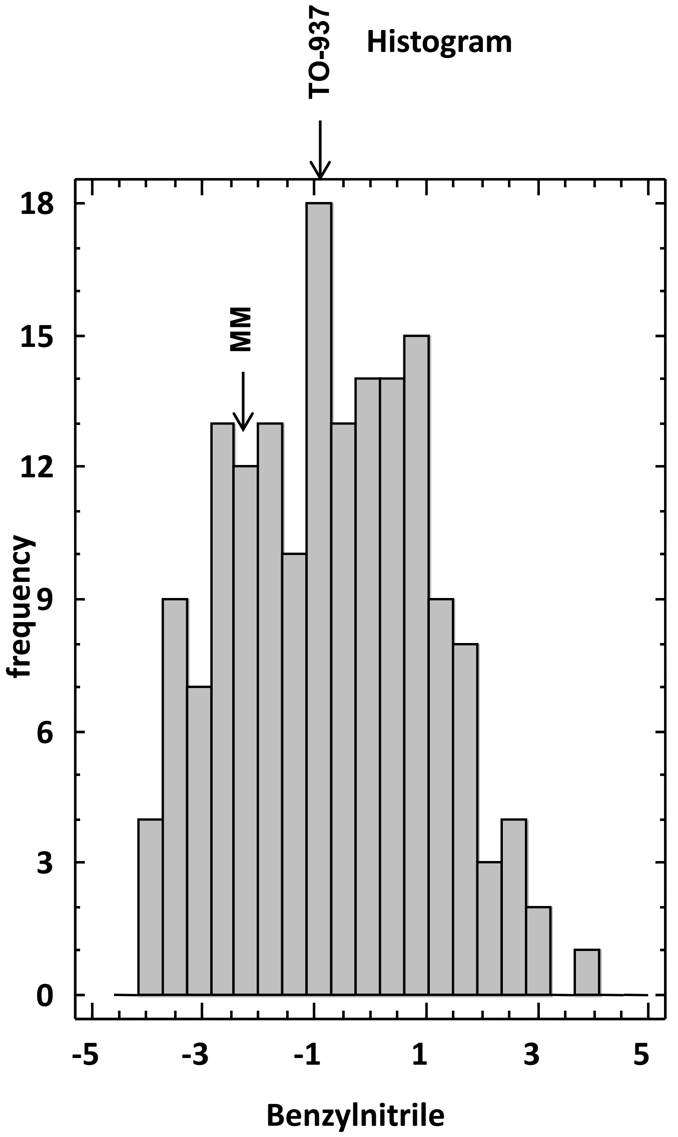

RILs season 3

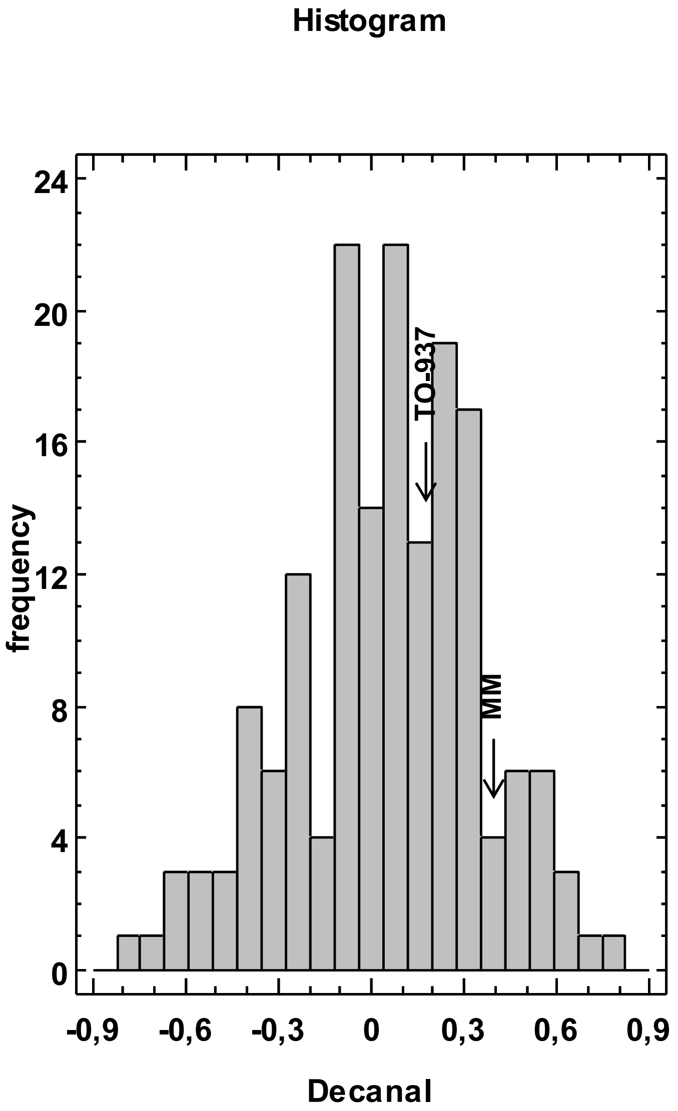

RILs season 3

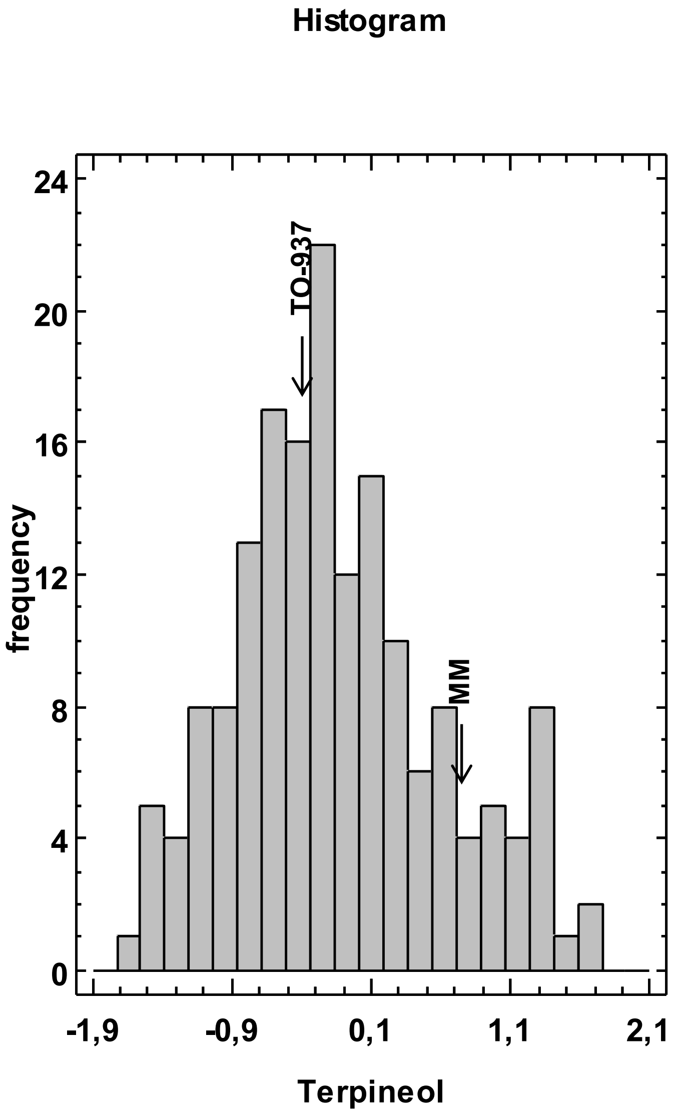

RILs season 3

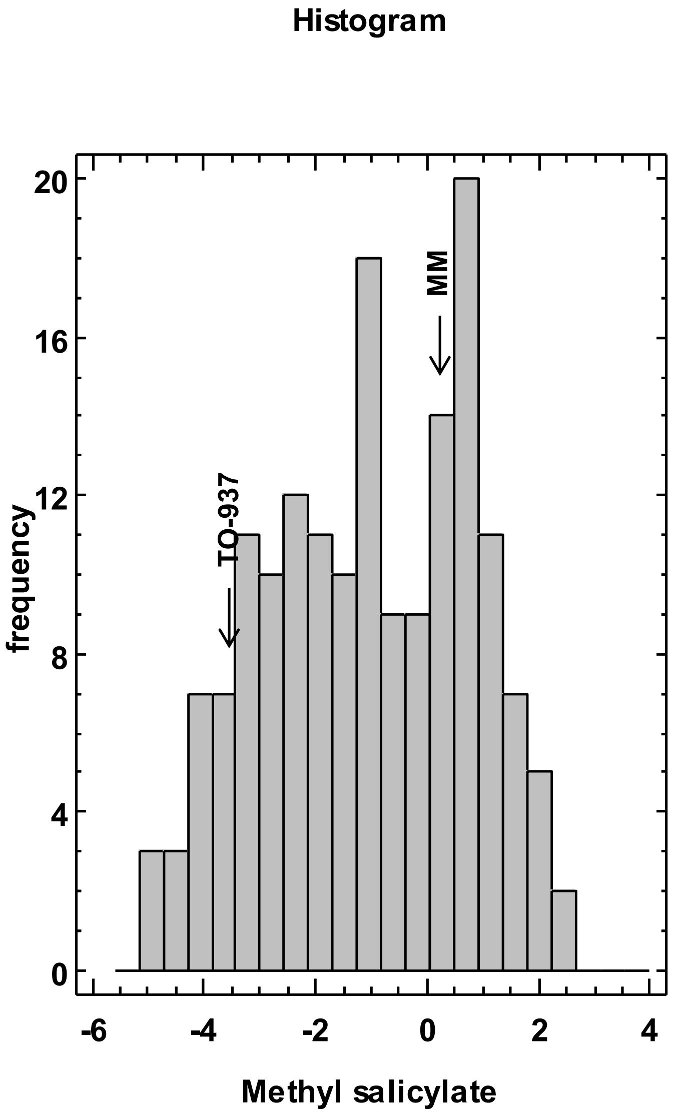

RILs season 3

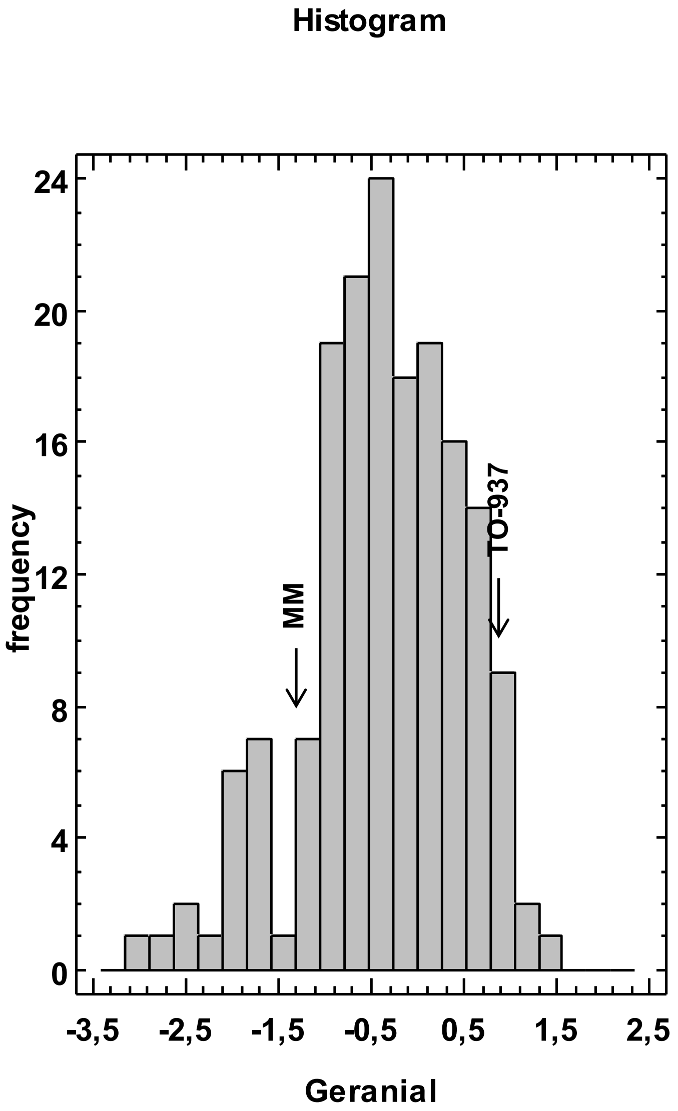

RILs season 3

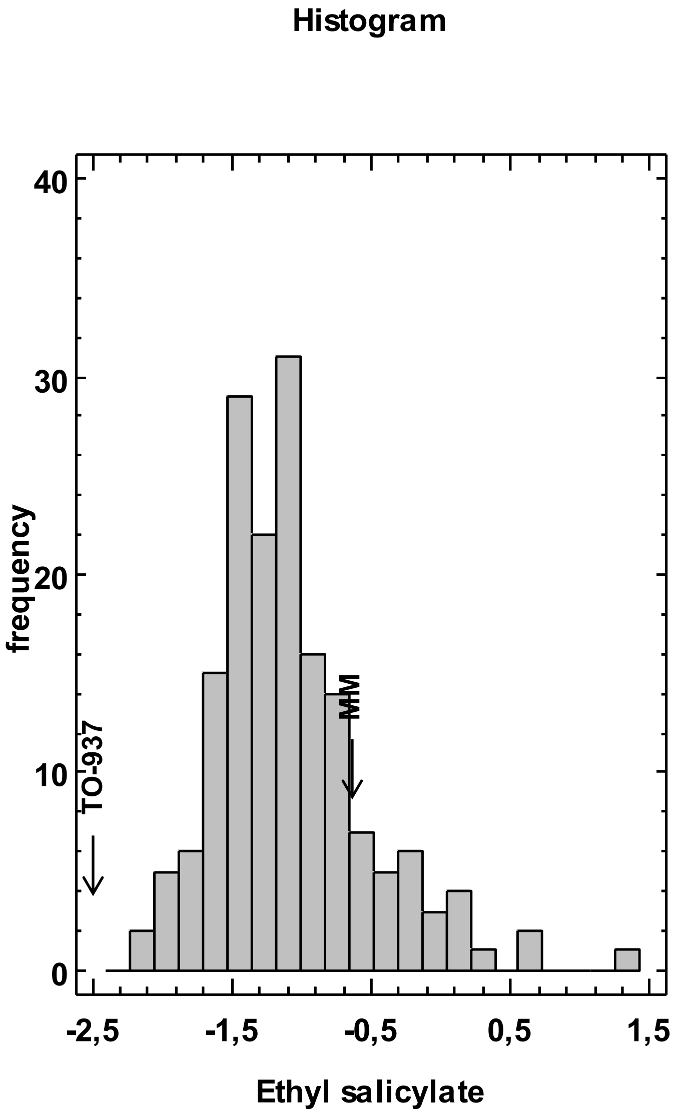

RILs season 3

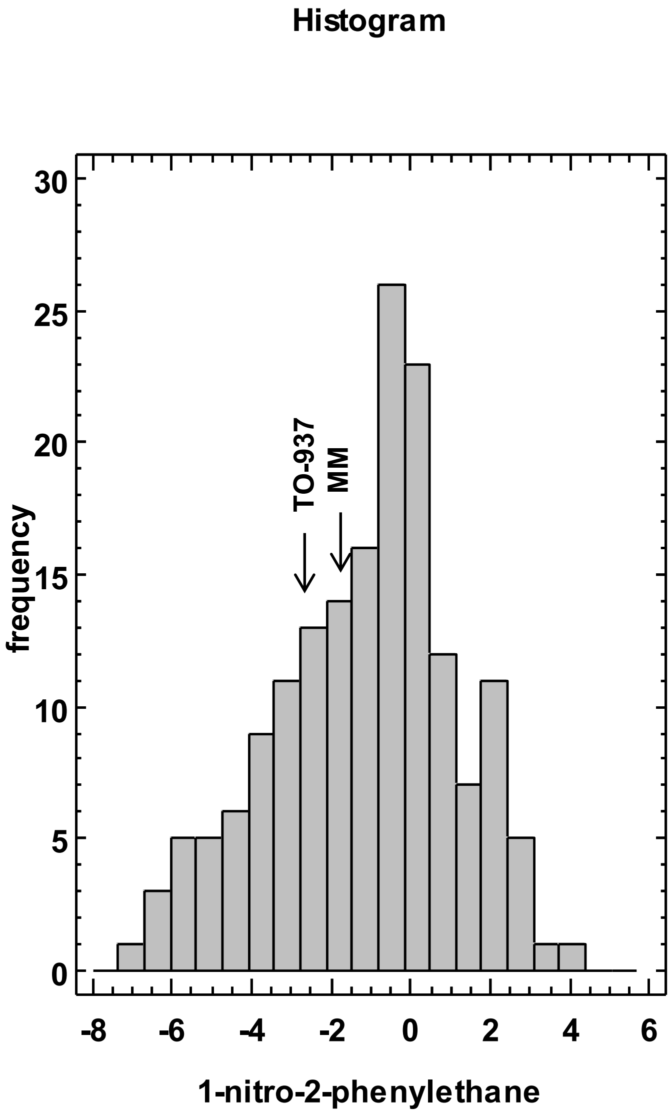

RILs season 3

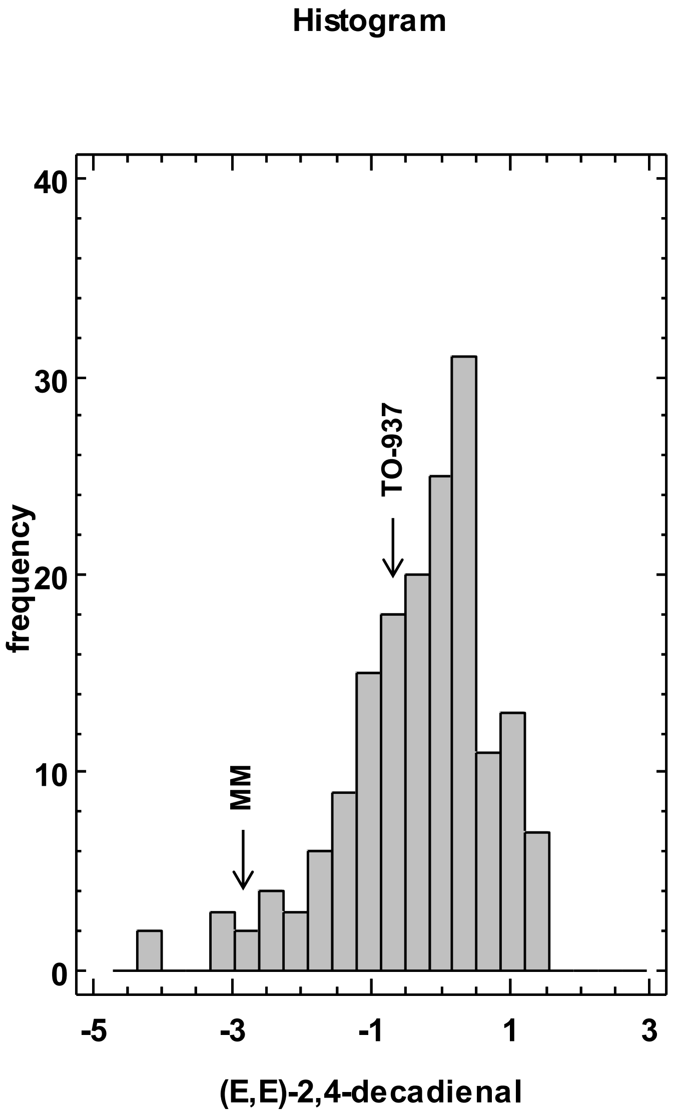

RILs season 3

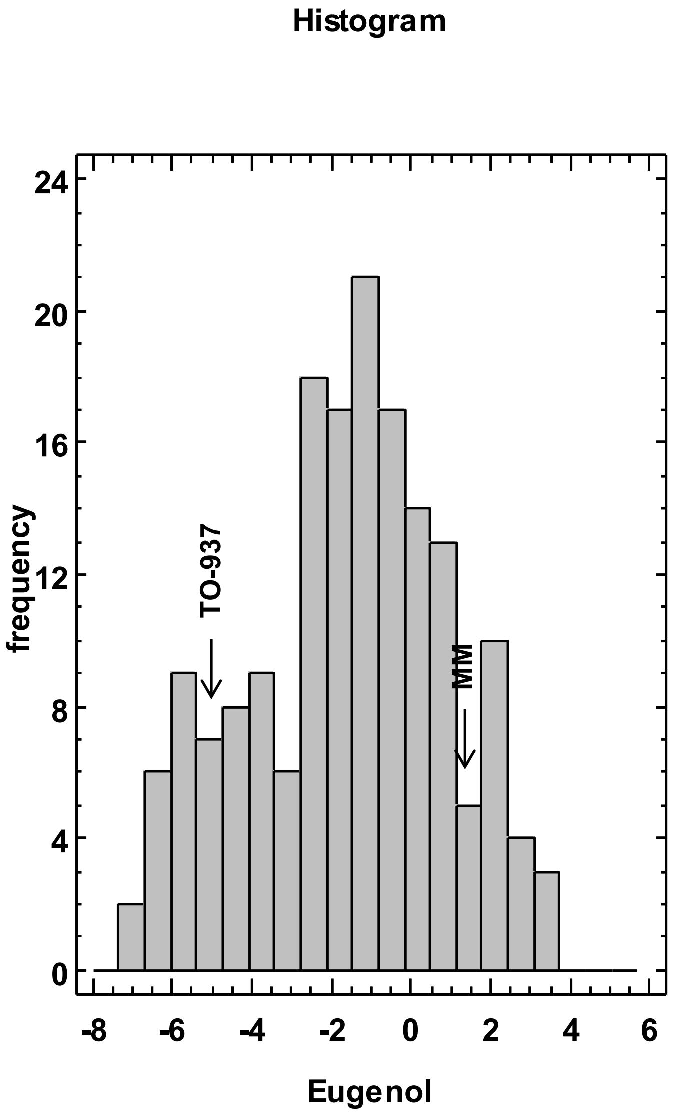

RILs season 3

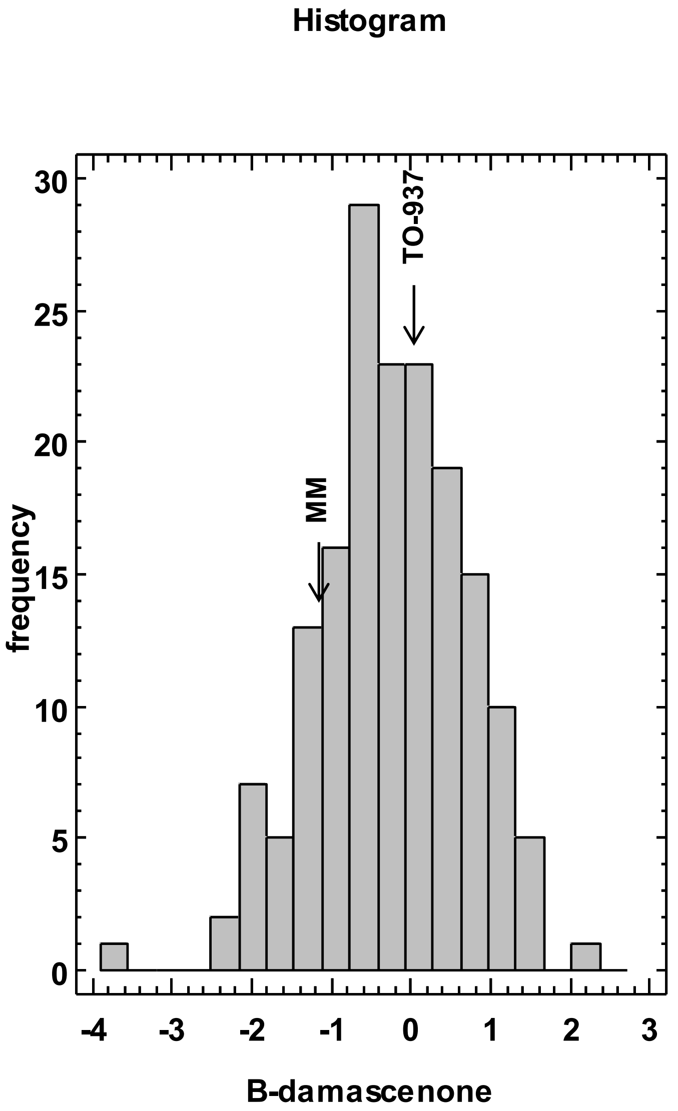

RILs season 3

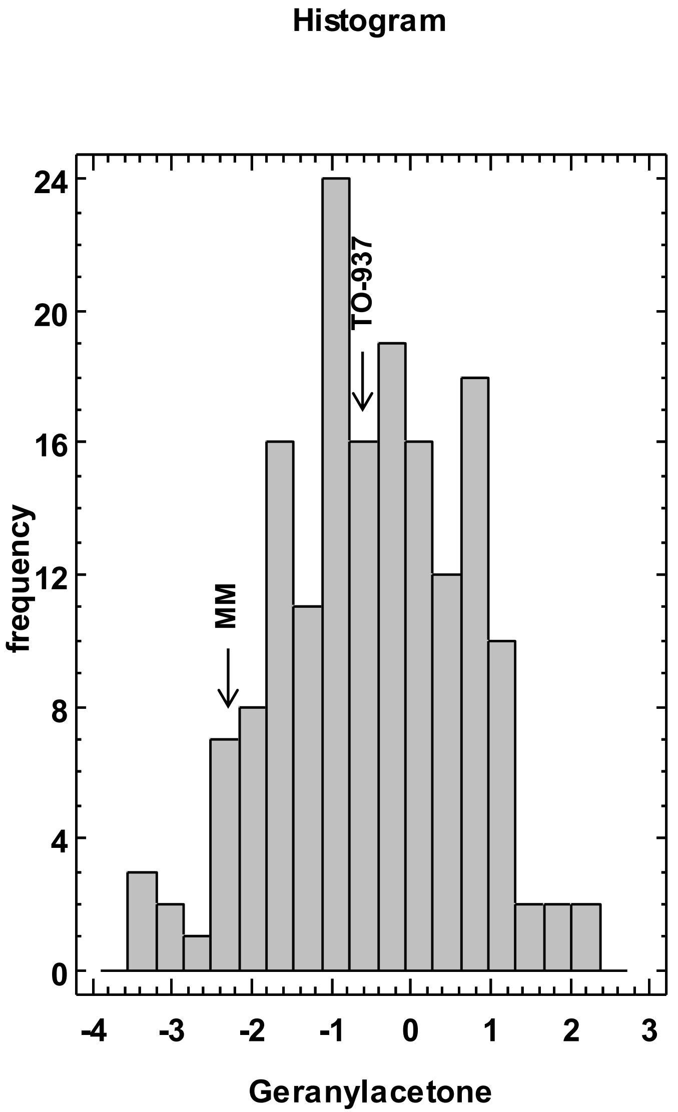

RILs season 3

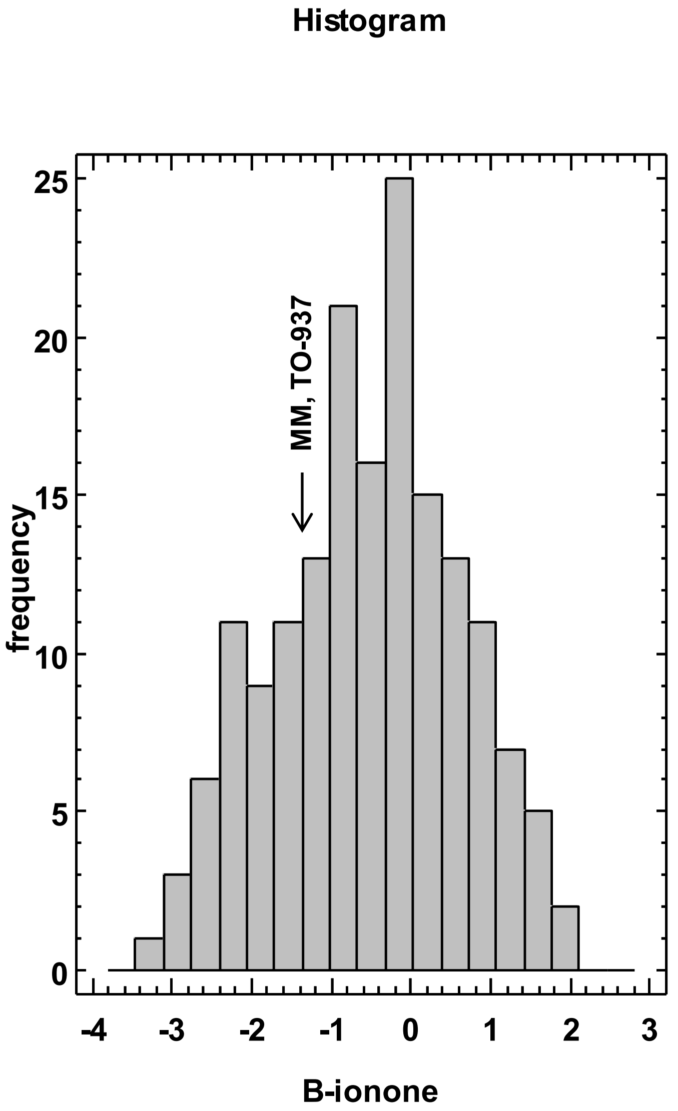

RILs season 3

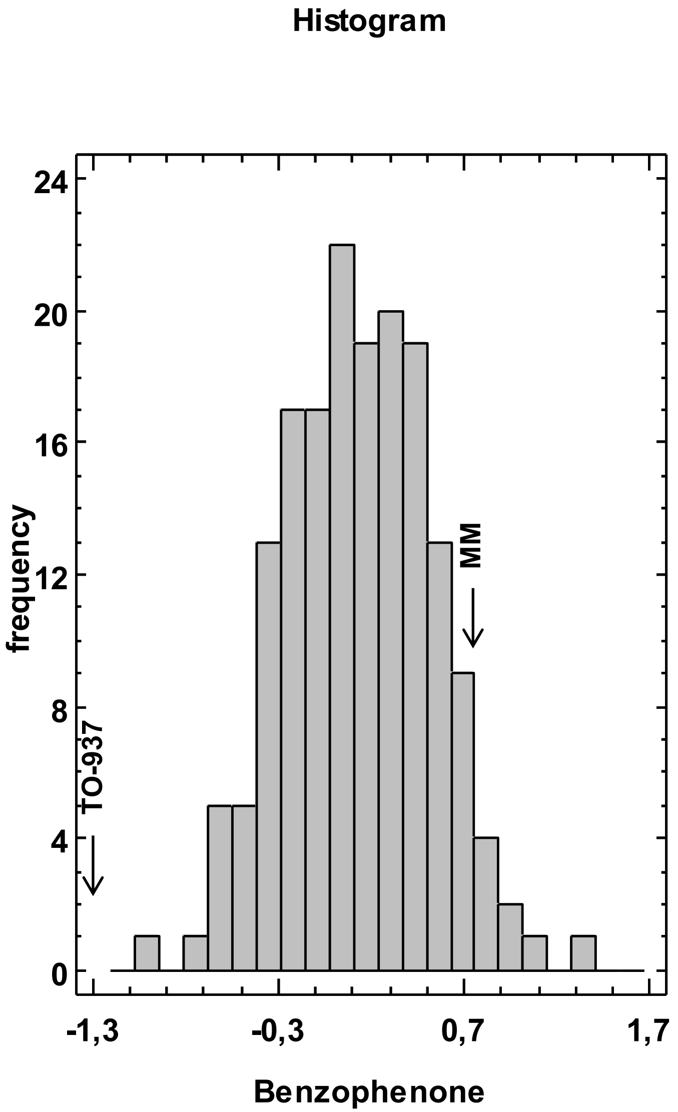

Figure S3. Hierarchical clusters of volatiles in seasons 2 and 3 independently and an evaluation of the degree of similarity.

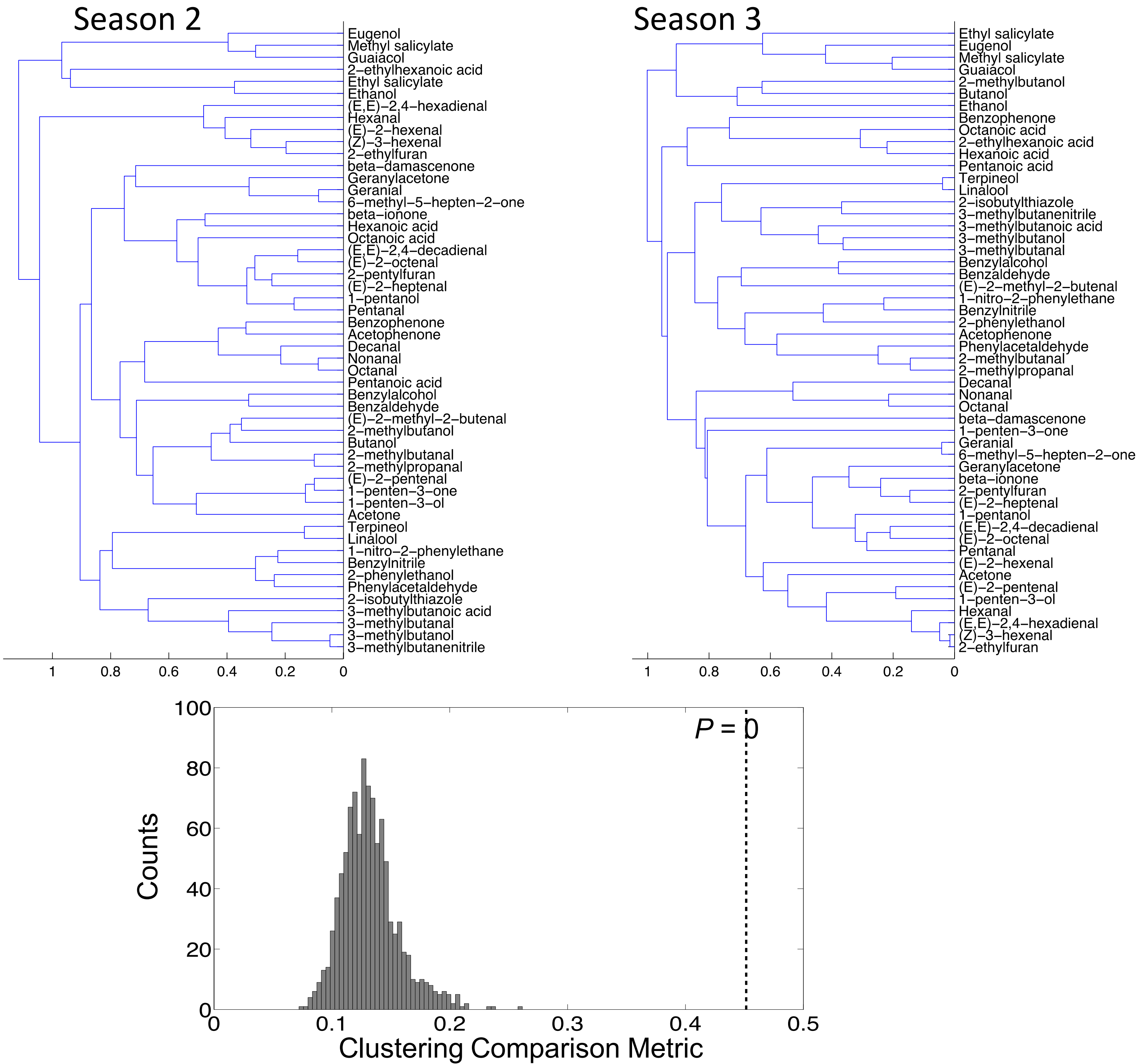
Season 2
Eugenol
Methyl salicylate
Guaiacol
2−ethylhexanoic acid
Ethyl salicylate
Ethanol
(E,E)−2,4−hexadienal
Hexanal
(E)−2−hexenal
(Z)−3−hexenal
2−ethylfuran
beta−damascenone
Geranylacetone
Geranial
6−methyl−5−hepten−2−one
beta−ionone
Hexanoic acid
Octanoic acid
(E,E)−2,4−decadienal
(E)−2−octenal
2−pentylfuran
(E)−2−heptenal
1−pentanol
Pentanal
Benzophenone
Acetophenone
Decanal
Nonanal
Octanal
Pentanoic acid
Benzylalcohol
Benzaldehyde
(E)−2−methyl−2−butenal
2−methylbutanol
Butanol
2−methylbutanal
2−methylpropanal
(E)−2−pentenal
1−penten−3−one
1−penten−3−ol
Acetone
Terpineol
Linalool
1−nitro−2−phenylethane
Benzylnitrile
2−phenylethanol
Phenylacetaldehyde
2−isobutylthiazole
3−methylbutanoic acid
3−methylbutanal
3−methylbutanol
3−methylbutanenitrile
1
0.8
0.6
0.4
0.2
0
Season 3
Ethyl salicylate
Eugenol
Methyl salicylate
Guaiacol
2−methylbutanol
Butanol
Ethanol
Benzophenone
Octanoic acid
2−ethylhexanoic acid
Hexanoic acid
Pentanoic acid
Terpineol
Linalool
2−isobutylthiazole
3−methylbutanenitrile
3−methylbutanoic acid
3−methylbutanol
3−methylbutanal
Benzylalcohol
Benzaldehyde
(E)−2−methyl−2−butenal
1−nitro−2−phenylethane
Benzylnitrile
2−phenylethanol
Acetophenone
Phenylacetaldehyde
2−methylbutanal
2−methylpropanal
Decanal
Nonanal
Octanal
beta−damascenone
1−penten−3−one
Geranial
6−methyl−5−hepten−2−one
Geranylacetone
beta−ionone
2−pentylfuran
(E)−2−heptenal
1−pentanol
(E,E)−2,4−decadienal
(E)−2−octenal
Pentanal
(E)−2−hexenal
Acetone
(E)−2−pentenal
1−penten−3−ol
Hexanal
(E,E)−2,4−hexadienal
(Z)−3−hexenal
2−ethylfuran
1
0.8
0.6
0.4
0.2
0
P = 0
Counts
100
80
60
40
20
0
0
0.1
0.2
0.3
0.4
0.5
Clustering Comparison Metric

Figure S4. Hierarchical cluster of the RILs according to their volatile profile in seasons 2 and 3 together. Log_2 transformed values for each volatile are represented as a heatmap. High levels are shown in red; low levels in green; intermediate levels in black.

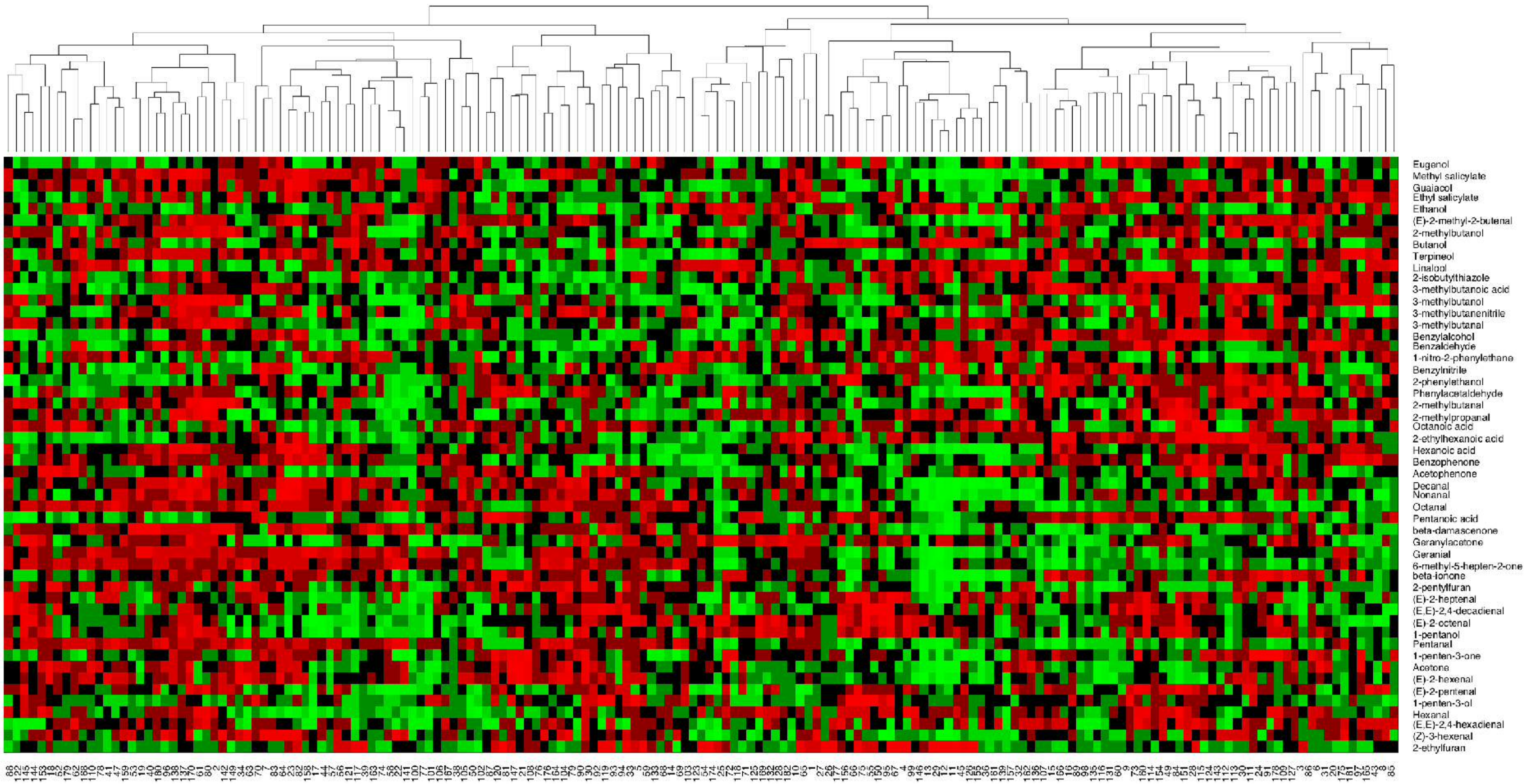
Eugenol
Methyl salicylate
Guaiacol
Ethyl salicylate
Ethanol
(E)-2-methyl-2-butenal
2-methylbutanol
Butanol
Terpineol
Linalool
2-isobutylthiazole
3-methylbutanoic acid
3-methylbutanol
3-methylbutanenitrile
3-methylbutanal
Benzylalcohol
Benzaldehyde
1-nitro-2-phenylethane
Benzylnitrile
2-phenylethanol
Phenylacetaldehyde
2-methylbutanal
2-methylpropanal
Octanoic acid
2-ethylhexanoic acid
Hexanoic acid
Benzophenone
Acetophenone
Decanal
Nonanal
Octanal
Pentanoic acid
beta-damascenone
Geranylacetone
Geranial
6-methyl-5-hepten-2-one
beta-ionone
2-pentylfuran
(E)-2-heptenal
(E,E)-2,4-decadienal
(E)-2-octenal
1-pentanol
Pentanal
1-penten-3-one
Acetone
(E)-2-hexenal
(E)-2-pentenal
1-penten-3-ol
Hexanal
(E,E)-2,4-hexadienal
(Z)-3-hexenal
2-ethylfuran
